# Divergent patterns between phenotypic and genetic variation in Scots pine

**DOI:** 10.1016/j.xplc.2020.100139

**Published:** 2020-12-29

**Authors:** David Hall, Jenny Olsson, Wei Zhao, Johan Kroon, Ulfstand Wennström, Xiao-Ru Wang

**Affiliations:** 1 Department of Ecology and Environmental Science, Umeå Plant Science Center, Umeå University, Umeå, Sweden; 2 Advanced Innovation Center for Tree Breeding by Molecular Design, College of Biological Sciences and Technology, Beijing Forestry University, Beijing, China; 3 The Forestry Research Institute of Sweden (Skogforsk), Uppsala Sweden

**Keywords:** clinal variation, cold hardiness, genetic diversity, population structure, *Pinus sylvestris*

## Abstract

In boreal forests, autumn frost tolerance in seedlings is a critical fitness component because it determines survival rates during regeneration. To understand the forces that drive local adaptation in this trait, we conducted freezing tests in a common garden setting for 54 *Pinus sylvestris* (Scots pine) populations (>5000 seedlings) collected across Scandinavia into western Russia, and genotyped 24 of these populations (>900 seedlings) at >10 000 SNPs. Variation in cold hardiness among populations, as measured by *Q*_*ST*_, was above 80% and followed a distinct cline along latitude and longitude, demonstrating significant adaptation to climate at origin. In contrast, the genetic differentiation was very weak (mean *F*_*ST*_ 0.37%). Despite even allele frequency distribution in the vast majority of SNPs among all populations, a few rare alleles appeared at very high or at fixation in marginal populations restricted to northwestern Fennoscandia. Genotype–environment associations showed that climate variables explained 2.9% of the genetic differentiation, while genotype–phenotype associations revealed a high marker-estimated heritability of frost hardiness of 0.56, but identified no major loci. Very extensive gene flow, strong local adaptation, and signals of complex demographic history across markers are interesting topics of forthcoming studies on this species to better clarify signatures of selection and demography.

## Teaser

Strong local adaptation in autumn frost tolerance was detected in *Pinus sylvestris* across northern Europe following the length of the growing season. In contrast, most of the genetic variation could be attributed to the expected migration route almost perpendicular to the gradient in autumn frost tolerance across the sampled distribution range.

## Introduction

Most boreal forest species have wide distribution ranges within which ecotype-specific variation and adaptive clines potentially arise across heterogeneous environments. Clinal trait variation is a response to spatially varying selection along environmental gradients. Strong local adaptations have been observed among tree populations in a plethora of published common garden experiments (e.g., Rehfeldt, 1989; Shutyaev and Giertych, 1998; Alberto et al., 2013), which identify a wide array of traits that affect fitness. For local adaptation to occur in species with large distribution ranges and extensive gene flow, such as Scots pine (*Pinus sylvestris* L*.*), selection has to be strong enough to overcome the homogenizing force of gene flow (Savolainen et al., 2007).

Selection on trait variation along environmental gradients is expected to generate allele frequency clines at loci that control a trait. However, distinguishing the impacts of local adaptation on allele frequency variation from those imposed by neutral evolutionary forces is often a challenging task in boreal settings and particularly for conifer trees (Zhao et al., 2020). Neutral forces have certainly acted on boreal conifer species when they tracked the receding ice sheets after the Last Glacial Maximum (LGM) and recolonized northern regions from their southern ice age refugia. Such migration routes expose migrating individuals to environmental clines that change with latitude. This confounds isolation by distance (IBD) (Wright, 1943) with isolation by environment and colonization (Wang and Bradburd, 2014), making inferences about the impacts of neutral and selective forces difficult to draw. In addition, selection on standing variation in quantitative polygenic traits may require only minute allele frequency shifts to facilitate local adaptation (Csilléry et al., 2018). This means that even if selection on a trait is strong, the identification of adaptive changes in minor allele frequencies associated with the trait variation is difficult, especially if the underlying genetic structure is unknown (Latta, 1998; Le Corre and Kremer, 2003; Hall et al., 2007; Yeaman, 2015). An advance in the field will likely come from coordinated investigations at both the phenotypic and the genomic levels with comprehensive sampling and a systems approach.

Scots pine is one of the most widely distributed conifers in the Northern Hemisphere (San-Miguel-Ayanz et al., 2016). The widespread occurrence of the species demonstrates an ability to adapt over spatially heterogeneous environments, e.g., large temperature and photoperiod variations (Rehfeldt et al., 2002; Jankowski et al., 2017). A clear illustration of local adaptation is that northern populations set buds and develop autumn frost tolerance significantly earlier than southern populations (Hurme et al., 1997; Andersson and Fedorkov, 2004; Savolainen et al., 2004; Jankowski et al., 2017). Autumn frost tolerance, “cold hardiness” hereafter, is a critical fitness component in the northern climate as it determines the mortality and survival of seedlings and thus the success rates of forest regeneration. The underlying genetic bases of these trait variations are still poorly investigated.

Scots pine likely recolonized Scandinavia after the LGM from the south and the Russian Plain (Bennett et al., 1991; Cheddadi et al., 2006; Tóth et al., 2017). However, macrofossil and ancient DNA evidence suggests that several tree species arrived in the north remarkably soon after the deglaciation and thus points to the possible existence of cryptic refugia on the edges of the ice sheet at high latitudes (Kullman, 2008; Parducci et al., 2012; Zale et al., 2018). This hypothesis would imply that a discrete genetic cluster of Scots pine may be present in the north. A complex demographic history can contribute to genetic heterogeneity over the distribution and complicate the inferences of other evolutionary processes in generating spatial diversity. The population genetics literature on Scots pine is extensive (see Pyhäjärvi et al., 2020, for review and literature therein). Despite these continuous efforts, large-scale sampling for phenotypic and genetic variation across the distribution is still lacking. It has also not been determined whether a fine-scale population structure exists in Scandinavia as a result of migration from the various refugia. This knowledge would inform us about the past history and refine both the detection power and the conclusions drawn from genome-wide associations (Barton et al., 2019).

In this study, we sampled 54 Scots pine populations from the Norwegian coast over the Arctic Circle to western Russia, covering 47.3 longitudes or more than 1/8 of the earth's circumference, which represents the most comprehensive coverage of northern Europe to date. We inferred variation in autumn phenology and dormancy progression from freeze-hardiness tests conducted on >5000 seedlings, of which >900 seedlings from 24 populations were genotyped using genotyping by sequencing (GBS). Our main goal was to evaluate adaptive responses in Scots pine at the phenotype and genotype levels. Evaluation of cold hardiness along environmental and geographical gradients would contribute to an understanding of the performance of these gradients for predicting freeze-damage levels. The genotype data allow evaluation of genetic variance across landscapes and thus shed light on the degree of genetic–environmental association and the recolonization history of Scots pine in Scandinavia. We found patterns in the allele frequencies that parallel the freeze-damage levels, although the generally small allele frequency shifts observed across the range could not be separated in attribution between geographic and environmental variables. More extensive investigations may provide further insights into the genomics of adaptive trait variation and aid the projection of evolutionary responses to climate change.

## Results

### Cold-hardiness variation

**Figure 1:**
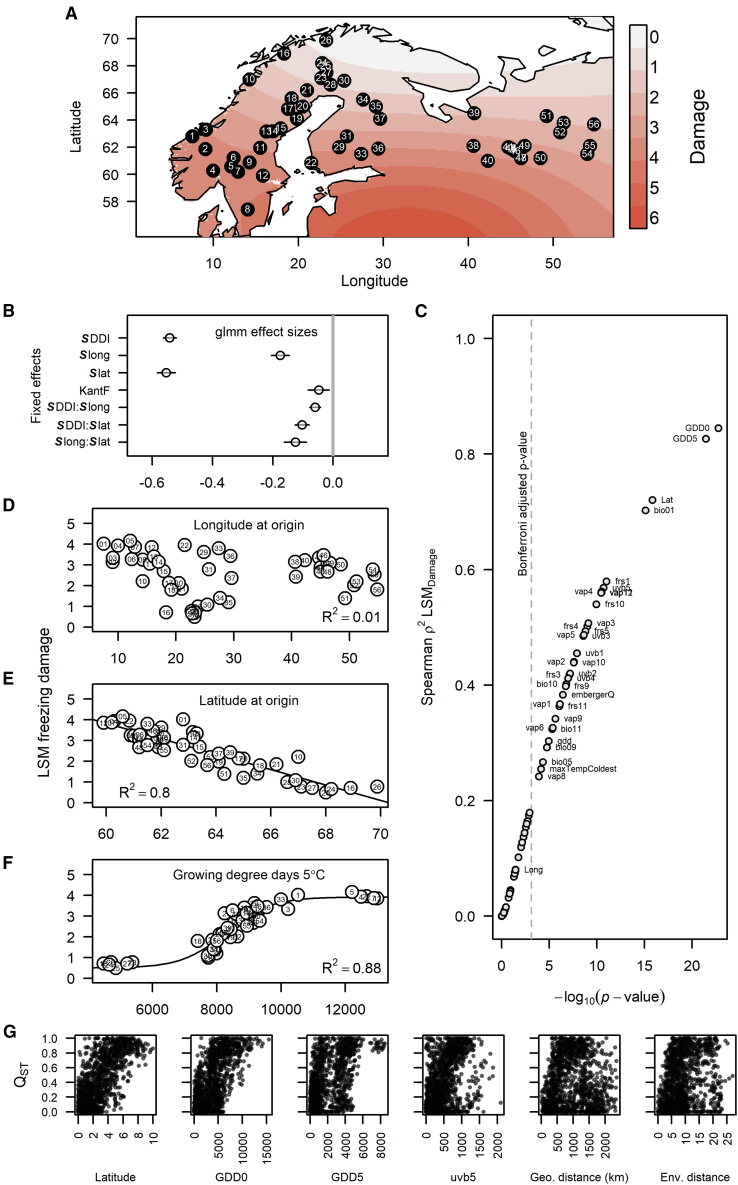
Phenotypic variation and environmental correlations. **(A)** The fitted damage level surface across the sampled range based on simple kriging of the least-squares means estimates of population damage levels from the GLMM model. **(B)** Estimated sizes of significant fixed effects from the GLMM model on freeze damage with their 95% CI. DDI, days since dormancy initiation; lat, latitude; long, longitude; and KantF, position of the seedling in the freezer. Uppercase ***S*** preceding effect names indicates that variables have been standardized before analysis. **(C)** Squared Spearman's ρ between least-squares means of damage levels and climate variables (see Supplemental Table 2), including latitude and longitude. **(D–F)** The explanatory ability of **(D)** longitude, **(E)** latitude, and **(F)** growing degree days above 5°C for freeze-damage variation among populations. **(G)** Pairwise *Q*_ST_ versus pairwise differences in latitude, growing degree days above 0°C (GDD0), GDD5, uvb5 (the four variables that correlate best with pairwise *Q*_ST_), and physical and environmental distance between populations.

The two major goals of this study were to understand the distributions of: (1) cold hardiness and (2) genotypic variances across the northwestern (NW) distribution range of Scots pine (Figure 1A). To achieve the first goal, we raised more than 5000 seedlings (Supplemental Table 1) in a common garden setting. We subsequently subjected these seedlings to increasing night length to initiate dormancy and finally exposed them to freezing temperatures to assess their frost hardiness through their damage levels.

The damage levels (0–6) of seedlings from each population were assessed at 10 specific time points after dormancy initiation. Because damage was scored categorically, we assumed a Poisson-distributed response variable and fit it using a general linear mixed model (GLMM) (see Materials and methods). The final GLMM showed that latitude at origin and number of days since initiation of dormancy (DDI) had the largest impacts on freeze damage (Figure 1B). Because DDI, latitude, and longitude were standardized (see Materials and methods), the effect size estimates do not translate 1:1 to the actual impact they had on damage levels; +1 in standardized latitudes decreases damage by 0.55 on average and a unit increase in standardized DDI decreases damage by 0.54 (Figure 1B), which corresponds to an average reduction of the damage scores by 0.22 for every +1°N and 0.06 for every additional day of dormancy progression. Longitude also had an effect where an increase in standardized longitude decreased damage levels by 0.18 on average (Figure 1B), which translates to a 0.012 damage reduction for every longitudinal degree east. Slightly higher damage scores were observed along the Norwegian coast at all latitudes, leading to a negative interaction between latitude and longitude (Figure 1A). This likely reflects the transition from the milder, Gulf Stream-influenced, maritime climates at higher latitudes along the Atlantic coast toward the increasingly continental climates of the east, where the onset of seasonal change is more rapid. We also detected a small position effect of the seedlings in the freezer (KantF): seedlings farther from the edge (i.e., more protected from freezing by neighboring seedlings) had reduced damage. Both longitude and latitude interacted negatively with DDI (Figure 1B), indicating a more rapid cold-tolerance progression with increasing longitude or latitude. All the above-described effects are highly significant from zero (Figure 1B), with p < 10^−10^, except for KantF, which had a p value of 0.0073.

Latitude is expected to have a high correlation with the length of the growing season and is often used as a proxy for climate in boreal settings. However, because longitude was also a significant factor, we expected that the actual climate at origin would be a better predictor of freeze damage (Andersson and Fedorkov, 2004). We then examined the statistical relationship of 68 climate variables (Supplemental Table 2) with freeze damage. In addition to latitude, growing degree days above 0°C (GDD0) and above 5°C (GDD5) and annual mean temperature (bio01) correlated well with the variation in freeze damage, but longitude did not (Figure 1C and 1D). In this study, the freezing time points were optimized to give the highest resolution in freeze damage around the average populations. This means that the resolution among the most extreme populations is reduced, and both extremes of the hardiness distribution level off, producing a response that is more sigmoidal than linear. Both GDD0 and latitude appeared to follow a more linear trend. When the data were fit to a sigmoid distribution, GDD5 (non-linear adjusted R^2^ = 0.883) explained more of the variance in freeze damage than either latitude (linear adjusted R^2^ = 0.805) or GDD0 (non-linear adjusted R^2^ = 0.853, data not shown; Figure 1E and 1F).

The principal-component analysis (PCA) on all 68 climate variables reduced the environmental space to three dimensions that explained 80.2% of the environmental variance among populations. The Norwegian coast appeared to possess unique environments resulting in greater environmental distances to all other populations (Supplemental Figure 1). In line with the strong clinal variation across latitude, GDD5, GDD0, and bio01, we observed clear divergent selection among populations on cold hardiness, with a point estimate of global *Q*_ST_ = 0.82 (95% highest [posterior] density interval [HPDI] 0.51–0.95). Pairwise *Q*_ST_ among populations correlated best with latitude, GDD0, and GDD5 (Spearman's ρ = 0.72, 0.69, and 0.6, respectively), but not with the overall environmental distance, longitude, and geographic distance *per se* (ρ = 0.31, 0, and 0.13, respectively, Figure 1G).

### Genetic diversity

To achieve our second goal, we genotyped 941 seedlings from 24 selected populations (Supplemental Table 1) using GBS. The average number of reads per individual was 2.36 M with a sequence depth 87×, and a mapping rate of 96.36% to the reference genome *Pinus taeda* (Supplemental Table 3)*.* We first screened for highly related individuals in the samples because their presence can inflate population structure. We identified 195 individuals with a relatedness greater than or equal to first cousin (Supplemental Table 4). Removing the related and replicated individuals and re-performing SNP filtering (see Materials and methods) gave us 855 487 SNPs. After removing non-polymorphic loci we had 85 573 SNPs left that, with a minor allele frequency <0.05 filter, shrank down to the final number of 10 925 SNPs for the remaining 746 individuals (Table 1). This set of individuals and SNPs was then used in diversity and population structure analyses.

**Table 1:** Summary of genetic diversity.

Population ID	Country	N	*H*_o_	*H*_e_	*F*_IS_	Nucleotide diversity π		
						All sites	Zero-fold sites	Four-fold sites
1	Norway	38	0.2683	0.2811	−0.0570	0.0045	0.0035	0.0065
2	Norway	18	0.2321	0.2931	0.0783	0.0044	0.0035	0.0064
3	Norway	40	0.2619	0.2791	−0.0163	0.0044	0.0035	0.0064
4	Norway	34	0.2729	0.2827	−0.0129	0.0045	0.0035	0.0066
8	Sweden	46	0.3026	0.2877	−0.1491	0.0050	0.0040	0.0076
10	Norway	49	0.2596	0.2798	−0.0159	0.0044	0.0035	0.0065
12	Sweden	56	0.2478	0.2795	0.0178	0.0045	0.0035	0.0064
13	Sweden	19	0.2338	0.2973	0.0608	0.0045	0.0036	0.0065
16	Norway	62	0.2288	0.2762	0.0330	0.0044	0.0035	0.0064
22	Finland	33	0.2517	0.2818	−0.0303	0.0044	0.0035	0.0066
26	Norway	59	0.2116	0.2780	0.0802	0.0044	0.0035	0.0064
30	Finland	20	0.2444	0.2903	0.0775	0.0045	0.0036	0.0064
31	Finland	16	0.2555	0.2982	0.0222	0.0044	0.0035	0.0065
33	Finland	10	0.2937	0.3181	−0.0213	0.0044	0.0034	0.0064
34	Finland	19	0.2764	0.2888	−0.0304	0.0045	0.0035	0.0064
37	Finland	64	0.2470	0.2780	0.0082	0.0044	0.0035	0.0065
38	Russia	15	0.2327	0.3018	0.0896	0.0044	0.0035	0.0063
39	Russia	12	0.2863	0.3201	−0.0888	0.0045	0.0036	0.0066
40	Russia	18	0.2662	0.2927	−0.0041	0.0044	0.0035	0.0064
47	Russia	20	0.2642	0.2886	0.0127	0.0045	0.0035	0.0066
51	Russia	19	0.2632	0.2935	−0.0334	0.0044	0.0035	0.0066
52	Russia	18	0.2507	0.2927	0.0272	0.0044	0.0035	0.0064
55	Russia	53	0.2567	0.2785	−0.0383	0.0044	0.0035	0.0065
56	Russia	8	0.3260	0.3356	−0.0483	0.0044	0.0034	0.0065
Overall		746	0.2487	0.2803	−0.0048	0.0045	0.0036	0.0066

Heterozygosities (*H*_o_ and *H*_e_) were measured as an average over all polymorphic sites in each population, whereas both monomorphic and polymorphic sites were used when calculating nucleotide diversity (π). Fixation index *F*_IS_ was calculated and tested by 1023 permutations of gene copies between individuals within each population. N is the number of seedlings remaining in each population after removing highly related individuals (see Supplemental Table 4 for distribution of related individuals).

The overall observed heterozygosity *H*_*o*_ was 0.2487, while expected heterozygosity *H*_*e*_ was 0.2803. All populations had similar values for *H*_*o*_ and *H*_*e*_ with no significant departures from Hardy–Weinberg proportions, as shown by the within-population fixation index *F*_IS_ (Table 1), in accordance with the high outbreeding of this species (Hall et al., 2020). The mean nucleotide diversity (*π*) for all sites was 0.0045 (Table 1), 0.0032 at zero-fold degenerate coding sites (*π*_*o*_), and 0.0066 at four-fold sites (*π*_*4*_). All populations had similar levels of diversity, except population 8 (southern Sweden), with slightly elevated estimates in both *H*_*o*_ and nucleotide diversity estimates*.*

### Population structure

**Figure 2:**
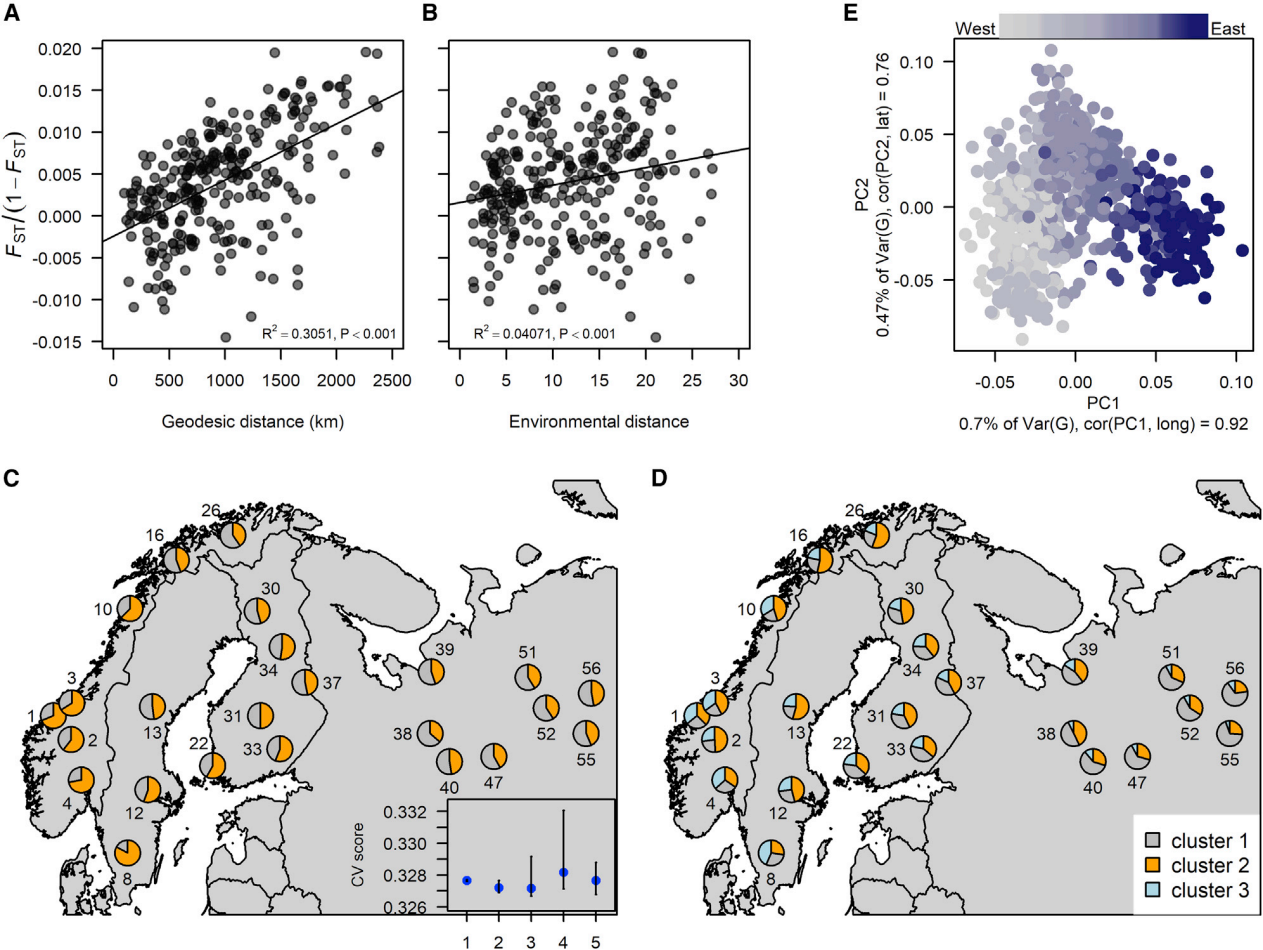
The spatial genetic structure across the genotyped populations using all SNPs. **(A** and **B)** The pairwise genetic differentiation across **(A)** geo-distance and **(B)** environment. **(C** and **D)** show the pie plots of genetic composition for *K* = 2 **(C)** and *K* = 3 **(D)** from *TESS3*. The blue points in the inlaid graph in **(C)** indicate the cross-validation score with the lowest root-mean-square error between the genotypic matrix and the fitted matrix among replications, for *K* = 1 to 5. **(E)** PCA of all genotyped seedlings, colored according to their longitudinal origin, with the darker color farther east. The two first PCA axes explained 1.17% of the genetic covariances among the seedlings. The PCA axes are also highly correlated with longitude (PC1) and latitude (PC2).

Using all 10 925 SNPs, we measured differentiation among populations using the fixation index (*F*_ST_)_._ Pairwise *F*_ST_ across all populations showed little variation and followed a normal distribution *N* (μ = 0.0040, σ = 0.0063, Supplemental Table 5), with a global estimate of 0.0037. Despite being low, *F*_ST_ increased with increasing geographic and environmental distances between populations (adjusted R^2^ = 0.31 and R^2^ = 0.041, respectively, both with *P* < 0.001, Figure 2A and 2B), indicating a pattern of IBD. Because of this IBD signal, we investigated whether we could detect possible ancestry components (*K*) over the sampling space using TESS3 (Caye et al., 2016). TESS3 suggested an optimal *K* from 1 to 3, although increasing *K* above 1 had only a marginal effect. It appears that at *K* = 3, the Russian populations displayed an ancestry composition that differed from the Fennoscandian populations (Figure 2C and 2D). We further examined population structure using c*onStruct*, which considers both continuous and discrete processes. Cross-validation tests indicated that the spatial model that accounts for IBD is preferred over the discrete non-spatial model, and that one ancestral component is broadly sufficient to describe the global structure in our data (Supplemental Figure 2).

PCA of the overall distribution of genetic variation revealed a similar pattern with a generally low discrimination power among populations but a discernable trend along longitude (PC1, R^2^ = 0.85) and latitude (PC2, R^2^ = 0.58, Figure 2E). Only 1.17% of the genetic variation was explained by the first two PC axes. All these results suggest a very weak population structure in Scots pine across Fennoscandia and western Russia.

**Figure 3:**
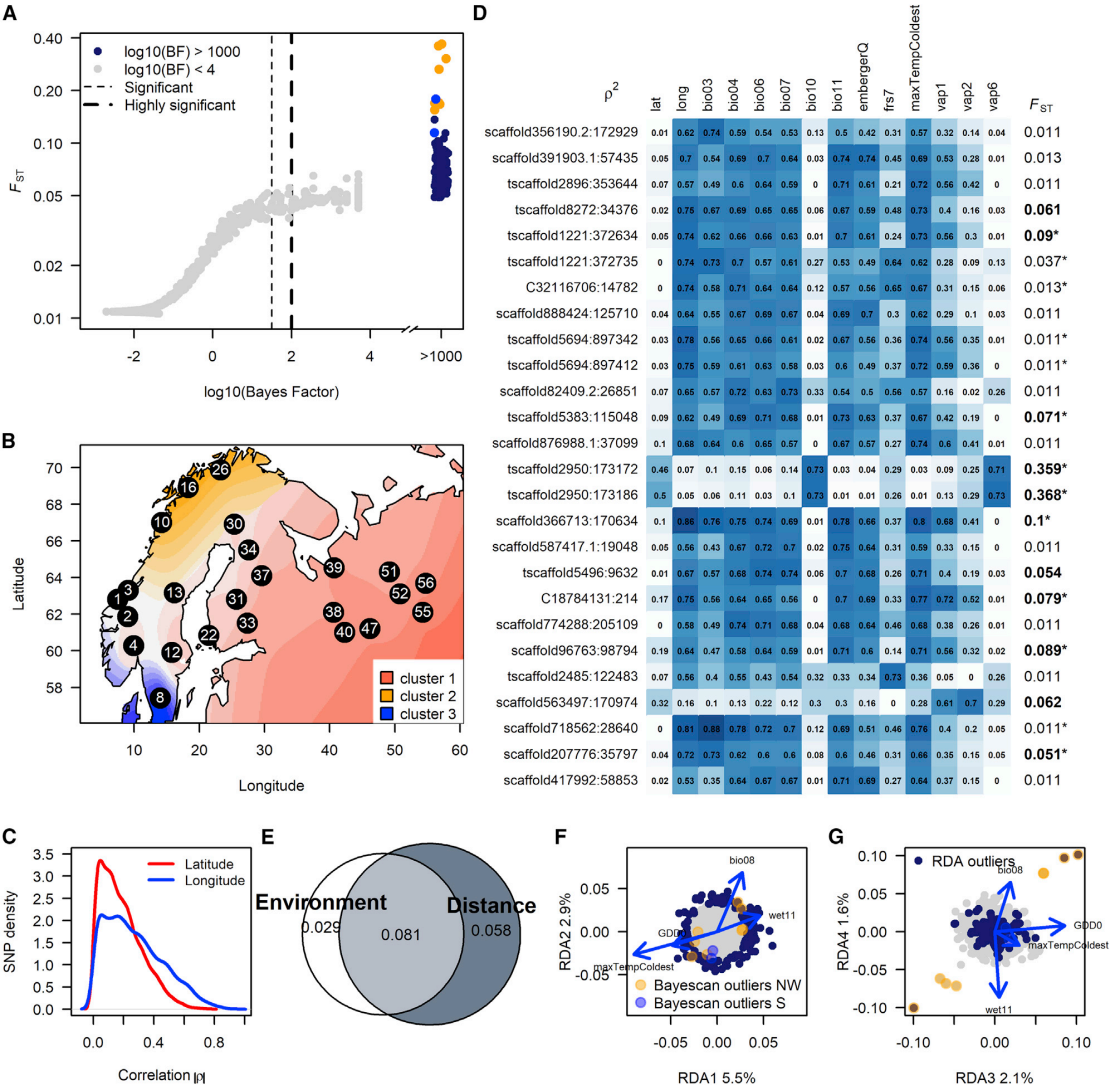
Detection of highly differentiated loci across the sampled range and environments. **(A)** BayeScan detected 164 outlier loci with a log_10_(Bayes Factor) ≥ 1000 and *F*_ST_ values ranging from 0.049 to 0.368. The highly differentiated loci specific to NW populations are marked in orange and the outliers in the southern cluster in blue. The *y* axis is on a logarithmic scale. **(B)** Distribution of major ancestry components in the 164 BayeScan outlier loci. **(C)** Distribution of absolute allele frequency Spearman's rank correlations (|ρ|) with latitude (red line) and longitude (blue) across all loci. **(D)** Squared correlation plot of 26 SNP and 12 environmental variables that had a ρ^2^ > 0.7; latitude and longitude are included for reference. *F*_ST_ values in bold are significant outliers in BayeScan; those with an asterisk are outliers in the environmental RDA model. **(E)** Proportion of variance explained by the environmental versus the geographic distance matrix in the full RDA model (see Materials and methods). **(F** and **G)** RDA of population allele frequencies in response to the most influential environmental variables in the first four constrained axes, of which **(F)** RDA1 and RDA2 are significant. **(G) RDA3 and RDA4.** Midnight blue points are outliers. However, no outliers were detected when geographic distance was accounted for.

To explore the possible forces on differentiated loci, we searched for significant *F*_ST_ outliers using BayeScan (Foll and Gaggiotti, 2008) and TESS3 (Caye et al., 2016), which control for geographic relationships between populations. Given the overall low differentiation, BayeScan identified 164 highly significant loci with a probability of 1 of being differentiated (*F*_ST_ > 0.0491; Bayes factor > 1000; Figure 3A), while TESS3 detects no significant outliers. These contrasting results imply that geographic relationships between populations are an important factor in the differentiation detected by BayeScan. Further examination of the allele frequency origins of these outliers indicated three major underlying haplotype components with a finer-scaled structure among populations. One cluster was of eastern origin, the second cluster was centered in NW Norway, while the alleles for the third cluster appeared only in southernmost Sweden (Figure 3B). The main drivers of this clustering are likely centered on the apparent distinct allele frequency differences among regions (Supplemental Figure 3). The southern cluster had a high frequency of one of the alleles at each of two loci (these loci are colored blue in the figures), while all other populations were almost fixed for the other allele. The same is true for eight other loci (colored orange) in the three NW populations. There are no apparent relationships between the ancestral components in outliers and the geographical variation in autumn frost tolerance (Figure 1A).

### Genetic–environmental association

To understand whether allele frequency variation could be associated with environmental variation, we compared allele frequencies among populations with their latitude, longitude, and climate variables using direct correlations and redundancy analysis (RDA). The overall allele frequencies were most strongly correlated with longitude and weakly with latitude (Figure 3C). Rank (Spearman) correlations among the allele frequencies of all loci to all 68 climate variables identified 26 loci that showed elevated correlations with 12 environmental variables (Figure 3D).

For RDA, we first performed forward selection on the initial 68 environmental variables and the principal coordinates of neighbor matrices (PCNM) separately. This step selected bio8 (mean temperature of wettest quarter), GDD0, and wet11 (wet day frequency in November) for the environmental matrix and four PCNM axes (1, 5, 11, and 13) for the geographic matrix. The full RDA model with these eight variables was significant and explained 17.4% of the total variance observed among populations (adjusted R^2^ = 17.4%, *P* = 0.001). Partitioning the variances for environmental and geographic impacts revealed a confounding effect of 8.1% between the two groups of factors, and environmental variables alone explained 2.9% and geo-distance 5.8% of the variation among populations (Figure 3E).

RDA performed on the environmental matrix alone identified the first two RDA axes as significant (*P* = 0.001 and 0.006) and explained about 5.5% and 2.9% of the variance, respectively. Maximum temperature of the coldest month is the most influential variable, with a correlation of −0.90 with RDA1. This variable had, in turn, a large negative correlation with longitude, Spearman's *ρ* = −0.88. Outlier analysis on the two significant RDA axes detected 154 significant SNPs, of which 38 were among the 164 BayeScan outliers (Figure 3F and 3G). Of the 38, 13 also showed high allele frequency correlations with environmental variables (Figure 3D). The strongly differentiated NW outliers, on the other hand, were mainly along the fourth non-significant RDA axis (Figure 3F and 3G), indicating that some environmental factors might be linked to their allele frequency shifts, but the signals are weak. Furthermore, no significant loci were detected when controlling for geographic distance in partial RDA, demonstrating that environmental and geo-distance are confounded in their influence on allele frequencies. The fact that RDA on the environment detected numerous outlier loci and that environmental factors by themselves explained about 3% of the allele frequency shifts implies that there could be adaptive differentiation among loci in our dataset, although pinpointing the environmental variables shaping their allele frequency distribution and disentangling them from the geographic relationships would be difficult.

### Association mapping

**Figure 4:**
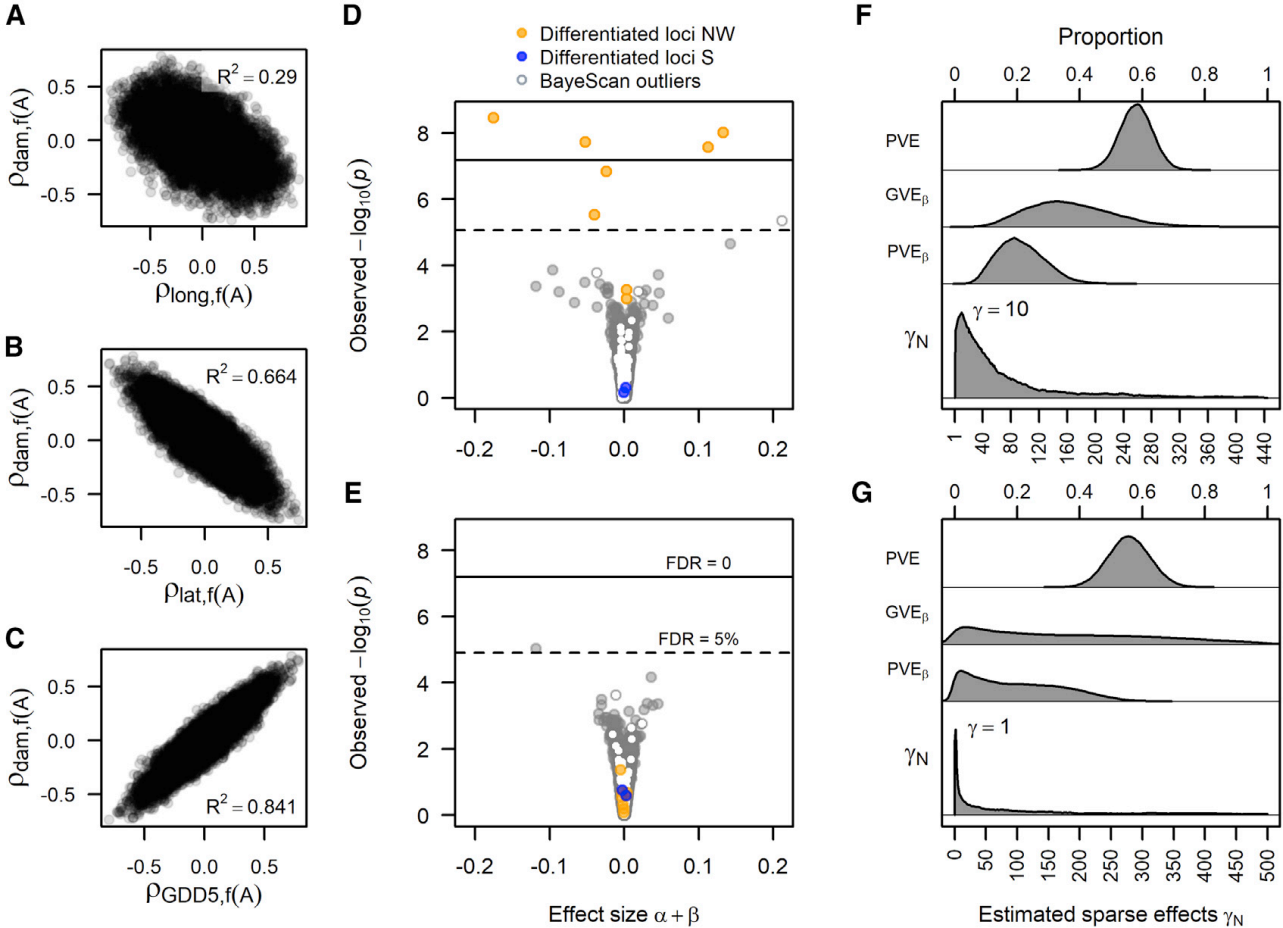
Allele frequency correlation with damage levels and genotype association with damage levels and environmental variables. **(A–C) (A)** The allele frequency rank correlation relationship between longitude–allele correlation and damage–allele correlation. **(B)** Latitude–allele correlation versus damage–allele correlation and **(C)** GDD5 and damage. Association mapping was performed on two datasets, the full population set and one excluding the NW populations. **(D** and **E)** Association mapping with damage levels for the **(D)** full and **(E)** reduced datasets. SNP effect size was estimated from BSLMM for the small effect size (*α*) found in each SNP and the major effect detected for some markers (*β*). *P* values and false discovery rate (FDR) are from the LMM. Colored points (orange, blue, and white) are the 164 BayeScan outlier loci, of which orange points are those unique to NW populations (10, 16, and 26) and blue are those unique to the southern population (8). **(F** and **G) (F)** The posterior distribution of the estimated genetic effects (top axis) and number of major effects (*γ*_N_, bottom axis) from the MCMC runs in the BSLMM for the full and **(G)** reduced datasets. PVE is the estimated marker heritability.

We found strong indications of footprints in the allele frequency gradients that mirror adaptation to cold hardiness, and both BayeScan and RDA indicated the possibility that some loci have been under selection. For example, those alleles that correlated strongly with freeze damage scores also correlated with latitude and the length of growing season (GDD5), but not with longitude (Figure 4A–4C), which otherwise appears to drive most allele frequency shifts. One of these loci, scaffold 563497:170974, displayed the highest explanatory power with freeze damage (linear model fit: R^2^ = 0.61, adjusted R^2^ = 0.596. Supplemental Figure 4).

To explore the possible association between genotypes and hardiness, we ran association mapping on seedlings' quantile normalized damage scores for two datasets: the full set of 24 populations and a second set without the NW populations 10, 16, and 26. The univariate linear mixed model (LMM) (Zhou and Stephens, 2012) in GEMMA (Genome-wide Efficient Mixed-Model Analysis) identified a group of highly significant loci in the full dataset and some with elevated effect size based on the Bayesian sparse linear mixed model (BSLMM) (Zhou et al., 2013, Figure 4D). Interestingly, most of these significant loci were the NW outliers. When the NW populations were removed, the signals of association disappeared, and none of the BayeScan outliers were significant or had elevated effect sizes (Figure 4E). Despite the lack of major effect loci in the reduced dataset, the marker estimated heritabilities of hardiness (*PVE*) were similar, *h*^2^ = 0.58 and 0.56, in the full and reduced population sets, respectively (Figure 4F and 4G, and Supplemental Table 6). This indicates that the combined minor effect size of all loci in the study captured a majority of the additive genetic variance in the trait. We also observed that the major genetic effects contributed around 19% (HPD point estimate *PVE*_*β*_) of the phenotypic variance in the full dataset. However, the sparse effect estimates (*GVE*_*β*_) showed no convergence in the reduced population set, as indicated by the large HPD interval and a point estimate close to zero (Figure 4G and Supplemental Table 6). This further suggests that there are no clear major genetic signals from any marker and that the loci detected as significant when NW populations were included are likely due to a large confounding effect between their extreme allele frequency shifts and their extreme environment.

Overall, our association results suggest that the markers captured a large part of the genetic variance in autumn frost tolerance among samples, but the identification of any single locus with identifiable effects on the trait is at best ambiguous. The small number of pseudo-randomly selected markers used in this study, compared with the genome size of Scots pine, makes it unlikely that we would catch any true large effect markers.

## Discussion

### Cold-hardiness variation and local adaptation

Quantitative and qualitative assessment of local adaptation is an essential step toward understanding the major evolutionary forces operating in natural populations. The common approach is to establish the relationships between phenotype, genotype, and environment and their interactions under (semi)controlled conditions to identify the selective agents underlying the trait variation. Estimating the degree of local adaptation through phenotypic measurement is often an extensive endeavor, especially for species with long generation times and vast distribution ranges. In forestry, cold hardiness of seedlings guides their deployment to correct climate zones and thus has operational cost–gain consequences. This is particularly true in northern Scandinavia, where the most important trait for production forests is survival (Berlin et al., 2009; Persson et al., 2010). Autumn frost hardiness in young seedlings has an estimated narrow sense heritability of 0.3–0.54 (Persson et al., 2010) and is an important target in breeding programs for northern climates (Andersson, 1992).

We observed increased and more rapid progression of frost tolerance in populations from harsher climates and a high estimate of *Q*_ST_ 0.82 among the sampled populations, illustrating strong divergent selection on this trait. If we account for the marker-estimated heritability of 0.56, which is somewhat higher than previously reported, the global phenotypic differentiation would be even higher, *Q*_ST_ = 0.9 (95% HPDI 0.65–0.97, see Materials and methods). High *Q*_ST_ values for phenology-related traits have also been reported for other tree species, e.g., Norway spruce (Milesi et al., 2019), downy birch (Bennie et al., 2010), European beech (Strømme et al., 2019), and European aspen (Hall et al., 2007). Analysis of phenotype–environment associations with 68 climate variables identified mainly those that describe the temperature over the year, GDD5, GDD0, bio01, and latitude. Latitude at origin has often been used as an explanatory variable in studies of clinal phenotypes and allele frequencies, e.g., insects (Oakeshott et al., 1982), birds (Johnsen et al., 2007), and plants (Stinchcombe et al., 2004; Ma et al., 2010). However, we also observed significant differences in frost tolerance among populations from the same latitudinal origins, but no differences when comparisons were based on length of the growing season at origin. We found that the phenotypic estimates and interactions with climate variables are complex, and that a simple experimental design along a latitudinal gradient would not capture all interactions with frost hardiness. Additional aspects of this phenotypic variation become evident when measured over a larger range across a boreal setting, indicating that the usual proxies of climate are suboptimal.

### Spatial genetic variance

Linking phenotypes and environmental factors to genetic variation requires a correct estimate of population structure, even at finer spatial scales (Barton et al., 2019). Similar to the importance of analyzing phenotypes across the appropriate explanatory variables to facilitate the separation of confounding factors, the assessment of genetic pattern would suffer from limited spatial coverage. Although we had a gap in our sampling, from the Karelia and Murmansk regions between Finland and Archangelsk, we detected a gradual change in genetic composition from east to west, with one ancestral component predominant in Russia and one in Scandinavia. This is suggestive of a major migration route from east to west. However, accounting for IBD, the gradual change in allele frequencies could be explained by a continuous spatial model with one ancestral component. A global *F*_ST_ of 0.0037 is much lower than that in Norway spruce (*Picea abies*), which also has a large distribution and a postglacial migration history similar to that of Scots pine. In Norway spruce, strong genetic differentiation is observed between the northern and the central parts of Europe (*F*_ST_ = 0.15–0.22; Chen et al., 2019; Milesi et al., 2019), as well as at a regional scale in northern Europe (Tollefsrud et al., 2009; Tsuda et al., 2016; Li, 2020; Sullivan, 2020). Our observation of weaker population structure in Scots pine in northern Europe, with no distinct subgroups, is in accordance with previous studies of Scots pine population structure (Dvornyk et al., 2002; Pyhäjärvi et al., 2007; Zimmer and Sønstebø, 2018; Tyrmi et al., 2020).

The very high and uniform diversity across Scots pine populations implies effective gene flow over large distances, which in turn could mask possible footprints of admixture in a population with recurrent migration from several refugia. However, the slightly elevated nucleotide diversity in the southern Swedish population (Table 1) could indicate an admixture of gene flow from two migratory routes, similar to what is observed in Norway spruce (Chen et al., 2019). In contrast to hardiness variation, which is associated with latitude, we found that the factors that most strongly correlate with and shape allele frequencies are longitude and environmental variables that follow a longitudinal gradient. In line with this, we found that the genetic variance explained by environmental variables overlaps the variance explained by spatial variables to a large extent. Longitude explained almost 50% more of the genetic variance than latitude in the PCA (Figure 2E). This suggests that most neutral allele frequency shifts, albeit small, are due to a longitudinal migratory route together with effective admixture, possibly over multiple post-Ice Age recolonizations.

In contrast to the weak structure in the nuclear genome, mitochondrial DNA markers have shown distinct differentiation in Scots pine from western Europe to eastern Russia (Naydenov et al., 2007; Dering et al., 2017). Mitochondrial DNA markers are maternally inherited through seeds and can often reveal otherwise hidden demographic components due to the low dispersal ability of seeds (Pyhäjärvi et al., 2008; Semerikov et al., 2018). Unfortunately, the resolution so far is low from the detected mitochondrial variation in Scots pine, nonetheless two distinct mitotypes have been found in Fennoscandia (Dering et al., 2017), and the pattern is inconclusive as to whether a refugium existed in northern Scandinavia under the LGM (Kullman, 2008; Parducci et al., 2012). Interestingly, Parducci et al. (2012) recovered 20 000 year old ancient *P. sylvestris* DNA and macrofossils in lake sediments on the island Andøya, situated just west of our population 16. This finding indicated a possibility of recovering a genetic signal in current populations. To bridge this knowledge gap, we managed to obtain material from a large part of Norway, and in particular NW Norway, and scored a substantial number of nuclear markers. This expanded sampling allowed us to identify a greater number of differentiated loci than in other recent studies (e.g., Tyrmi et al., 2020). Further mapping of these loci provided hints of three major haplotype structures underlying their allele frequency differences. Although the signals are weak, the NW-most populations appear to harbor alleles from an isolated lineage with otherwise rare alleles at or almost at fixation (populations 10 and 16, respectively). Possible scenarios for this to occur are genetic drift or selection. Genetic drift would be more effective when gene flow into these marginal populations is restricted. However, for almost all loci the differentiation is basically zero, implying large gene flow among populations in general. For this drift scenario to be plausible, given the observed allele frequency distribution, the simplest explanation would be that some loci were located in a genomic region that experienced rearrangement during drift. This would produce a segment with greatly reduced recombination rate relative to all other populations. Thus, when the gene-flow barrier dissipated, the high frequencies would be maintained for a few loci, despite the high gene flow observed across the range since LGM, a corresponding time period of a few hundred or so generations. If selection, on the other hand, has shaped these extreme allele frequency differences, we would expect very strong selection favoring the rare allele in these populations, but equally strong against them in all other populations, given the extremely high gene flow observed. Unfortunately, we cannot examine these hypothesis further with the data currently at hand.

### Genotype–environment and genotype–phenotype associations

The aim of association studies is to understand the underlying causes and biological nature of the variation in traits so that this information can be used to predict responses to future conditions and facilitate selection in breeding. However, association mapping in the wild for polygenic traits is challenging due to confounding factors and complex genetic interactions, resulting in typically weak association and predictive power (Nadeau et al., 2016; Barton et al., 2019). The influence of genes and environment on important quantitative traits remains elusive in conifer species.

In this study, we detected 164 significant *F*_ST_ outlier loci. Some of the loci correlated strongly with both environmental variables and longitude, but none were significant in the genotype–phenotype mapping. A similar result is reported by Tyrmi et al. (2020), who obtained almost eight times as many SNPs from exome capture (although still a minor part of the genome), but detected only one significant outlier. The most extreme *F*_ST_ outliers in our study are limited to the Norwegian marginal NW distribution range. The same extreme outliers are the only loci that appear to have a significant effect on freeze damage, but the signal from these loci disappeared completely when the NW populations were excluded from the analysis. It is also striking that, compared with BayeScan, neither TESS3 nor RDA detected any outlier loci when accounting for geography. From this we conclude that it is more probable that none of the loci we analyzed are more strongly influenced by any environmental factor than as a result of colonization or that they individually control a substantial proportion of frost hardiness. However, the great number of shared allele frequency correlations between freeze damage and length of growing season, rather than the east–west colonization route, is also suggestive of the presence of many small allele frequency shifts that have been shaped by local adaptation. The high marker-estimated heritability of frost hardiness (*h*^2^ = 0.56) corroborates this hypothesis. This is a promising finding illustrating that, collectively, our markers captured a large portion of the trait variation and inheritance. The weak association signals at individual loci are not surprising, given the small and random portion of the genome sampled, the large effective population size of Scots pine, and the polygenic architecture of quantitative traits. In such cases, the statistical power of detecting any association is very limited unless the sampling of markers and phenotypes is extraordinarily large (Hall et al., 2016; Gienapp, 2020).

Overall, this study reveals several suggestive results that further studies may shed light upon: (1) The evolutionary forces that shape the strong correlation of some outliers with a locally adapted trait and climate should be studied. With an east–west colonization route almost perpendicular to the adaptive cline and the low neutral differentiation observed, the population genomics of Scots pine should facilitate detection of loci under divergent selection given a larger representation of the genome. (2) The three underlying haplotype structures among differentiated loci across the range (see Supplemental Figure 3, and Tyrmi et al., 2020) could have been caused by differential linkage disequilibrium between these loci in some populations. The fixation of otherwise rare alleles in the NW populations despite extensive gene flow indicates a reduction in recombination around these loci, possibly from an inversion, that would allow for these alleles to drift to fixation. Another possibility would be that differential selection on these or adjacent linked loci in a low recombination area brings these rare alleles to high frequencies. The common rare alleles in the southern population possibly originate from admixture with continental alleles, due to migration routes similar to those observed in *P. abies* (Chen et al., 2019). Whether this is the case or whether it is due to some other phenomena clearly needs further validation. (3) Finally, capturing a large proportion of genetic variance in the trait could potentially lead to genomic prediction in natural populations. However, whether we would actually then estimate the origin of the population as a function of the spatial matrix rather than the actual adaptation of the population to the local environment remains to be explored. Nevertheless, Scots pine promises to provide an excellent genomic background to advance our understanding of how allele frequency shifts are shaped by evolutionary forces.

## Materials and methods

A more detailed Materials and methods is provided in the Supplemental information.

### Sampling and freezing test

To obtain a comprehensive view of autumn frost-tolerance (or cold-hardiness) variation, genetic diversity and possible genetic structure of the NW distribution range of Scots pine, *P. sylvestris* L., we collected 54 populations ranging from Norway to western Russia and covering latitudes 57.5°N–69.1°N (Figure 1, Supplemental Table 1). Fifty-three of these populations were freeze tested. Seedlings were grown in a greenhouse at +20°C. Each seedling's position in the seedling box was mapped for calculation of edge effect (KantF). From week 10, the length of the dark period was increased by 1 h every week to initiate bud dormancy. Seedling boxes were placed in a freezer chamber at 10 different time points after the DDI treatment, for 2 h at −10°C. The freeze damage on each seedling was evaluated at least 1 week after to allow for discoloration to develop. The degree of freeze damage was scored visually into seven classes: 0, no needle discoloration; 1, 1%–20% of needles discolored; 2, 20%–40%; 3, 40%–60%; 4, 60%–80%; 5, 80%–99%, and 6, all needles discolored. This testing protocol was established in the early 1980s by the Forestry Research Institute of Sweden (Skogforsk) in Sävar (Andersson, 1992; Persson et al., 2010) as a standard method for monitoring the hardiness of Scots pine seed orchard crops for reforestation. Needles were collected from seedlings for genotyping before they were subjected to freeze exposure.

### Phenotypic analyses

Freeze damage was analyzed using a GLMM with the “glmer” function in the R package “lme4” (Bates et al., 2015). We assumed a Poisson-distributed response variable of damage categories as a function of the fixed effects of placement in the freezer (KantF), DDI, longitude, and latitude at origin, and their interactions. Individual plants were treated as a random effects of the population within replication. Longitude, latitude, and DDI were centered (subtracting the mean) and then scaled by dividing by their standard deviations. The average damage levels of the populations were calculated as the least-squares means for each sampled population to remove some experimental effects.

We also sampled the posterior distribution of freeze-damage differentiation among populations to estimate *Q*_ST_ in this trait with a Markov chain Monte Carlo (MCMC) procedure. *Q*_ST_ is the quantitative trait equivalent of the *F*_ST_ (Spitze, 1993; Whitlock, 1999; Leinonen et al., 2013). *Q*_ST_ for freeze damage was calculated as follows:

$$Q_{ST}=\frac{\sigma_{B}^{2}}{\sigma_{B}^{2}+2h^{2}\sigma_{W}^{2}},$$

where $\sigma_{B}^{2}$ is the between-population variance estimate and $\sigma_{W}^{2}$ is the within-population variance estimate (Spitze, 1993; Whitlock, 1999). The $\sigma_{W}^{2}$ term is confounded and contains the total within-population phenotypic variance, i.e., heritability, *h*^2^ = 1. We estimated pairwise *Q*_ST_ values between populations and compared them with their differences in environmental variables, latitude, longitude, and physical and environmental distances. To calculate environmental distance, we performed a PCA on 68 environmental variables (Supplemental Table 2) and used the decomposition of variables to calculate the Euclidean distance *D*_*i,j*_ between populations *i* and *j* in multidimensional space.

### GBS library preparations

We extracted DNA for seedlings from 23 populations (Table 1, Supplemental Table 1) using the EZNA SP Plant DNA Kit (Omega Bio-tek). The GBS library was prepared using a *Pst*I high-fidelity restriction enzyme (New England Biolabs), following the protocol of Pan et al. (2015). Briefly, 200 ng DNA from each seedling was digested separately and ligated to sequencing adapters (with individual barcodes) simultaneously. This was carried out at 37°C for 8 h followed by 65°C for 30 min. The digested and ligated DNAs of 300 samples were then pooled into each library, purified, and PCR amplified. Fragment sizes of 350–450 bp were selected using an E-gel EX 2% agarose gel (Thermo Fisher Scientific) and purified. Paired-end sequencing (2 × 150 bp) was performed on Illumina HiSeq X Ten. In each library, we included a few samples as within- and among-library replicates.

### Bioinformatics

Sequence read quality was assessed with FastQC (http://www.bioinformatics.babraham.ac.uk/projects/fastqc/). Adapter sequences and low-quality bases (Phred quality <20) from the tail of each read were removed by using Trimmomatic (Bolger et al., 2014). Clean reads were cataloged by using the process_radtags module of Stacks v.2.0 (Catchen et al., 2011) according to individual barcode. Reads shorter than 41 bases were discarded. Sequence reads were aligned to the *P. taeda* draft genome v.1.01 (Neale et al., 2014; Zimin et al., 2014) using the Burrows-Wheeler Aligner MEM algorithm with default parameters (Li, 2013). Variants were called using the SAMtools and BCFtools pipeline with default parameters (Li, 2011; Catchen et al., 2013). One previously genotyped population (No. 8) was included in this study, resulting in a total of 941 individuals from 24 populations in the final sequence dataset (Supplemental Table 1).

Several filtering steps were performed to minimize genotyping errors: SNPs located in repetitive regions (reference to *P. taeda* genome v.1.01) and with mapping quality <40 were removed; genotypes with genotype quality <20 or read depth <5 were masked as missing; loci with a missing rate of >30%, minor allele frequency of <5%, or heterozygosity of >70% or that were not biallelic were also removed.

### Genetic diversity and population structure

The presence of related individuals, if undetected, would inflate population structure. To remove highly related samples, we examined relatedness among individuals in each population following the procedure of Hall et al. (2020). To obtain an overview of the spatial pattern of diversity we examined population structure using the R implementation of TESS3 (Caye et al., 2016) with the assumptions of one to five ancestral populations (*K*) and each *K* replicated 20 times. To avoid overestimating the number of potential clusters caused by the presence of IBD, which is often found in continuous populations, we used *conStruct* v.1.03 (Bradburd et al., 2018) to identify structure in a spatially aware context. We tested both the spatial and the non-spatial models with *K* values from 1 to 5 and 50 000 MCMC iterations for each test and with 5000 iterations and 10 replicates for model cross-validation.

To examine whether the individual seedling genotypes could be classified into their respective populations and whether the genetic variation could be attributed to geographic distance, we performed a PCA on the genetic covariances with EIGENSOFT v.6.1.4 (Patterson et al., 2006; Price et al., 2006). Genetic differentiation among populations was determined using pairwise *F*_ST_ (Weir and Cockerham, 1984) in Arlequin 3.5 (Excoffier and Lischer, 2010), where the statistical significance was assessed by 1023 permutations and a significance level of 0.05.

We estimated the nucleotide diversity at four-fold degenerate, synonymous sites (π_4_) and zero-fold, non-synonymous sites (π_0_) using the general feature format (gff) annotation file of *P. taeda*. We calculated observed (*H*_o_) and expected (*H*_e_) heterozygosity and fixation index *F*_IS_ in each population and overall. Pairwise nucleotide diversity at all sites (π) and at zero-fold (π_0_) and four-fold (π_4_) degenerate coding sites were computed using VCFtools (https://vcftools.github.io/index.html).

### Genotype and environment association

As an initial screening of putative genotype–environment associations, we calculated the correlations between population-specific allele frequencies and their latitude, longitude, and GDD5 to identify possible allele frequency clines. We also compared the observed allele correlations among the three variables to establish whether there was an overlap between correlations.

To identify loci that are more or less differentiated than the average loci, we performed an *F*_ST_ outlier test of the SNPs using BayeScan (de Villemereuil et al., 2014; Foll and Gaggiotti, 2008). We also used TESS3 for *F*_*ST*_-outlier loci detection. This method examines allele frequency changes while taking geographical constraints into account. We further analyzed the BayeScan outliers using TESS3 to see whether they resulted from shared ancestral components or possibly similar or linked selection and whether those components could be attributed to specific geographic origins.

To evaluate the impact of environmental factors on the differentiation at all loci and among outliers, we performed RDA over population allele frequencies (as the dependent matrix) and environmental parameters (independent matrix), a method that has been shown to be robust in comparative studies for detecting genotype by environment associations (Forester et al., 2018). We included two independent matrices in RDA, an environmental matrix and a geo-distance matrix in the form of PCNM. We performed forward selection on 68 environmental variables (Supplemental Table 2) and on 15 PCNM decomposed geographic distance variables. The number of variables was reduced with a step-wise model to the four most influential variables in each dataset. We also ran partial RDA models, conditional on the geographic distance and environmental distance, respectively, to assess the exclusive impacts of env- and geo-factors on population differentiation and whether we could observe outlier SNPs exclusively shaped by the environment. Evaluation of SNP significance in the RDA was based on *anova.cca* function in the “vegan” R package (Oksanen et al., 2019). Significance tests followed the method in Capblancq et al., (2018).

### Association mapping with damage levels

To further examine genotype–phenotype associations, we applied more direct association mapping of genotypes to freeze-damage levels. The damage scores were normalized over replication and freezing time points with the quantile normalization procedure in the Bioconductor R package “preprocessCore” (Bolstad, 2020), which results in comparable phenotypes across replicates.

Association mapping with damage levels was performed using both the LMM (Zhou and Stephens, 2012) and the BSLMM (Zhou et al., 2013), implemented in GEMMA. Both models are expected to control for population structure and kinship. We used the centered genotype matrix (mean genotype = 0) for both models. Two datasets were tested, one set was the full 24 populations with 935 genotypes (six replicated samples were removed, related individuals were kept), and another was a reduced set, which excluded populations 10, 16, and 26 from NW Norway. To estimate a significant threshold, we ran a permutation test of the LMM where phenotypes were shuffled 1000 times for both datasets.

To estimate SNP effects, we used BSLMM, which estimates the genetic effect of each marker without assigning significance and is a type of modeling that has been used for genomic selection (Meuwissen et al., 2001). One of the advantages of the BSLMM is that, in addition to estimating the *PVE* (proportion of genetic effects contributing to the total phenotypic variance, “chip heritability”), we could also estimate the *GVE*_*β*_ (proportion of major genetic effects contributing to the genetic variation). To estimate how much the major SNP effects contribute to the total phenotypic variance (*PVE*_*β*_), we multiplied equations 13 (*PVE*) and 14 (*GVE*_*β*_) from Zhou et al. (2013). The BSLMM model was run with 10^5^ burn-in steps and 10^6^ to 10^7^ iterations for multiple runs to compare results, thinning sets to 10 and allowing for up to 500 sparse effects.

## Funding

Freezing tests were performed by Skogforsk and sponsored by NEFCO through the Programme for Environment and Climate Co-operation. This study was supported by grants from 10.13039/501100001862Formas, TC4F, Carl Tryggers Stiftelse, and Umeå Plant Science Center, Sweden.

## Author contributions

U.W., J.K., D.H., and X.R.W. designed the study; U.W. and J.K. conducted the freezing tests; J.O., D.H., W.Z., and X.R.W. performed the lab work; D.H., W.Z., and J.O. analyzed the data; D.H., J.O., and X.R.W. wrote the manuscript with contributions from all authors.

## Accession Numbers

Raw GBS library data reads have been deposited to NCBI under BioProject PRJNA687348 (https://www.ncbi.nlm.nih.gov/bioproject/PRJNA687348/). Final VCF files (https://doi.org/10.5878/8bg9-ah89 and https://doi.org/10.5878/rjzz-jk23), genotype population and phenotype information (https://doi.org/10.5878/0msc-3v36), population coordinates and environmental variables (https://doi.org/10.5878/45yn-ag55), and freeze test protocol and results (https://doi.org/10.5878/7pt2-ja36) are freely available from the Swedish National Data Service.

## Supplemental information

Document S1. Supplemental methods, Supplemental Figures 1–4, and Supplemental Tables 1–6

Document S2. Article plus Supplemental information

## Footnotes

Supplemental information is available at *Plant Communications Online*.
