# Supplementary material for: Divergent patterns between phenotypic and genetic variation in Scots pine
Source: Plant Commun. 2020 Dec 29;2(1):100139. doi: 10.1016/j.xplc.2020.100139 (PMC7816077; doi:10.1016/j.xplc.2020.100139)
Supplement: Document S1. Supplemental methods, Supplemental Figures 1–4, and Supplemental Tables 1–6 [file mmc1.pdf]

Plant Communications, Volume *2*

Supplemental Information

Divergent patterns between phenotypic and genetic variation in Scots pine

David Hall, Jenny Olsson, Wei Zhao, Johan Kroon, Ulfstand Wennström, and Xiao-Ru Wang

Divergent pattern between phenotypic and genetic variation in Scots pine

David Hall, Jenny Olsson, Wei Zhao, Johan Kroon, Ulfstand Wennström and Xiao-Ru Wang

Supporting information

Supplementary Methods

Sampling and freezing test

To get a comprehensive view of the hardiness variation and genetic diversity and possible genetic structure of the northwestern distribution range of Scots pine, we collected 54 populations ranging from Norway to western Russia, and covering latitudes 57.5°N - 69.1°N (Figure 1, Supplemental Table 1), of which 53 populations were freeze tested. For most stands, cones were collected by commercial forest companies in stands consisting of 100 – 1000 trees. Seeds were extracted from cones in bulk for each stand. Seeds were then randomly picked from these bulk collections to be used in the freezing test. However, cones from population 39 was collected around the forestry research station in Arkhangelsk, Russia and population 40 from a seed orchard based on local stand trees.

The 53 populations were randomly divided into five groups. Within a group, seeds were sown in seedling boxes, with seven by eleven pots. Based on earlier assessment of germination, one, two (most stands) or three seeds were sown per pot (see Table S1). Each population was randomly given a number 1-55 and divided in five groups (A-E) according to their number, 1-11, 12-22, etc. In replication one all populations were sown in numerical order from left to right. To avoid edge effects, populations within a box were moved one column to the right within box and replicate (population in column eleven was moved to column one). Each seedling's position in the freezer was mapped for calculation of edge effect (KantF). Seedlings were sown in unfertilized peat and grown in a dark greenhouse at +20°C for the first week. In the following weeks of 1-9, the seedlings had 20/4 hr light/dark condition at +20°C daytime and +15°C during night. From week 10 the nighttime was increased by 1 hr every week with +15°C daytime and +5°C during night to initiate bud dormancy. Before exposure to freezing temperatures the pots where controlled for number of seedlings. If more than one seed had germinated, only the most central seedling was kept. Seedling boxes were placed into a freezer chamber at ten different time points after the dormancy initiation treatment, for two hours at -10°C. First freezing was conducted at 39 days after dormancy initiation (DDI) and the final freezing at 70 DDI. The scoring of needle damage on each seedling after exposure to low temperatures was done at least 1 week after exposure to allow for discoloration to develop. Each seedling was only frozen once but each population was represented in each replication. The degree of needle damage was scored visually into seven classes: **0**, no needle discoloration; **1**, 1-20% of needles discolored; **2**, 20-40%; **3**, 40-60%; **4**, 60-80%; **5**, 80-99% and **6** when all needles are completely discolored. A low score indicates low needle damage and therefore higher tolerance to freezing temperature (Table S1). This testing protocol was established by the Forestry Research Institute of Sweden (Skogforsk) in Sävar (Andersson, 1992, Persson et al., 2010) as a standard method for monitoring the hardiness of Scots pine seed orchard crops for reforestation since the early 1980s. In total there were 18 replications, of which each population had up to 7 seedlings represented per replication, made over 10 freezing time points, of which 15 were visually scored by a single person. Needles were collected from seedlings for genotyping before they were subjected to freeze exposure.

Phenotypic analyses

Freezing damage was analyzed using a generalized linear mixed model (GLMM) with the 'glmer' function in the R-package 'lme4' (Bates et al., 2015). We assumed a Poisson

distributed response variable of damage categories as a function of the fixed effects of placement in the freezer (KantF), DDI, longitude and latitude at origin and their interactions. Individual plant was set as a random effect of the population within replication. Longitude, latitude and DDI was centered (subtracting the mean) and then scaled by dividing with their standard deviations, because these variables were on a different scale than the response variable and caused instability during model fitting. The average damage levels of the populations were calculated as the least square means (LSM) for each sampled population to remove some experimental effects. These LSMs were then used for regression analysis against latitude, longitude and growing degree days with a 5°C base (GDD5). We used linear regression with latitude and longitude while a logistic regression was used with GDD0 and GDD5 where the LSM values were first normalized to a range between 0 and 1. In addition, we performed simple kriging (i.e. we assume we know the mean of our sampled region) on the freezing damage LSM to extrapolate expected frost tolerance across the sampled space. The semivariogram was calculated and fitted to a Gaussian model in the R-package 'gstat' (Pebesma, 2004).

We also sampled the posterior distribution of freezing damage differentiation among populations to estimate Q_{ST} in this trait with a Markov chain Monte Carlo (MCMC) procedure. Q_{ST} is the quantitative trait equivalent to the fixation index (F_{ST}), which is a measure of population differentiation due to genetic structure, and the comparison between Q_{ST} and F_{ST} can thus indicate if a trait is under balancing selection, i.e. $Q_{ST} < F_{ST}$, or under divergent or local adaptation, i.e. $Q_{ST} > F_{ST}$ (Leinonen et al., 2013, Spitze, 1993, Whitlock, 1999). We utilized the MCMCglmm package in R (Hadfield, 2010) following the full model above but with population and individual plant as random effects. We used 100 000 iterations with a burnin of 50 000 and thinning of 10. Q_{ST} for freezing damage was calculated as follows:

$$Q_{ST} = \frac{\sigma_B^2}{\sigma_B^2 + 2h^2\sigma_W^2}$$

where σ_B^2 is the between population variance estimate and σ_W^2 is the within population variance estimate (Spitze, 1993, Whitlock, 1999). The σ_W^2 term is confounded and contains the total within population phenotypic variance. Due to lack of family structure across the samples it is difficult to estimate additive genetic variance, which in turn will result in an underestimated Q_{ST}-value. However, see the association mapping section under the genotype and environment association subheading for marker estimated heritability.

We estimated pairwise Q_{ST}-values and compare them to differences in environmental variables, latitude, longitude, and physical and environmental distance between populations. To calculate environmental distance we performed a principal component analysis of 68 environmental variables (Supplemental Table 2) and used the decomposition of variables to calculate the Euclidean distance $D_{i,j}$ between populations i and j in the multidimensional space:

$$D_{i,j} = \sqrt{\sum_{n=1}^{N} \left(PC_{n_j} - PC_{n_i}\right)^2}$$

Where N is the number of principal components that explain a majority of variance observed or where adding additional eigenvector provide little explanatory gain ("elbow" in the scree-plot). Pairwise Q_{ST} was calculated utilizing a generalized linear mixed model due to the categorical data structure of freeze damage assessment for each seedlot and replication.

GBS library preparations

We extracted DNA for seedlings from 23 populations (Supplemental Table 1) using EZNA SP Plant DNA Kit (Omega Bio-tek). GBS library was prepared using a *Pst*I high-fidelity restriction enzyme (New England Biolabs® Inc.), following the protocol of (Pan et al., 2015).

Briefly, 200 ng DNA from each seedling was individually digested and ligated to sequencing adapters (with individual barcode) simultaneously. This was carried out at 37 °C for 8 h followed by 65 °C for 30 min. Then, the digested and ligated DNA of 300 samples were pooled into each library, purified, and PCR-amplified. Fragment size of 350–450 bp was selected using an E-gel EX 2% agarose gel (Thermo Fisher Scientific) and purified. Paired-end sequencing (2 × 150 bp) was performed on Illumina HiSeq X Ten. In each library, we included a few samples as within and among library replicates.

Bioinformatics

Sequence read quality was assessed with FastQC (http://www.bioinformatics.babraham.ac.uk/projects/fastqc/). Adapter sequences and low quality bases (Phred quality <20) from the tail of each read were removed by using Trimmomatic (Bolger et al., 2014). Clean reads were cataloged by using the process_radtags module of Stacks v2.0 (Catchen et al., 2011) according to individual barcode. Reads shorter than 41 bases were discarded. Sequence reads were aligned to the *Pinus taeda* draft genome v1.01 (Neale et al., 2014, Zimin et al., 2014), by using the Burrows-Wheeler Aligner mem (BWA-MEM) algorithm with default parameters (Li, 2013). Variants were called using the SAMtools and BCFtools pipeline with default parameters (Catchen et al., 2013, Li, 2011). One previously genotyped population (Skillingaryd in Southern Sweden, population No. 8 with 48 individuals, Figure 1A) was included in this study, resulting in a total of 941 individuals from 24 populations in the final sequence dataset (Supplemental Table 4).

Several filtering steps were performed to minimize genotyping errors: SNPs located in repetitive regions (reference to *P. taeda* genome v1.01) and with mapping quality (MQ) <40, were removed; genotypes with genotype quality (GQ) <20 or read depth (DP) <5 were masked as missing; loci with a missing rate of >30%, minor allele frequency (MAF) <5%, heterozygosity >70% or not biallelic were also removed.

Genetic diversity and population structure

We first examined relatedness among individuals in each population following the procedure of (Hall et al., 2020) to remove highly related samples. The presence of related individuals, if undetected, would inflate population structure. Relatedness between samples was based on the estimator proposed by Ritland (1996), a Method-of-moments estimator (MME) present in the R-package ‘related’(Pew et al., 2015) which in turn is an R implementation of the software ‘COANCESTRY’ (Wang, 2011). It requires a large population allele frequency reference which is based on a representative set of individuals. We did not know the relatedness among samples a priori, but we assumed that related individuals would be among different populations and not affect the overall allele frequency estimates, and used all samples as the reference set. Related samples were removed iteratively. First we removed the sample that had the largest number of pairwise comparisons among samples with elevated relatedness, above cousin level 0.125. Then the next and so forth until no sample had an elevated relatedness to any other sample.

We estimated the nucleotide diversity at synonymous sites (π_4) and non-synonymous sites (π_0). Using *Pinus taeda* genome as reference for mapping, we could annotate the SNPs based on the General Feature Format (gff) annotation file of *P. taeda*. A position of a codon is said to be an n-fold degenerate site if only n of four possible nucleotides (A, C, G, and T) at this position specify the same amino acid. A nucleotide substitution at a fourfold degenerate site is referred to as a synonymous nucleotide substitution, and 0-fold degenerate site is nonsynonymous site. The observed (H_o) and expected (H_e) heterozygosity and fixation index F_{IS} in each populations and overall, and pairwise nucleotide diversity at all sites (π) and at 0-

fold (π_0) and 4-fold (π_4) degenerate sites were computed using VCFtools (https://vcftools.github.io/index.html).

To obtain an overview of the spatial pattern of diversity, we examined population structure using the R-implementation of TESS3 (Caye et al., 2016). TESS3 assumes that the genotypes are sampled from an admixed populations originating from K ancestral clusters, like other softwares, such as *fastStructure* (Raj et al., 2014), that estimates ancestral components. The likelihood method used then assumes that the probability of sampling from K number ancestral genotype pools is equal to the admixture coefficients. The probabilities are geographically constrained, meaning that neighboring samples are more likely to share ancestral genotypes. We ran the TESS3 algorithm with the assumptions of 1 to 5 ancestral populations (K) and each K was replicated 20 times. To avoid overestimating the number of potential clusters caused by the presence of isolation by distance (IBD), as is often found in continuous populations, we used *conStruct* v.1.03 (Bradburd et al., 2018) to identify structure in a spatially aware context. We tested both the spatial and non-spatial models with K values from 1 to 5 and 50,000 MCMC iterations for each test. Model cross-validation was run with 5000 iterations and 10 replicates to test which model best explains the distribution of genetic variation. For each best fit K, i.e. the number of ancestral layers that explains most of the variation, we conducted three independent runs to evaluate the convergence.

Genetic differentiation among populations was determined using pairwise F_{ST} (Weir and Cockerham, 1984) in Arlequin 3.5 (Excoffier and Lischer, 2010), where the statistical significance was assessed by 1023 permutations, at significance level of 0.05. To examine if the individual seedlings genotypes could be categorized into their respective populations and if the genetic variation can be attributed to geographic distance, we performed a principal component analysis (PCA) on the genetic covariances with EIGENSOFT v 6.1.4 (Patterson et al., 2006, Price et al., 2006).

Genotype and environment association

To examine possible allele frequency clines, as an initial screening of putative genotype-environment association, we calculated the association of allele frequencies in populations with latitude, longitude and GDD5. We also compared the observed allele correlations (Spearman's ρ) among the three variables to establish if there was an overlap between correlations.

To identify loci that are more or less differentiated than the average loci, we performed a F_{ST} outlier test of the SNPs using BayeScan (Foll and Gaggiotti, 2008). This method has been shown to have elevated type I error rates under IBD (de Villemereuil et al., 2014). However, we know from previous studies that the expected differentiation among loci is low (Hall et al., 2020:Tyrmi, 2020 #1139), and we are interested in examining putative patterns of all loci with elevated differentiation. We also used TESS3 (Caye et al., 2016) for F_{ST}-outlier loci detection, but in contrast to BayeScan, TESS3 is based on the estimated ancestral allele frequencies which in turn are geographically constrained. In addition, we further analyzed those highly differentiated loci identified by BayeScan using TESS3. We did this to see if they were a result of any shared ancestral component and if those components could be attributed to a specific geographic origin (Caye et al., 2016).

We considered whether environmental factors could have shaped the patterns of differentiation in all loci and among outliers by performing a redundancy analysis (RDA) over population allele frequencies and environmental parameters, a method that has been shown to be robust in comparative studies in detecting genotype by environment associations (Forester et al., 2018). We ran one full RDA based on two predictive matrices, environment and geographic distance in the form of Principal coordinates of neighbor matrices (PCNM). PCNMs considers the spatial input variables (longitude and latitude in this study) and determine the distance between sites and neighboring sites in particular. These distances are

then decomposed into a new set of independent spatial variables. The PCNM removes spatial autocorrelations (Dray et al., 2006). However, this requires assumptions about the connectedness between samplings sites which could inflate the explanatory power (Gilbert and Bennett, 2010). The two matrices are evaluated by separate RDAs on each matrix. One RDA with 68 environmental variable (Supplemental Table 2), and one with the 15 decomposed geographic distance variables. We applied a forward stepwise selection with the function *ordistep* in the vegan –R-package for parameters on the two models separately. The function employs permutation tests to only keep those that had the most influence. The algorithm perform a stepwise addition of variables that significantly contribute to the model fit. For each step the remaining variables are evaluated to find the variable that would explain most of the remaining variance and give the lowest AIC-value. This forward stepping procedure is performed until there is no more significant improvement of the model, making the best fit model with the fewest possible variables. We also made sure that none of the remaining variables from the forward selection shared high levels of information ($|R| < 0.75$) (Oksanen et al., 2019). The full model with the two matrices are then partitioned to evaluate the two predictors' relative importance and confounding using the function *varpart* in the 'vegan' R-package. We also ran a partial RDA, conditional on the geographic distance, to assess whether we could observe outlier SNPs exclusively shaped by the environment.

Evaluation of SNP-significance in the RDA was based on the number of significant constrained axis (K) evaluated with the *anova.cca* function in the 'vegan' R-package (Oksanen et al., 2019). We then performed significant tests following the method in (Capblancq et al., 2018). In short, for each loci the Mahalanobis distance is estimated within the variance covariance matrix of K number dimensions through the *covRob* function of the 'robust' R-package (Wang et al., 2020). Loci with an extreme Mahalanobis distance are considered significant. The Mahalanobis distances has a Chi-square distribution with K degrees of freedom and distances are considered significant at q-values ≤ 0.05 after adjusting p-values for a false discovery rate with the 'qvalue' R-package (Storey et al., 2020).

Association mapping with damage levels

To further examine genotype-phenotype and genotype-environment associations we applied more direct association mapping of genotypes to freeze damage levels and to population environmental variables. To accommodate a more uniform dataset in association mapping on the genotyped and phenotyped seedlings we had to normalize the data over replication and freezing time points. This was necessary because each seedling (genotype) was only phenotyped once and dormancy progression will strongly influence their resistance to frost. We used quantile normalization procedure from the Bioconductor R-package 'preprocessCore' (Bolstad, 2020), which results in comparable phenotypes across replicates. However, because the underlying data has a Poisson distribution the result from the quantile normalization is pseudo-continuous with a non-normal distribution.

Association mapping with damage levels were performed using both the univariate linear mixed model (LMM, Zhou and Stephens, 2012) and Bayesian sparse linear mixed model (BSLMM, Zhou et al., 2013) implemented in GEMMA. Both these models are expected to control for population structure and kinship. We used the centered genotype matrix (mean genotype = 0) for both models. Two datasets were used with both models. Data set one was the full dataset with 935 genotypes, (6 genotype replicated samples were removed) which included related individuals, and a reduced set which excluded populations 10, 16 and 26 of north western Norway. To estimate a significant threshold we ran a permutation test of the LMM where phenotypes were shuffled 1000 times for both data sets. Minimum p-values for each permutation was stored and the lower 5% tail of the distribution considered a significant

threshold comparable to a 5% FDR. We also considered the lowest observed *p*-value of the permutation runs as a threshold for a highly significant effect.

To estimate SNP effects we used BSLMM, which estimates the genetic effect of each marker without assigning significance, and is a type of modelling that has been used for genomic selection (Meuwissen et al., 2001). One of the advantages of the BSLMM is that it is a mixed distribution model where in addition to estimate the *PVE* (proportion of genetic effects contributing the total phenotypic variance, "chip heritability"), we can also get an estimate of the GVE_β (proportion of major genetic effects contributing to the genetic variation) almost independently. To get an estimate of how much major SNP effects contributes to the total phenotypic variance, GVE_β, we multiplied equations 13 (*PVE*) and 14 (GVE_β) from Zhou et al. (2013) and thus get the following:

$$PVE_\beta(\tilde{\beta}, \boldsymbol{u}, \tau) = \frac{V(\boldsymbol{X}\tilde{\beta})}{V(\boldsymbol{X}\tilde{\beta} + \boldsymbol{u}) + \tau^{-1}}$$

where $\boldsymbol{u}$ is the polygenic component which captures the combined small effects of all markers, τ the error variance, $\tilde{\beta}$ the major SNP effects or "sparse effects" and $\boldsymbol{X}$ is the genotype matrix.

References

Andersson, B. (1992). Autumn frost hardiness of *Pinus sylvestris* offspring from seed orchard grafts of different ages. Scand J Forest Res **7**:367-375.

Bates, D., Machler, M., Bolker, B.M., and Walker, S.C. (2015). Fitting linear mixed-effects models using lme4. J Stat Softw **67**:1-48.

Bolger, A.M., Lohse, M., and Usadel, B. (2014). Trimmomatic: a flexible trimmer for Illumina sequence data. Bioinformatics **30**:2114-2120.

Bolstad, B. (2020). preprocessCore: A collection of pre-processing functions. *R package version 1.50.0*. https://github.com/bmbolstad/preprocessCore.

Bradburd, G.S., Coop, G.M., and Ralph, P.L. (2018). Inferring continuous and discrete population genetic structure across space. Genetics **210**:33-52.

Capblancq, T., Luu, K., Blum, M.G.B., and Bazin, E. (2018). Evaluation of redundancy analysis to identify signatures of local adaptation. Mol Ecol Resour **18**:1223-1233.

Catchen, J., Hohenlohe, P.A., Bassham, S., Amores, A., and Cresko, W.A. (2013). Stacks: an analysis tool set for population genomics. Mol Ecol **22**:3124-3140.

Catchen, J.M., Amores, A., Hohenlohe, P., Cresko, W., and Postlethwait, J.H. (2011). Stacks: Building and genotyping loci de novo from short-read sequences. G3 **1**:171-182.

Caye, K., Deist, T.M., Martins, H., Michel, O., and François, O. (2016). TESS3: fast inference of spatial population structure and genome scans for selection. Mol Ecol Resour **16**:540-548.

de Villemereuil, P., Frichot, É., Bazin, É., François, O., and Gaggiotti, O.E. (2014). Genome scan methods against more complex models: when and how much should we trust them? Mol Ecol **23**:2006-2019.

Dray, S., Legendre, P., and Peres-Neto, P.R. (2006). Spatial modelling: a comprehensive framework for principal coordinate analysis of neighbour matrices (PCNM). Ecological Modelling **196**:483-493.

Excoffier, L., and Lischer, H.E.L. (2010). Arlequin suite ver 3.5: a new series of programs to perform population genetics analyses under Linux and Windows. Mol Ecol Resour **10**:564-567.

Foll, M., and Gaggiotti, O. (2008). A genome-scan method to identify selected loci appropriate for both dominant and codominant markers: a Bayesian perspective. Genetics **180**:977-993.

Forester, B.R., Lasky, J.R., Wagner, H.H., and Urban, D.L. (2018). Comparing methods for detecting multilocus adaptation with multivariate genotype–environment associations. Mol Ecol **27**:2215-2233.

Gilbert, B., and Bennett, J.R. (2010). Partitioning variation in ecological communities: do the numbers add up? Journal of Applied Ecology **47**:1071-1082.

Hadfield, J.D. (2010). MCMC methods for multi-response generalized linear mixed models: The MCMCglmm R package. J Stat Softw **33**:1-22.

Hall, D., Zhao, W., Wennström, U., Andersson Gull, B., and Wang, X.-R. (2020). Parentage and relatedness reconstruction in *Pinus sylvestris* using genotyping-by-sequencing. Heredity:DOI: https://doi.org/10.1038/s41437-41020-40302-41433.

Leinonen, T., McCairns, R.J.S., O'Hara, R.B., and Merila, J. (2013). Q_{ST}-F_{ST} comparisons: evolutionary and ecological insights from genomic heterogeneity. Nat Rev Genet **14**:179-190.

Li, H. (2011). A statistical framework for SNP calling, mutation discovery, association mapping and population genetical parameter estimation from sequencing data. Bioinformatics **27**:2987-2993.

Li, H. (2013). Aligning sequence reads, clone sequences and assembly contigs with BWA-MEM. arXiv preprint arXiv:1303.3997.

Meuwissen, T.H.E., Hayes, B.J., and Goddard, M.E. (2001). Prediction of total genetic value using genome-wide dense marker maps. Genetics **157**:1819-1829.

Neale, D.B., Wegrzyn, J.L., Stevens, K.A., Zimin, A.V., Puiu, D., Crepeau, M.W., Cardeno, C., Koriabine, M., Holtz-Morris, A.E., Liechty, J.D., et al. (2014). Decoding the massive genome of loblolly pine using haploid DNA and novel assembly strategies. Genome Biol **15**:R59.

Oksanen, J., Blanchet, F.G., Friendly, M., Kindt, R., Legendre, P., McGlinn, D., Minchin, P.R., O'Hara, R.B., Simpson, G.L., Solymos, P., et al. (2019). vegan: Community ecology package. *R package version 2.5-6.* https://CRAN.R-project.org/package=vegan.

Pan, J., Wang, B.S., Pei, Z.Y., Zhao, W., Gao, J., Mao, J.F., and Wang, X.R. (2015). Optimization of the genotyping-by-sequencing strategy for population genomic analysis in conifers. Mol Ecol Resour **15**:711-722.

Patterson, N., Price, A.L., and Reich, D. (2006). Population structure and eigenanalysis. Plos Genet **2**.

Pebesma, E.J. (2004). Multivariable geostatistics in S: the gstat package. Computers & Geosciences **30**:683-691.

Persson, T., Andersson, B., and Ericsson, T. (2010). Relationship between autumn cold hardiness and field performance in northern *Pinus sylvestris*. Silva Fennica **44**:255-266.

Pew, J., Muir, P.H., Wang, J.L., and Frasier, T.R. (2015). related: an R package for analysing pairwise relatedness from codominant molecular markers. Mol Ecol Resour **15**:557-561.

Price, A.L., Patterson, N.J., Plenge, R.M., Weinblatt, M.E., Shadick, N.A., and Reich, D. (2006). Principal components analysis corrects for stratification in genome-wide association studies. Nat Genet **38**:904-909.

Raj, A., Stephens, M., and Pritchard, J.K. (2014). fastSTRUCTURE: Variational inference of population structure in large SNP data sets. Genetics **197**:573-589.

Ritland, K. (1996). Estimators for pairwise relatedness and individual inbreeding coefficients. Genetical Research **67**:175-185.

Spitze, K. (1993). Population structure in *Daphnia obtusa*: quantitative genetic and allozymic variation. Genetics **135**:367-374.

Storey, J.D., Bass, A.J., Dabney, A., Robinson, D., and Warnes, G. (2020). qvalue: Q-value estimation for false discovery rate control. *R package version 2.20.0*. http://github.com/jdstorey/qvalue.

Wang, J. (2011). COANCESTRY: a program for simulating, estimating and analysing relatedness and inbreeding coefficients. Mol Ecol Resour **11**:141-145.

Wang, J., Zamar, R., Marazzi, A., Yohai, V., Salibian-Barrera, M., Maronna, R., Zivot, E., Rocke, D., Martin, D., Maechler, M., et al. (2020). robust: Robust Library. *R Package Version 0.5-00.* https://CRAN.R-project.org/package=robust.

Weir, B.S., and Cockerham, C.C. (1984). Estimating F-statistics for the analysis of population structure. Evolution **38**:1358-1370.

Whitlock, M.C. (1999). Neutral additive genetic variance in a metapopulation. Gen Res **74**:215-221.

Zhou, X., Carbonetto, P., and Stephens, M. (2013). Polygenic modeling with Bayesian sparse linear mixed models. Plos Genet **9**:e1003264.

Zhou, X., and Stephens, M. (2012). Genome-wide efficient mixed-model analysis for association studies. Nat Genet **44**:821-824.

Zhou, X., and Stephens, M. (2014). Efficient multivariate linear mixed model algorithms for genome-wide association studies. Nature Methods **11**:407-409.

Zimin, A., Stevens, K.A., Crepeau, M., Holtz-Morris, A., Koriabine, M., Marçais, G., Puiu, D., Roberts, M., Wegrzyn, J.L., de Jong, P.J., et al. (2014). Sequencing and assembly of the 22-Gb Loblolly pine genome. Genetics **196**:875-890.

Tables

Table S1. Population information collection year and seeds sown per pot. Hardiness determined by average damage on needles after freeze test. LSM: the model corrected hardiness. NP: No. seedlings subjected to freezing test. NG: No. seedlings genotyped.

Population ID	Name	Collected	Seeds/ pot	Country	Lat.	Long.	GDD5	LSM (Damage)	NP	NG
1	Molde, Gjemnes, Skodje	1998	2	Norway	62.80	7.50	10521	4.03	104	59
2	Vågå, Oppland	2006	2	Norway	61.86	9.05	8243	3.14	102	21
3	Hemne, Sør-Trøndelag	1999	2	Norway	63.30	9.12	10215	3.34	103	66
4	Ringerike, Buskerud	2004	2	Norway	60.28	9.95	12483	3.93	104	61
5	Åsnes, Hedmark	2002	2	Norway	60.60	12.08	12204	4.18	105	-
6	Trysil, Hedmark	2005	2	Norway	61.22	12.37	8490	3.29	101	-
7	Torsby 1	2016	2	Sweden	60.20	12.90	12879	3.87	105	-
8	Skillingaryd	1999	1	Sweden	57.42	14.02	12906	-	-	48
9	Hattkullen B	1995	3	Sweden	60.89	14.23	8910	3.27	105	-
10	Beiarn, Nordland	1988	3	Norway	67.00	14.24	8243	2.22	103	60
11	Laforsen-Kårböle	1998	2	Sweden	61.97	15.48	9053	3.06	103	-
12	Gunnilbo_Kulheden	1995	3	Sweden	59.90	15.80	12987	3.86	90	59
13	Hammarstrand	1995	3	Sweden	63.15	16.20	8850	3.41	102	20
14	Sollefteå kommun	1998	2	Sweden	63.17	17.01	8925	3.15	105	-
15	Skorped	2016	2	Sweden	63.40	17.90	8535	2.70	105	-
16	Kirkesmoen, Troms	2011	2	Norway	68.90	18.30	4476	0.72	104	65
17	Abborrträskliden	1998	2	Sweden	64.82	18.80	8168	2.13	99	-
18	Arvidsjaur	1998	2	Sweden	65.60	19.20	7403	1.81	97	-
19	Kullsjöliden	1992	2	Sweden	64.10	19.80	8423	1.98	94	-
20	Nyträsk	1998	2	Sweden	65.00	20.50	8085	2.13	105	-
21	Harads	1998	2	Sweden	66.20	21.00	7860	1.87	102	-
22	Uusikaupunki	2004	2	Finland	60.84	21.49	12663	3.96	60	50
23	Tärendö	1998	2	Sweden	67.10	22.70	5370	0.78	104	-
24	Kuttainen	2003	2	Sweden	68.20	22.80	4650	0.66	93	-
25	Almajärvi	1998	3	Sweden	68.00	23.20	4854	0.50	102	-
26	Alta, Stengelsen	2013	2	Norway	69.87	23.27	4704	0.78	105	60
27	Kaunisvaara	1998	3	Sweden	67.50	23.30	5208	0.72	105	-
28	Svanstein	2016	2	Sweden	66.60	23.80	7733	1.00	84	-
29	Kourevesi	1977	1	Finland	62.00	24.80	9165	3.63	78	-
30	Rovaniemen mlk	2002	2	Finland	66.90	25.40	7718	1.09	105	20
31	Äänekoski	1999	2	Finland	62.80	25.70	8798	2.79	99	20
33	Riistina	1985	1	Finland	61.50	27.40	9998	3.81	25	17
34	Pudasjärvi	2004	2	Finland	65.50	27.60	7905	1.41	102	19
35	Suomussalmi	2002	2	Finland	65.00	29.10	7988	1.20	104	-
36	Kerimäki	2007	2	Finland	61.90	29.40	9548	3.43	105	-
37	Kuhmo	1997	2	Finland	64.10	29.60	8430	2.38	104	66
38	Archangelsk stand 5	2015	2	Russia	62.10	40.60	9075	3.15	100	18
39	Archangelsk stand 13	2016	2	Russia	64.52	40.70	8333	2.44	70	57
40	Archangelsk seed orchard	2015	2	Russia	61.00	42.30	9285	3.23	103	24
41	Archangelsk stand 12	2016	2	Russia	62.00	44.70	8963	3.37	87	-
43	Archangelsk stand 10	2016	2	Russia	61.90	45.00	9060	2.84	98	-
44	Archangelsk stand 11	2016	2	Russia	61.90	45.00	9060	2.89	95	-
45	Archangelsk stand 8	2016	2	Russia	61.90	45.00	9060	2.69	105	-
46	Archangelsk stand 7	2016	2	Russia	61.70	45.50	9263	3.47	102	-
47	Archangelsk stand 3	2015	2	Russia	61.20	46.20	9135	3.07	103	20
48	Archangelsk stand 4	2015	2	Russia	61.20	46.20	9135	2.68	105	-
49	Archangelsk stand 6	2016	2	Russia	62.10	46.60	8603	3.10	104	-
50	Archangelsk stand 2	2014	2	Russia	61.20	48.50	9233	3.03	103	-
51	Udora, Komi	2000	2	Russia	64.30	49.20	7935	1.40	79	19
52	Megdurechensk, Komi	2000	2	Russia	63.10	50.80	8640	2.03	83	19
53	Mechura, Komi	2000	2	Russia	63.80	51.20	8205	2.20	95	-
54	Ust-Kulom, Komi	2000	2	Russia	61.50	54.00	9330	2.78	97	-
55	Pomozdino, Komi	2000	2	Russia	62.10	54.30	8933	2.54	78	64
56	Sosnogorsk, Komi	2000	2	Russia	63.68	54.76	8003	1.82	8	9
**Total**									5028	941

Table S2. The 68 climatic variables used for phenotype- and genotype-environment association analyses. Bold text indicates those kept by redundancy analysis (RDA).

Variable	Information	Reference
BIO1	Annual Mean Temperature	1
BIO2	Mean Diurnal Range (Mean of monthly (max temp - min temp))	1
BIO3	Isothermality (BIO2/BIO7) (×100)	1
BIO4	Temperature Seasonality (standard deviation ×100)	1
BIO5	Max Temperature of Warmest Month	1
BIO6	Min Temperature of Coldest Month	1
BIO7	Temperature Annual Range (BIO5-BIO6)	1
**BIO8**	**Mean Temperature of Wettest Quarter**	**1**
BIO9	Mean Temperature of Driest Quarter	1
BIO10	Mean Temperature of Warmest Quarter	1
BIO11	Mean Temperature of Coldest Quarter	1
BIO12	Annual Precipitation	1
BIO13	Precipitation of Wettest Month	1
BIO14	Precipitation of Driest Month	1
BIO15	Precipitation Seasonality (Coefficient of Variation)	1
BIO16	Precipitation of Wettest Quarter	1
BIO17	Precipitation of Driest Quarter	1
BIO18	Precipitation of Warmest Quarter	1
BIO19	Precipitation of Coldest Quarter	1
frs01	Frost day frequency in January	2
frs02	Frost day frequency in February	2
frs03	Frost day frequency in March	2
frs04	Frost day frequency in April	2
frs05	Frost day frequency in May	2
frs06	Frost day frequency in June	2
frs07	Frost day frequency in July	2
frs08	Frost day frequency in August	2
frs09	Frost day frequency in September	2
frs10	Frost day frequency in October	2
frs11	Frost day frequency in November	2
frs12	Frost day frequency in December	2
vap01	Vapor pressure in January	2
vap02	Vapor pressure in February	2
vap03	Vapor pressure in March	2
vap04	Vapor pressure in April	2
vap05	Vapor pressure in May	2
vap06	Vapor pressure in June	2
vap07	Vapor pressure in July	2
vap08	Vapor pressure in August	2
vap09	Vapor pressure in September	2

Table S2 contd.

vap10	Vapor pressure in October	2
vap11	Vapor pressure in November	2
vap12	Vapor pressure in December	2
wet01	Wet day frequency in January	2
wet02	Wet day frequency in February	2
wet03	Wet day frequency in March	2
wet04	Wet day frequency in April	2
wet05	Wet day frequency in May	2
wet06	Wet day frequency in June	2
wet07	Wet day frequency in July	2
wet08	Wet day frequency in August	2
wet09	Wet day frequency in September	2
wet10	Wet day frequency in October	2
**wet11**	**Wet day frequency in November**	**2**
wet12	Wet day frequency in December	2
embergerQ	Emberger's pluviothermic quotient: a metric that was designed to differentiate among Mediterranean type climates	3
**GDD0**	**Sum of mean monthly temperature for months with mean temperature greater than 0°C multiplied by number of days**	**3**
GDD5	Sum of mean monthly temperature for months with mean temperature greater than 5°C multiplied by number of days	3
**maxTempColdestMonth**	**Max. temp. of the coldest month**	**3**
sc	Soil organic carbon	4
SpH	Soil pH	4
gdd	Annual GDD with 5°C baseline	4
uvb1	Annual Mean UV-B	5
uvb2	Annual UV-B Seasonality (standard deviation)	5
uvb3	Mean UV-B of Highest Month	5
uvb4	Mean UV-B of Lowest Month	5
uvb5	Sum of Monthly Mean UV-B during Highest Quarter	5
uvb6	Sum of Monthly Mean UV-B during Lowest Quarter	5

References

1. Fick, S.E., and Hijmans, R.J. (2017). WorldClim 2: new 1-km spatial resolution climate surfaces for global land areas. International Journal of Climatology 37:4302-4315.
2. Mitchell, T.D., and Jones, P.D. (2005). An improved method of constructing a database of monthly climate observations and associated high-resolution grids. International Journal of Climatology 25:693-712.
3. Title, P.O., and Bemmels, J.B. (2018). ENVIREM: an expanded set of bioclimatic and topographic variables increases flexibility and improves performance of ecological niche modeling. Ecography 41:291-307.
4. SAGE. Atlas of the Biosphere--The Center for Sustainability and the Global Environment, N.I.F.E.S., University of Wisconsin-Madison.
5. Beckmann, M., Václavík, T., Manceur, A.M., Šprtová, L., von Wehrden, H., Welk, E., and Cord, A.F. (2014). glUV: a global UV-B radiation data set for macroecological studies. Methods in Ecology and Evolution 5:372-383.

Table S3. Summary of GBS results for the analyzed Scots pine seedlings. Numbers in parentheses are the ±1 standard error. Reads with a low coverage or depth (less than or equal to 5x) were discarded.

	**Mean**	**Median**
Reads per individual	2,355,433 (± 94,478)	1,630,707
Coverage (Mbp, >=5x)	2.53 (± 0.026)	2.45
Depth (>=5x)	87x (± 1.51)	32x
Mapping rate	96.36 % (± 0.153)	97.41 %

Table S4. Number of related (including replicates) and unrelated individuals in each population. Only unrelated individuals were used in the genetic analyses.

**Population ID**	**Country**	**Related**	**Unrelated**	**Total**
1	Norway	21	38	59
2	Norway	3	18	21
3	Norway	26	40	66
4	Norway	27	34	61
8	Sweden	2	46	48
10	Norway	11	49	60
12	Sweden	3	56	59
13	Sweden	1	19	20
16	Norway	3	62	65
22	Finland	17	33	50
26	Norway	1	59	60
30	Finland	0	20	20
31	Finland	4	16	20
33	Finland	7	10	17
34	Finland	0	19	19
37	Finland	2	64	66
38	Russia	3	15	18
39	Russia	45	12	57
40	Russia	6	18	24
47	Russia	0	20	20
51	Russia	0	19	19
52	Russia	1	18	19
55	Russia	11	53	64
56	Russia	1	8	9
**Total**		**195**	**746**	**941**

Table S5. Pairwise F_{ST} estimates between all genotyped populations. Darker shade of red indicate higher pairwise differentiation.

Pop	1	2	3	4	8	10	12	13	16	22	26	30	31	33	34	37	38	39	40	47	51	52	55
**56**	0.0190	0.0136	0.0192	0.0129	0.0135	0.0191	0.0161	0.0152	0.0149	0.0058	0.0087	0.0122	0.0117	0.0126	0.0102	0.0134	0.0018	0.0057	0.0053	0.0022	0.0036	0.0041	0.0027
**55**	0.0158	0.0076	0.0139	0.0082	0.0116	0.0145	0.0101	0.0038	0.0084	0.0068	-0.0021	0.0073	0.0077	0.0108	0.0075	0.0053	-0.0036	-0.0031	0.0028	-0.0009	-0.0003	0.0003	
**52**	0.0144	0.0141	0.0143	0.0072	0.0091	0.0140	0.0105	0.0125	0.0120	0.0042	0.0053	0.0093	0.0096	0.0094	0.0049	0.0070	0.0013	0.0022	0.0000	-0.0004	0.0001		
**51**	0.0132	0.0152	0.0123	0.0050	0.0076	0.0124	0.0105	0.0123	0.0114	0.0042	0.0059	0.0063	0.0112	0.0097	0.0025	0.0054	0.0030	0.0032	-0.0004	-0.0025			
**47**	0.0152	0.0072	0.0153	0.0110	0.0119	0.0155	0.0105	0.0035	0.0082	-0.0011	-0.0024	0.0076	0.0051	0.0067	0.0071	0.0062	-0.0087	-0.0060	-0.0009				
**40**	0.0098	0.0024	0.0085	0.0020	0.0049	0.0102	0.0040	-0.0031	0.0034	0.0022	-0.0072	0.0028	0.0019	0.0050	0.0022	0.0006	-0.0079	-0.0072					
**39**	0.0020	0.0130	-0.0002	-0.0083	-0.0041	-0.0012	-0.0011	0.0119	0.0075	-0.0028	0.0066	-0.0022	0.0032	0.0014	-0.0084	-0.0034	0.0072						
**38**	0.0050	0.0137	0.0028	-0.0065	-0.0033	0.0049	0.0023	0.0110	0.0105	-0.0030	0.0074	0.0007	0.0063	0.0014	-0.0034	-0.0007							
**37**	0.0091	0.0045	0.0087	0.0053	0.0069	0.0089	0.0056	0.0031	0.0048	-0.0042	-0.0034	0.0029	0.0031	0.0022	0.0013								
**34**	0.0099	0.0015	0.0087	0.0069	0.0081	0.0075	0.0066	-0.0023	0.0022	-0.0040	-0.0089	0.0020	-0.0019	0.0026									
**33**	0.0076	0.0032	0.0061	-0.0016	0.0015	0.0072	0.0033	-0.0010	0.0063	0.0016	-0.0051	0.0027	0.0027										
**31**	0.0073	0.0093	0.0043	-0.0007	0.0019	0.0054	0.0039	0.0070	0.0071	-0.0030	0.0016	0.0040											
**30**	0.0105	0.0037	0.0097	0.0059	0.0068	0.0085	0.0063	-0.0006	0.0046	-0.0029	-0.0054												
**26**	-0.0045	0.0053	-0.0066	-0.0122	-0.0076	-0.0083	-0.0031	0.0060	0.0000	-0.0147													
**22**	0.0018	-0.0028	-0.0028	-0.0103	-0.0051	-0.0007	-0.0044	-0.0088	-0.0027														
**16**	0.0055	0.0071	0.0031	-0.0011	0.0026	-0.0001	0.0051	0.0071															
**13**	-0.0037	0.0061	-0.0049	-0.0113	-0.0066	-0.0045	-0.0045																
**12**	0.0053	0.0000	0.0045	0.0006	0.0019	0.0056																	
**10**	0.0071	0.0001	0.0059	0.0054	0.0074																		
**8**	0.0048	-0.0033	0.0058	0.0020																			
**4**	0.0005	-0.0110	0.0030																				
**3**	0.0027	-0.0025																					
**2**	-0.0005																						

Table S6. Posterior distribution of the hyper parameters Bayesian sparse linear mixed models. The 95 % HPD regions of the hyper-parameters posterior distributions and the point estimate of the highest posterior density (HPD, from Fig. 4F and G of the main text). *PVE* is the proportion of phenotypic variance explained by all markers, GVE_β the proportion of PVE explained by the large, or sparse, effects and PVE_β the proportion of the phenotypic variation explained by large effect alleles. γ_N is the posterior distribution of the number of large effect. Point is the point along the x-axis with the highest density in y.

Full population set	2.50%	50%	97.50%	Point
PVE	0.478	0.580	0.681	0.584
GVE_β	0.147	0.357	0.663	0.328
PVE_β	0.087	0.206	0.373	0.190
γ_N	3	39	285	10
Reduced population set*				
PVE	0.427	0.557	0.690	0.555
GVE_β	0	0.316	0.898	0.034
PVE_β	0	0.100	0.807	0.010
γ_N	0	67	453	1

* Reduced population set is without population (10, 16, and 26) from the north western part of the distribution.

Figures

Figure S1

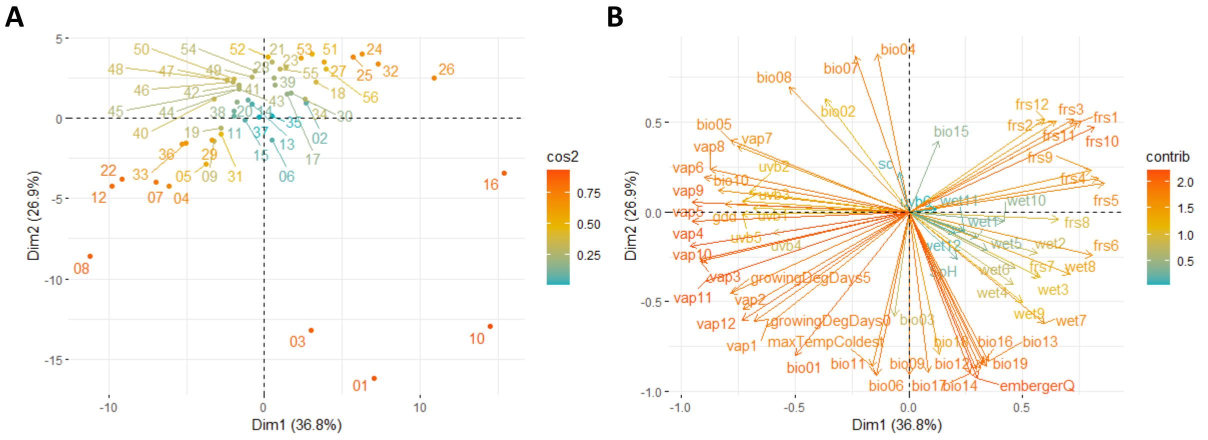

Figure S1. PCA of the environmental variables. (A) The populations' locations in the PCA-space. **(B)** The contributing variables and their direction in the first two PC-axis. Colors towards dark imply greater contribution.

Figure S2

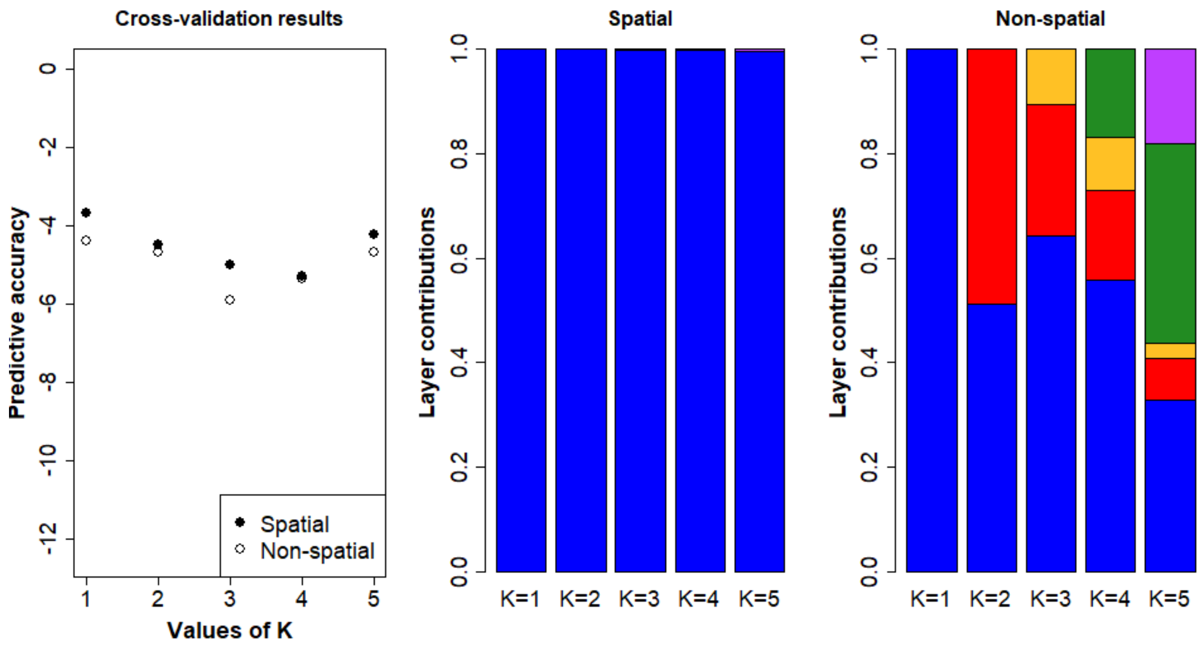

Figure S2. Cross-validation results and ancestral layer contributions to the total covariance with *K*=1 to *K*=5 for both the spatial and non-spatial model.

Figure S3

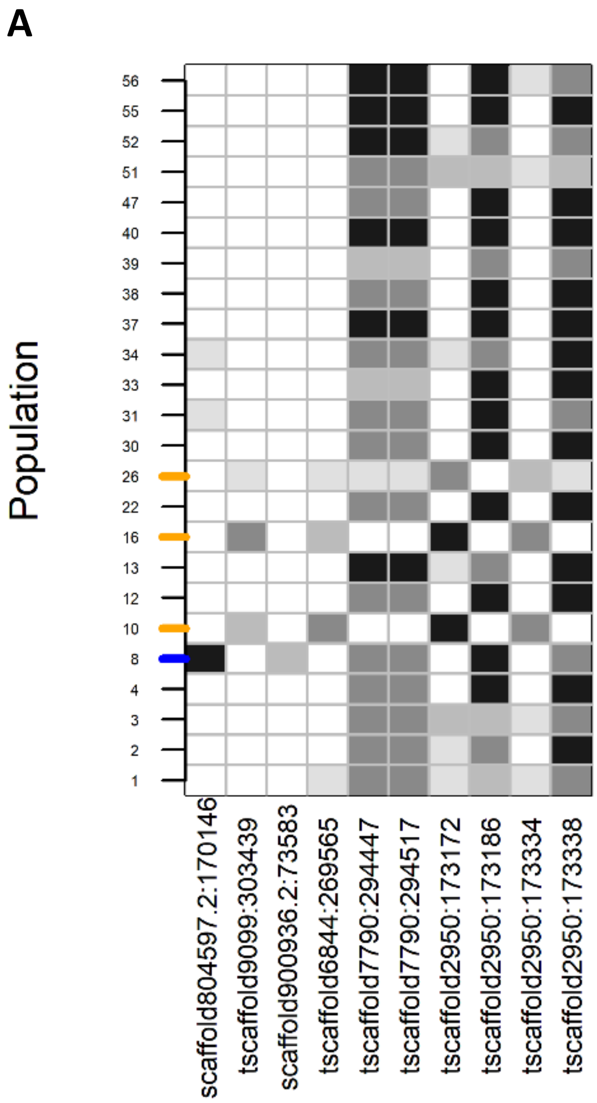

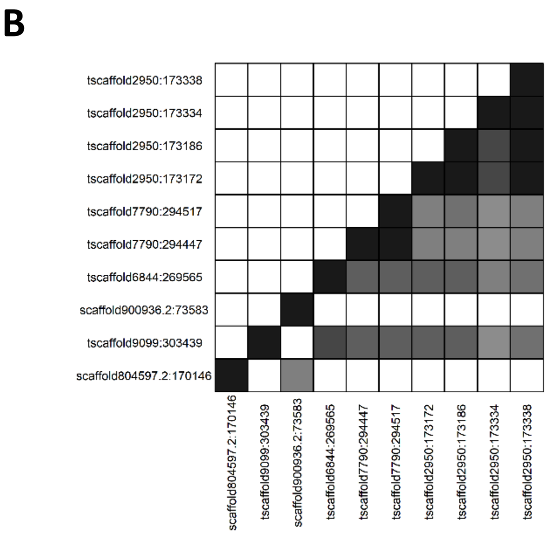

Figure S3. Population level linkage structure among ten of the most differentiated loci. (**A**) Orange tick marks indicate populations in the northwestern cluster and blue the southern cluster. Darker color indicate higher allele frequency or LD. Population ID on the y-axis and SNP on the x-axis. **(B)** LD (r^2) between the 10 loci

Figure S4

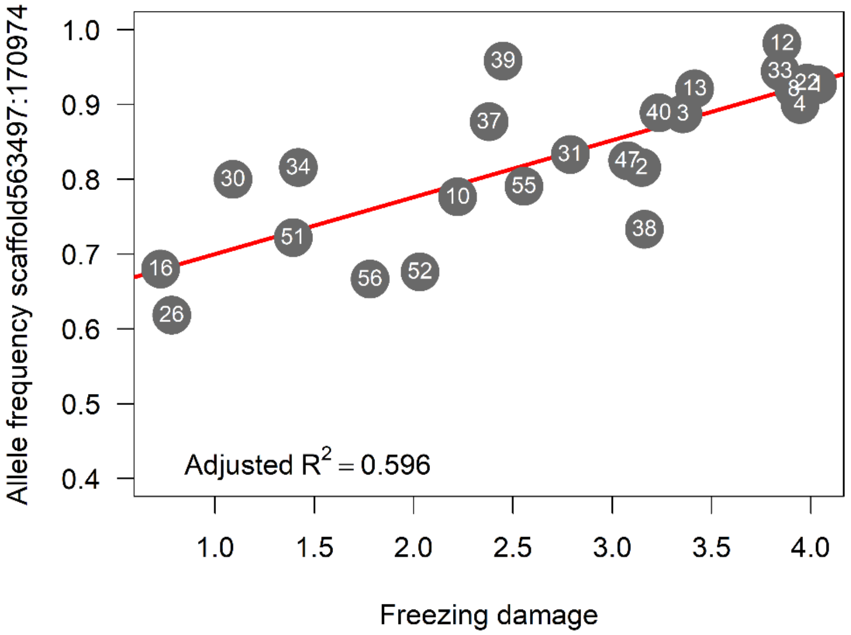

Figure S4. The allele frequencies of SNP scaffold563497:170974 as a function of freeze damage among populations, displayed as numbers within the points. Scaffold563497:170974 is the SNP that correlates most with freeze damage and is also highly correlated with the environmental variable vap2 but less with latitude or longitude see Figure 3D of the main text.
