# Supplementary material for: Divergent patterns between phenotypic and genetic variation in Scots pine
Source: Plant Commun. 2020 Dec 29;2(1):100139. doi: 10.1016/j.xplc.2020.100139 (PMC7816077; doi:10.1016/j.xplc.2020.100139)
Supplement: Document S2. Article plus Supplemental information [file mmc2.pdf]

Plant Communications
Research Article

CellPress
Partner Journal

Divergent patterns between phenotypic and genetic variation in Scots pine

David Hall[1], Jenny Olsson[1], Wei Zhao[1,2], Johan Kroon[3], Ulfstand Wennström[3] and Xiao-Ru Wang[1,2,*]

[1]Department of Ecology and Environmental Science, Umeå Plant Science Center, Umeå University, Umeå, Sweden

[2]Advanced Innovation Center for Tree Breeding by Molecular Design, College of Biological Sciences and Technology, Beijing Forestry University, Beijing, China

[3]The Forestry Research Institute of Sweden (Skogforsk), Uppsala Sweden

***Correspondence: Xiao-Ru Wang (xiao-ru.wang@umu.se)**

https://doi.org/10.1016/j.xplc.2020.100139

ABSTRACT

In boreal forests, autumn frost tolerance in seedlings is a critical fitness component because it determines survival rates during regeneration. To understand the forces that drive local adaptation in this trait, we conducted freezing tests in a common garden setting for 54 *Pinus sylvestris* (Scots pine) populations (>5000 seedlings) collected across Scandinavia into western Russia, and genotyped 24 of these populations (>900 seedlings) at >10 000 SNPs. Variation in cold hardiness among populations, as measured by Q_{ST}, was above 80% and followed a distinct cline along latitude and longitude, demonstrating significant adaptation to climate at origin. In contrast, the genetic differentiation was very weak (mean F_{ST} 0.37%). Despite even allele frequency distribution in the vast majority of SNPs among all populations, a few rare alleles appeared at very high or at fixation in marginal populations restricted to northwestern Fennoscandia. Genotype–environment associations showed that climate variables explained 2.9% of the genetic differentiation, while genotype–phenotype associations revealed a high marker-estimated heritability of frost hardiness of 0.56, but identified no major loci. Very extensive gene flow, strong local adaptation, and signals of complex demographic history across markers are interesting topics of forthcoming studies on this species to better clarify signatures of selection and demography.

Keywords: clinal variation, cold hardiness, genetic diversity, population structure, *Pinus sylvestris*

Hall D., Olsson J., Zhao W., Kroon J., Wennström U., and Wang X.-R. (2021). Divergent patterns between phenotypic and genetic variation in Scots pine. Plant Comm. **2**, 100139.

INTRODUCTION

Most boreal forest species have wide distribution ranges within which ecotype-specific variation and adaptive clines potentially arise across heterogeneous environments. Clinal trait variation is a response to spatially varying selection along environmental gradients. Strong local adaptations have been observed among tree populations in a plethora of published common garden experiments (e.g., Rehfeldt, 1989; Shutyaev and Giertych, 1998; Alberto et al., 2013), which identify a wide array of traits that affect fitness. For local adaptation to occur in species with large distribution ranges and extensive gene flow, such as Scots pine (*Pinus sylvestris* L.), selection has to be strong enough to overcome the homogenizing force of gene flow (Savolainen et al., 2007).

Selection on trait variation along environmental gradients is expected to generate allele frequency clines at loci that control a trait. However, distinguishing the impacts of local adaptation on allele frequency variation from those imposed by neutral evolutionary forces is often a challenging task in boreal settings and particularly for conifer trees (Zhao et al., 2020). Neutral forces have certainly acted on boreal conifer species when they tracked the receding ice sheets after the Last Glacial Maximum (LGM) and recolonized northern regions from their southern ice age refugia. Such migration routes expose migrating individuals to environmental clines that change with latitude. This confounds isolation by distance (IBD) (Wright, 1943) with isolation by environment and colonization (Wang and Bradburd, 2014), making inferences about the impacts of neutral and selective forces difficult to draw. In addition, selection on standing variation in quantitative polygenic traits may require only minute allele frequency shifts to facilitate local adaptation (Csilléry et al., 2018). This means that even if selection on a trait is strong, the identification of adaptive changes in minor allele

Published by the Plant Communications Shanghai Editorial Office in association with Cell Press, an imprint of Elsevier Inc., on behalf of CSPB and CEMPS, CAS.

This is an open access article under the CC BY license (http://creativecommons.org/licenses/by/4.0/).

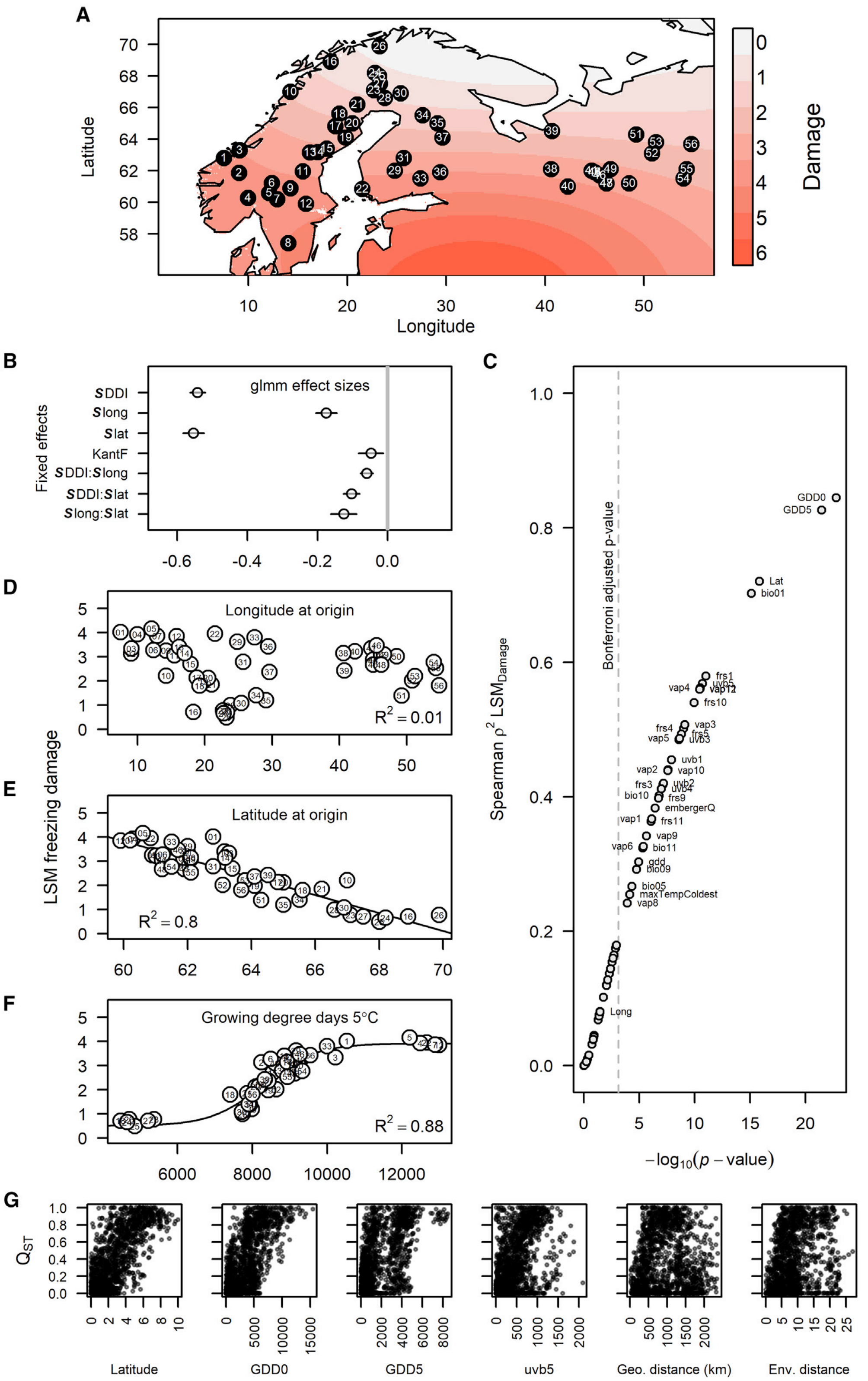

Figure 1. Phenotypic variation and environmental correlations.
(A) The fitted damage level surface across the sampled range based on simple kriging of the least-squares means estimates of population damage levels from the GLMM model.

(legend continued on next page)

frequencies associated with the trait variation is difficult, especially if the underlying genetic structure is unknown (Latta, 1998; Le Corre and Kremer, 2003; Hall et al., 2007; Yeaman, 2015). An advance in the field will likely come from coordinated investigations at both the phenotypic and the genomic levels with comprehensive sampling and a systems approach.

Scots pine is one of the most widely distributed conifers in the Northern Hemisphere (San-Miguel-Ayanz et al., 2016). The widespread occurrence of the species demonstrates an ability to adapt over spatially heterogeneous environments, e.g., large temperature and photoperiod variations (Rehfeldt et al., 2002; Jankowski et al., 2017). A clear illustration of local adaptation is that northern populations set buds and develop autumn frost tolerance significantly earlier than southern populations (Hurme et al., 1997; Andersson and Fedorkov, 2004; Savolainen et al., 2004; Jankowski et al., 2017). Autumn frost tolerance, ‘‘cold hardiness’’ hereafter, is a critical fitness component in the northern climate as it determines the mortality and survival of seedlings and thus the success rates of forest regeneration. The underlying genetic bases of these trait variations are still poorly investigated.

Scots pine likely recolonized Scandinavia after the LGM from the south and the Russian Plain (Bennett et al., 1991; Cheddadi et al., 2006; Tóth et al., 2017). However, macrofossil and ancient DNA evidence suggests that several tree species arrived in the north remarkably soon after the deglaciation and thus points to the possible existence of cryptic refugia on the edges of the ice sheet at high latitudes (Kullman, 2008; Parducci et al., 2012; Zale et al., 2018). This hypothesis would imply that a discrete genetic cluster of Scots pine may be present in the north. A complex demographic history can contribute to genetic heterogeneity over the distribution and complicate the inferences of other evolutionary processes in generating spatial diversity. The population genetics literature on Scots pine is extensive (see Pyhäjärvi et al., 2020, for review and literature therein). Despite these continuous efforts, large-scale sampling for phenotypic and genetic variation across the distribution is still lacking. It has also not been determined whether a fine-scale population structure exists in Scandinavia as a result of migration from the various refugia. This knowledge would inform us about the past history and refine both the detection power and the conclusions drawn from genome-wide associations (Barton et al., 2019).

In this study, we sampled 54 Scots pine populations from the Norwegian coast over the Arctic Circle to western Russia, covering 47.3 longitudes or more than 1/8 of the earth's circumference, which represents the most comprehensive coverage of northern Europe to date. We inferred variation in autumn phenology and dormancy progression from freeze-hardiness tests conducted on >5000 seedlings, of which >900 seedlings from 24 populations were genotyped using genotyping by sequencing (GBS). Our main goal was to evaluate adaptive responses in Scots pine at the phenotype and genotype levels. Evaluation of cold hardiness along environmental and geographical gradients would contribute to an understanding of the performance of these gradients for predicting freeze-damage levels. The genotype data allow evaluation of genetic variance across landscapes and thus shed light on the degree of genetic–environmental association and the recolonization history of Scots pine in Scandinavia. We found patterns in the allele frequencies that parallel the freeze-damage levels, although the generally small allele frequency shifts observed across the range could not be separated in attribution between geographic and environmental variables. More extensive investigations may provide further insights into the genomics of adaptive trait variation and aid the projection of evolutionary responses to climate change.

RESULTS

Cold-hardiness variation

The two major goals of this study were to understand the distributions of: (1) cold hardiness and (2) genotypic variances across the northwestern (NW) distribution range of Scots pine (Figure 1A). To achieve the first goal, we raised more than 5000 seedlings (Supplemental Table 1) in a common garden setting. We subsequently subjected these seedlings to increasing night length to initiate dormancy and finally exposed them to freezing temperatures to assess their frost hardiness through their damage levels.

The damage levels (0–6) of seedlings from each population were assessed at 10 specific time points after dormancy initiation. Because damage was scored categorically, we assumed a Poisson-distributed response variable and fit it using a general linear mixed model (GLMM) (see Materials and methods). The final GLMM showed that latitude at origin and number of days since initiation of dormancy (DDI) had the largest impacts on freeze damage (Figure 1B). Because DDI, latitude, and longitude were standardized (see Materials and methods), the effect size estimates do not translate 1:1 to the actual impact they had on damage levels; +1 in standardized latitudes decreases damage by 0.55 on average and a unit increase in standardized DDI decreases damage by 0.54 (Figure 1B), which corresponds to an average reduction of the damage scores by 0.22 for every +1°N and 0.06 for every additional day of dormancy progression. Longitude also had an effect where an increase in standardized longitude decreased damage levels by 0.18 on average (Figure 1B), which translates to a 0.012 damage reduction for every longitudinal degree east. Slightly higher damage scores were observed

(B) Estimated sizes of significant fixed effects from the GLMM model on freeze damage with their 95% CI. DDI, days since dormancy initiation; lat, latitude; long, longitude; and KantF, position of the seedling in the freezer. Uppercase ***S*** preceding effect names indicates that variables have been standardized before analysis.

(C) Squared Spearman's ρ between least-squares means of damage levels and climate variables (see Supplemental Table 2), including latitude and longitude.

(D–F) The explanatory ability of **(D)** longitude, **(E)** latitude, and **(F)** growing degree days above 5°C for freeze-damage variation among populations.

(G) Pairwise Q_{ST} versus pairwise differences in latitude, growing degree days above 0°C (GDD0), GDD5, uvb5 (the four variables that correlate best with pairwise Q_{ST}), and physical and environmental distance between populations.

along the Norwegian coast at all latitudes, leading to a negative interaction between latitude and longitude (Figure 1A). This likely reflects the transition from the milder, Gulf Stream-influenced, maritime climates at higher latitudes along the Atlantic coast toward the increasingly continental climates of the east, where the onset of seasonal change is more rapid. We also detected a small position effect of the seedlings in the freezer (KantF): seedlings farther from the edge (i.e., more protected from freezing by neighboring seedlings) had reduced damage. Both longitude and latitude interacted negatively with DDI (Figure 1B), indicating a more rapid cold-tolerance progression with increasing longitude or latitude. All the above-described effects are highly significant from zero (Figure 1B), with $p < 10^{-10}$, except for KantF, which had a p value of 0.0073.

Latitude is expected to have a high correlation with the length of the growing season and is often used as a proxy for climate in boreal settings. However, because longitude was also a significant factor, we expected that the actual climate at origin would be a better predictor of freeze damage (Andersson and Fedorkov, 2004). We then examined the statistical relationship of 68 climate variables (Supplemental Table 2) with freeze damage. In addition to latitude, growing degree days above 0°C (GDD0) and above 5°C (GDD5) and annual mean temperature (bio01) correlated well with the variation in freeze damage, but longitude did not (Figure 1C and 1D). In this study, the freezing time points were optimized to give the highest resolution in freeze damage around the average populations. This means that the resolution among the most extreme populations is reduced, and both extremes of the hardiness distribution level off, producing a response that is more sigmoidal than linear. Both GDD0 and latitude appeared to follow a more linear trend. When the data were fit to a sigmoid distribution, GDD5 (non-linear adjusted R^2 = 0.883) explained more of the variance in freeze damage than either latitude (linear adjusted R^2 = 0.805) or GDD0 (non-linear adjusted R^2 = 0.853, data not shown; Figure 1E and 1F).

The principal-component analysis (PCA) on all 68 climate variables reduced the environmental space to three dimensions that explained 80.2% of the environmental variance among populations. The Norwegian coast appeared to possess unique environments resulting in greater environmental distances to all other populations (Supplemental Figure 1). In line with the strong clinal variation across latitude, GDD5, GDD0, and bio01, we observed clear divergent selection among populations on cold hardiness, with a point estimate of global Q_{ST} = 0.82 (95% highest [posterior] density interval [HPDI] 0.51–0.95). Pairwise Q_{ST} among populations correlated best with latitude, GDD0, and GDD5 (Spearman's ρ = 0.72, 0.69, and 0.6, respectively), but not with the overall environmental distance, longitude, and geographic distance *per se* (ρ = 0.31, 0, and 0.13, respectively, Figure 1G).

Genetic diversity

To achieve our second goal, we genotyped 941 seedlings from 24 selected populations (Supplemental Table 1) using GBS. The average number of reads per individual was 2.36 M with a sequence depth 87×, and a mapping rate of 96.36% to the reference genome *Pinus taeda* (Supplemental Table 3). We first screened for highly related individuals in the samples because their presence can inflate population structure. We identified 195 individuals with a relatedness greater than or equal to first cousin (Supplemental Table 4). Removing the related and replicated individuals and re-performing SNP filtering (see Materials and methods) gave us 855 487 SNPs. After removing non-polymorphic loci we had 85 573 SNPs left that, with a minor allele frequency <0.05 filter, shrank down to the final number of 10 925 SNPs for the remaining 746 individuals (Table 1). This set of individuals and SNPs was then used in diversity and population structure analyses.

The overall observed heterozygosity H_o was 0.2487, while expected heterozygosity H_e was 0.2803. All populations had similar values for H_o and H_e with no significant departures from Hardy–Weinberg proportions, as shown by the within-population fixation index F_{IS} (Table 1), in accordance with the high outbreeding of this species (Hall et al., 2020). The mean nucleotide diversity (π) for all sites was 0.0045 (Table 1), 0.0032 at zero-fold degenerate coding sites (π_0), and 0.0066 at four-fold sites (π_4). All populations had similar levels of diversity, except population 8 (southern Sweden), with slightly elevated estimates in both H_o and nucleotide diversity estimates.

Population structure

Using all 10 925 SNPs, we measured differentiation among populations using the fixation index (F_{ST}). Pairwise F_{ST} across all populations showed little variation and followed a normal distribution N (μ = 0.0040, σ = 0.0063, Supplemental Table 5), with a global estimate of 0.0037. Despite being low, F_{ST} increased with increasing geographic and environmental distances between populations (adjusted R^2 = 0.31 and R^2 = 0.041, respectively, both with $P < 0.001$, Figure 2A and 2B), indicating a pattern of IBD. Because of this IBD signal, we investigated whether we could detect possible ancestry components (K) over the sampling space using TESS3 (Caye et al., 2016). TESS3 suggested an optimal K from 1 to 3, although increasing K above 1 had only a marginal effect. It appears that at K = 3, the Russian populations displayed an ancestry composition that differed from the Fennoscandian populations (Figure 2C and 2D). We further examined population structure using *conStruct*, which considers both continuous and discrete processes. Cross-validation tests indicated that the spatial model that accounts for IBD is preferred over the discrete non-spatial model, and that one ancestral component is broadly sufficient to describe the global structure in our data (Supplemental Figure 2).

PCA of the overall distribution of genetic variation revealed a similar pattern with a generally low discrimination power among populations but a discernable trend along longitude (PC1, R^2 = 0.85) and latitude (PC2, R^2 = 0.58, Figure 2E). Only 1.17% of the genetic variation was explained by the first two PC axes. All these results suggest a very weak population structure in Scots pine across Fennoscandia and western Russia.

To explore the possible forces on differentiated loci, we searched for significant F_{ST} outliers using BayeScan (Foll and Gaggiotti, 2008) and TESS3 (Caye et al., 2016), which control for

Population ID	Country	N	H_o	H_e	F_{IS}	Nucleotide diversity π		
						All sites	Zero-fold sites	Four-fold sites
1	Norway	38	0.2683	0.2811	−0.0570	0.0045	0.0035	0.0065
2	Norway	18	0.2321	0.2931	0.0783	0.0044	0.0035	0.0064
3	Norway	40	0.2619	0.2791	−0.0163	0.0044	0.0035	0.0064
4	Norway	34	0.2729	0.2827	−0.0129	0.0045	0.0035	0.0066
8	Sweden	46	0.3026	0.2877	−0.1491	0.0050	0.0040	0.0076
10	Norway	49	0.2596	0.2798	−0.0159	0.0044	0.0035	0.0065
12	Sweden	56	0.2478	0.2795	0.0178	0.0045	0.0035	0.0064
13	Sweden	19	0.2338	0.2973	0.0608	0.0045	0.0036	0.0065
16	Norway	62	0.2288	0.2762	0.0330	0.0044	0.0035	0.0064
22	Finland	33	0.2517	0.2818	−0.0303	0.0044	0.0035	0.0066
26	Norway	59	0.2116	0.2780	0.0802	0.0044	0.0035	0.0064
30	Finland	20	0.2444	0.2903	0.0775	0.0045	0.0036	0.0064
31	Finland	16	0.2555	0.2982	0.0222	0.0044	0.0035	0.0065
33	Finland	10	0.2937	0.3181	−0.0213	0.0044	0.0034	0.0064
34	Finland	19	0.2764	0.2888	−0.0304	0.0045	0.0035	0.0064
37	Finland	64	0.2470	0.2780	0.0082	0.0044	0.0035	0.0065
38	Russia	15	0.2327	0.3018	0.0896	0.0044	0.0035	0.0063
39	Russia	12	0.2863	0.3201	−0.0888	0.0045	0.0036	0.0066
40	Russia	18	0.2662	0.2927	−0.0041	0.0044	0.0035	0.0064
47	Russia	20	0.2642	0.2886	0.0127	0.0045	0.0035	0.0066
51	Russia	19	0.2632	0.2935	−0.0334	0.0044	0.0035	0.0066
52	Russia	18	0.2507	0.2927	0.0272	0.0044	0.0035	0.0064
55	Russia	53	0.2567	0.2785	−0.0383	0.0044	0.0035	0.0065
56	Russia	8	0.3260	0.3356	−0.0483	0.0044	0.0034	0.0065
Overall		746	0.2487	0.2803	−0.0048	0.0045	0.0036	0.0066

Table 1. Summary of genetic diversity.

Heterozygosities (H_o and H_e) were measured as an average over all polymorphic sites in each population, whereas both monomorphic and polymorphic sites were used when calculating nucleotide diversity (π). Fixation index F_{IS} was calculated and tested by 1023 permutations of gene copies between individuals within each population. N is the number of seedlings remaining in each population after removing highly related individuals (see Supplemental Table 4 for distribution of related individuals).

geographic relationships between populations. Given the overall low differentiation, BayeScan identified 164 highly significant loci with a probability of 1 of being differentiated ($F_{ST} > 0.0491$; Bayes factor > 1000; Figure 3A), while TESS3 detects no significant outliers. These contrasting results imply that geographic relationships between populations are an important factor in the differentiation detected by BayeScan. Further examination of the allele frequency origins of these outliers indicated three major underlying haplotype components with a finer-scaled structure among populations. One cluster was of eastern origin, the second cluster was centered in NW Norway, while the alleles for the third cluster appeared only in southernmost Sweden (Figure 3B). The main drivers of this clustering are likely centered on the apparent distinct allele frequency differences among regions (Supplemental Figure 3). The southern cluster had a high frequency of one of the alleles at each of two loci (these loci are colored blue in the figures), while all other populations were almost fixed for the other allele. The same is true for eight other loci (colored orange) in the three NW populations. There are no apparent relationships between the ancestral components in outliers and the geographical variation in autumn frost tolerance (Figure 1A).

Genetic–environmental association

To understand whether allele frequency variation could be associated with environmental variation, we compared allele frequencies among populations with their latitude, longitude, and climate variables using direct correlations and redundancy analysis (RDA). The overall allele frequencies were most strongly correlated with longitude and weakly with latitude (Figure 3C). Rank (Spearman) correlations among the allele frequencies of all loci to all 68 climate variables identified 26 loci that showed elevated correlations with 12 environmental variables (Figure 3D).

For RDA, we first performed forward selection on the initial 68 environmental variables and the principal coordinates of neighbor matrices (PCNM) separately. This step selected bio8

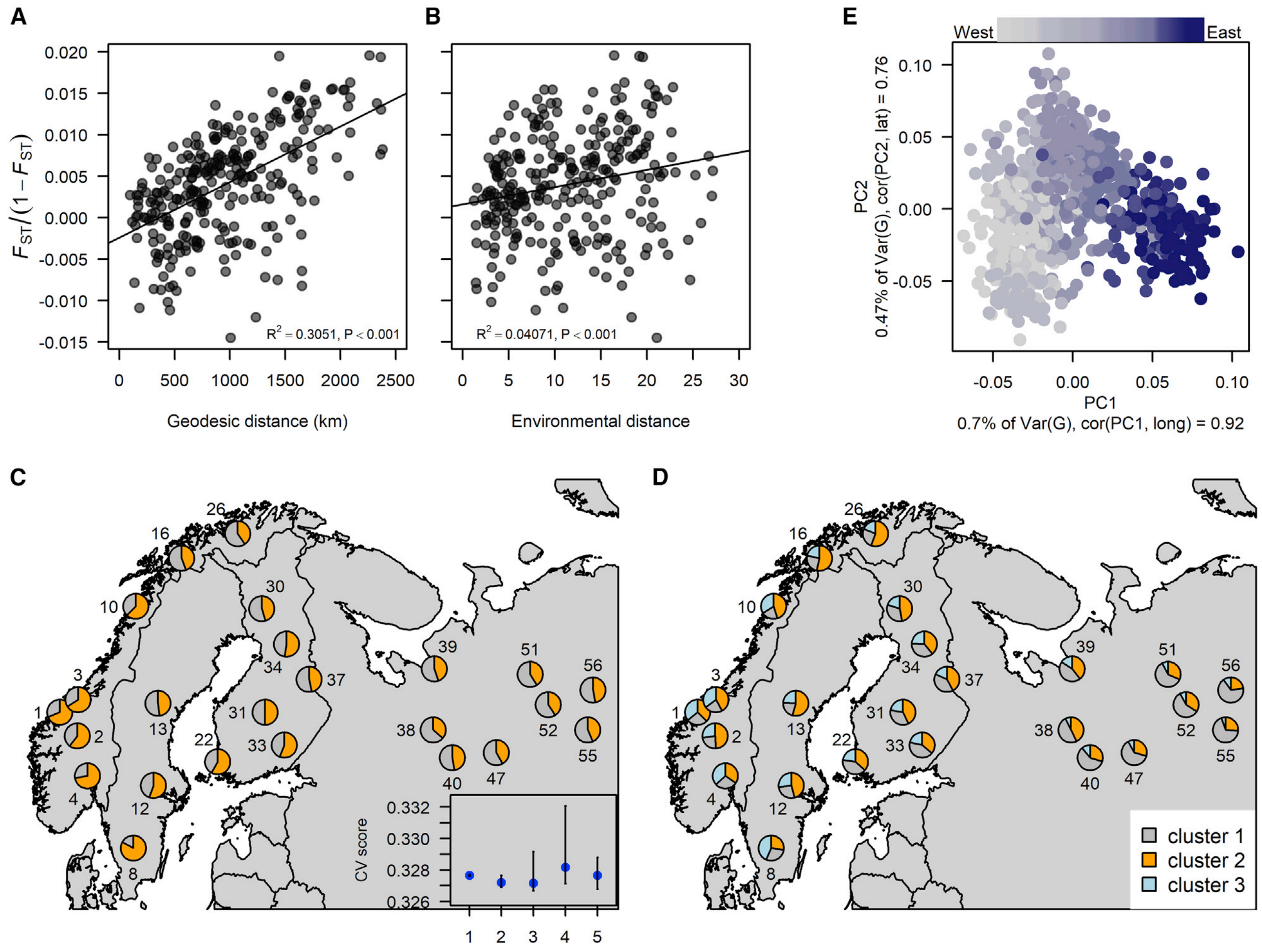

Figure 2. The spatial genetic structure across the genotyped populations using all SNPs.
(A and B) The pairwise genetic differentiation across **(A)** geo-distance and **(B)** environment.
(C and D) show the pie plots of genetic composition for $K = 2$ **(C)** and $K = 3$ **(D)** from *TESS3*. The blue points in the inlaid graph in **(C)** indicate the cross-validation score with the lowest root-mean-square error between the genotypic matrix and the fitted matrix among replications, for $K = 1$ to 5.
(E) PCA of all genotyped seedlings, colored according to their longitudinal origin, with the darker color farther east. The two first PCA axes explained 1.17% of the genetic covariances among the seedlings. The PCA axes are also highly correlated with longitude (PC1) and latitude (PC2).

(mean temperature of wettest quarter), GDD0, and wet11 (wet day frequency in November) for the environmental matrix and four PCNM axes (1, 5, 11, and 13) for the geographic matrix. The full RDA model with these eight variables was significant and explained 17.4% of the total variance observed among populations (adjusted R^2 = 17.4%, P = 0.001). Partitioning the variances for environmental and geographic impacts revealed a confounding effect of 8.1% between the two groups of factors, and environmental variables alone explained 2.9% and geo-distance 5.8% of the variation among populations (Figure 3E).

RDA performed on the environmental matrix alone identified the first two RDA axes as significant (P = 0.001 and 0.006) and explained about 5.5% and 2.9% of the variance, respectively. Maximum temperature of the coldest month is the most influential variable, with a correlation of −0.90 with RDA1. This variable had, in turn, a large negative correlation with longitude, Spearman's $\rho = -0.88$. Outlier analysis on the two significant RDA axes detected 154 significant SNPs, of which 38 were among the 164 BayeScan outliers (Figure 3F and 3G). Of the 38, 13 also showed high allele frequency correlations with environmental variables (Figure 3D). The strongly differentiated NW outliers, on the other hand, were mainly along the fourth non-significant RDA axis (Figure 3F and 3G), indicating that some environmental factors might be linked to their allele frequency shifts, but the signals are weak. Furthermore, no significant loci were detected when controlling for geographic distance in partial RDA, demonstrating that environmental and geo-distance are confounded in their influence on allele frequencies. The fact that RDA on the environment detected numerous outlier loci and that environmental factors by themselves explained about 3% of the allele frequency shifts implies that there could be adaptive differentiation among loci in our dataset, although pinpointing the environmental variables shaping their allele frequency distribution and disentangling them from the geographic relationships would be difficult.

Association mapping

We found strong indications of footprints in the allele frequency gradients that mirror adaptation to cold hardiness, and both BayeScan

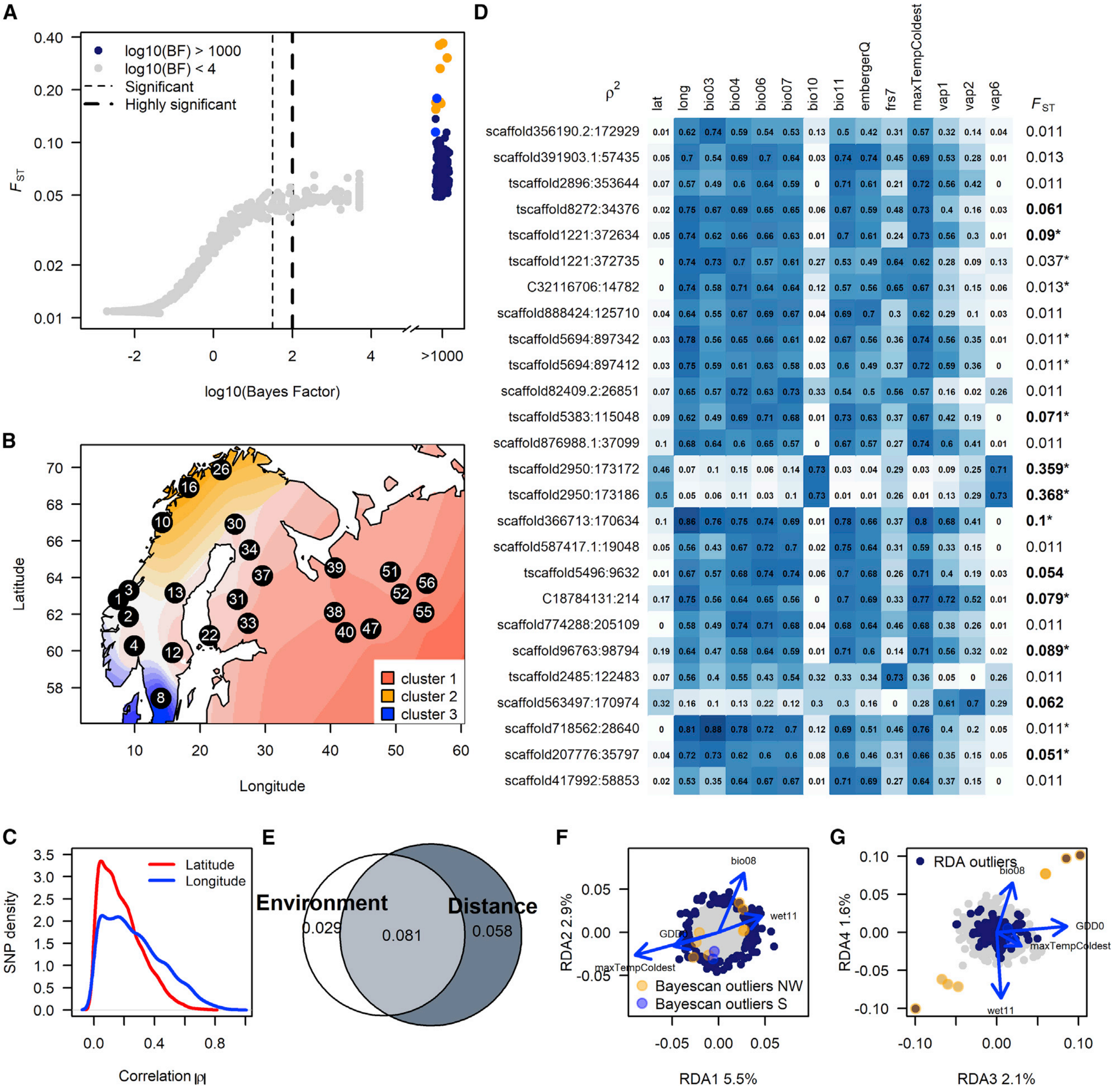

Figure 3. Detection of highly differentiated loci across the sampled range and environments.
(A) BayeScan detected 164 outlier loci with a $\log_{10}$(Bayes Factor) $\geq$ 1000 and F_{ST} values ranging from 0.049 to 0.368. The highly differentiated loci specific to NW populations are marked in orange and the outliers in the southern cluster in blue. The *y* axis is on a logarithmic scale.
(B) Distribution of major ancestry components in the 164 BayeScan outlier loci.
(C) Distribution of absolute allele frequency Spearman's rank correlations (|ρ|) with latitude (red line) and longitude (blue) across all loci.
(D) Squared correlation plot of 26 SNP and 12 environmental variables that had a $\rho^2 > 0.7$; latitude and longitude are included for reference. F_{ST} values in bold are significant outliers in BayeScan; those with an asterisk are outliers in the environmental RDA model.
(E) Proportion of variance explained by the environmental versus the geographic distance matrix in the full RDA model (see Materials and methods).
(F and G) RDA of population allele frequencies in response to the most influential environmental variables in the first four constrained axes, of which **(F)** RDA1 and RDA2 are significant. **(G) RDA3 and RDA4.** Midnight blue points are outliers. However, no outliers were detected when geographic distance was accounted for.

and RDA indicated the possibility that some loci have been under selection. For example, those alleles that correlated strongly with freeze damage scores also correlated with latitude and the length of growing season (GDD5), but not with longitude (Figure 4A–4C), which otherwise appears to drive most allele frequency shifts. One of these loci, scaffold 563497:170974, displayed the highest explanatory power with freeze damage (linear model fit: R^2 = 0.61, adjusted R^2 = 0.596. Supplemental Figure 4).

To explore the possible association between genotypes and hardiness, we ran association mapping on seedlings' quantile normalized damage scores for two datasets: the full set of 24

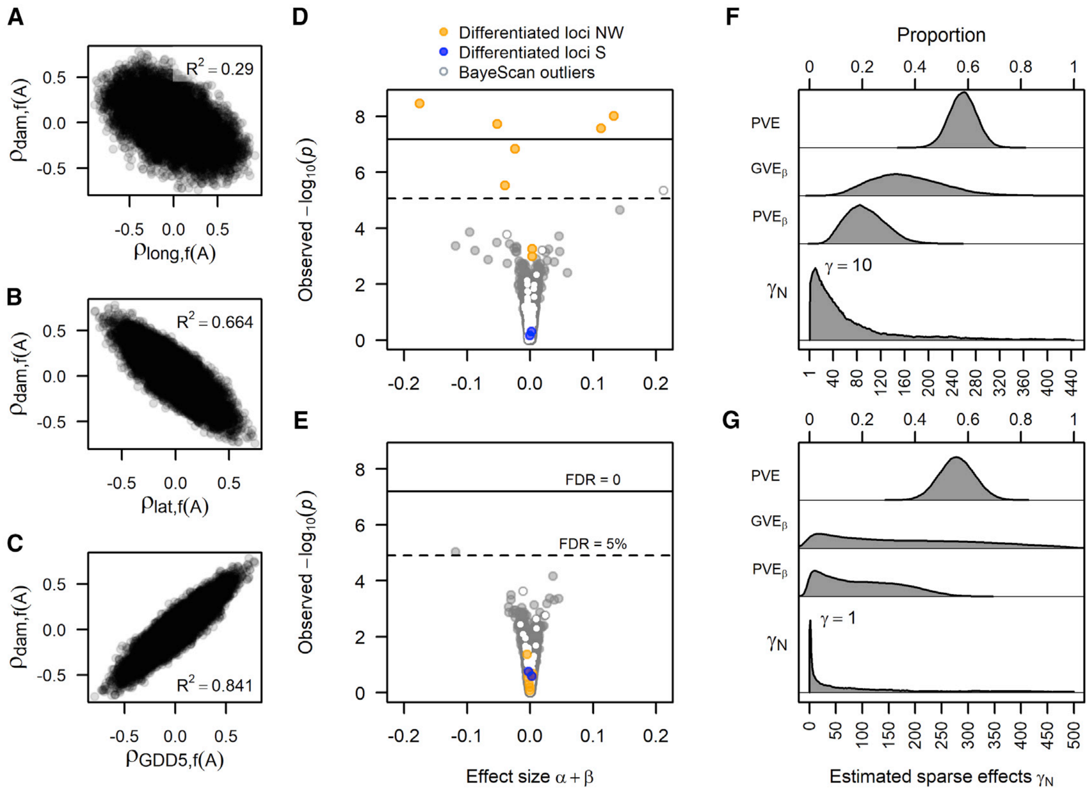

Figure 4. Allele frequency correlation with damage levels and genotype association with damage levels and environmental variables.
(A–C) (A) The allele frequency rank correlation relationship between longitude–allele correlation and damage–allele correlation. **(B)** Latitude–allele correlation versus damage–allele correlation and **(C)** GDD5 and damage. Association mapping was performed on two datasets, the full population set and one excluding the NW populations.
(D and E) Association mapping with damage levels for the **(D)** full and **(E)** reduced datasets. SNP effect size was estimated from BSLMM for the small effect size (α) found in each SNP and the major effect detected for some markers (β). *P* values and false discovery rate (FDR) are from the LMM. Colored points (orange, blue, and white) are the 164 BayeScan outlier loci, of which orange points are those unique to NW populations (10, 16, and 26) and blue are those unique to the southern population (8).
(F and G) (F) The posterior distribution of the estimated genetic effects (top axis) and number of major effects (γ_N, bottom axis) from the MCMC runs in the BSLMM for the full and **(G)** reduced datasets. PVE is the estimated marker heritability.

populations and a second set without the NW populations 10, 16, and 26. The univariate linear mixed model (LMM) (Zhou and Stephens, 2012) in GEMMA (Genome-wide Efficient Mixed-Model Analysis) identified a group of highly significant loci in the full dataset and some with elevated effect size based on the Bayesian sparse linear mixed model (BSLMM) (Zhou et al., 2013, Figure 4D). Interestingly, most of these significant loci were the NW outliers. When the NW populations were removed, the signals of association disappeared, and none of the BayeScan outliers were significant or had elevated effect sizes (Figure 4E). Despite the lack of major effect loci in the reduced dataset, the marker estimated heritabilities of hardiness (*PVE*) were similar, h^2 = 0.58 and 0.56, in the full and reduced population sets, respectively (Figure 4F and 4G, and Supplemental Table 6). This indicates that the combined minor effect size of all loci in the study captured a majority of the additive genetic variance in the trait. We also observed that the major genetic effects contributed around 19% (HPD point estimate PVE_β) of the phenotypic variance in the full dataset. However, the sparse effect estimates (GVE_β) showed no convergence in the reduced population set, as indicated by the large HPD interval and a point estimate close to zero (Figure 4G and Supplemental Table 6). This further suggests that there are no clear major genetic signals from any marker and that the loci detected as significant when NW populations were included are likely due to a large confounding effect between their extreme allele frequency shifts and their extreme environment.

Overall, our association results suggest that the markers captured a large part of the genetic variance in autumn frost tolerance among samples, but the identification of any single locus with identifiable effects on the trait is at best ambiguous. The small number of pseudo-randomly selected markers used in this study, compared with the genome size of Scots pine, makes it unlikely that we would catch any true large effect markers.

DISCUSSION

Cold-hardiness variation and local adaptation

Quantitative and qualitative assessment of local adaptation is an essential step toward understanding the major evolutionary forces operating in natural populations. The common approach is to establish the relationships between phenotype, genotype, and environment and their interactions under (semi)controlled

 © 2020 The Author(s).

conditions to identify the selective agents underlying the trait variation. Estimating the degree of local adaptation through phenotypic measurement is often an extensive endeavor, especially for species with long generation times and vast distribution ranges. In forestry, cold hardiness of seedlings guides their deployment to correct climate zones and thus has operational cost–gain consequences. This is particularly true in northern Scandinavia, where the most important trait for production forests is survival (Berlin et al., 2009; Persson et al., 2010). Autumn frost hardiness in young seedlings has an estimated narrow sense heritability of 0.3–0.54 (Persson et al., 2010) and is an important target in breeding programs for northern climates (Andersson, 1992).

We observed increased and more rapid progression of frost tolerance in populations from harsher climates and a high estimate of Q_{ST} 0.82 among the sampled populations, illustrating strong divergent selection on this trait. If we account for the marker-estimated heritability of 0.56, which is somewhat higher than previously reported, the global phenotypic differentiation would be even higher, Q_{ST} = 0.9 (95% HPDI 0.65–0.97, see Materials and methods). High Q_{ST} values for phenology-related traits have also been reported for other tree species, e.g., Norway spruce (Milesi et al., 2019), downy birch (Bennie et al., 2010), European beech (Strømme et al., 2019), and European aspen (Hall et al., 2007). Analysis of phenotype–environment associations with 68 climate variables identified mainly those that describe the temperature over the year, GDD5, GDD0, bio01, and latitude. Latitude at origin has often been used as an explanatory variable in studies of clinal phenotypes and allele frequencies, e.g., insects (Oakeshott et al., 1982), birds (Johnsen et al., 2007), and plants (Stinchcombe et al., 2004; Ma et al., 2010). However, we also observed significant differences in frost tolerance among populations from the same latitudinal origins, but no differences when comparisons were based on length of the growing season at origin. We found that the phenotypic estimates and interactions with climate variables are complex, and that a simple experimental design along a latitudinal gradient would not capture all interactions with frost hardiness. Additional aspects of this phenotypic variation become evident when measured over a larger range across a boreal setting, indicating that the usual proxies of climate are suboptimal.

Spatial genetic variance

Linking phenotypes and environmental factors to genetic variation requires a correct estimate of population structure, even at finer spatial scales (Barton et al., 2019). Similar to the importance of analyzing phenotypes across the appropriate explanatory variables to facilitate the separation of confounding factors, the assessment of genetic pattern would suffer from limited spatial coverage. Although we had a gap in our sampling, from the Karelia and Murmansk regions between Finland and Archangelsk, we detected a gradual change in genetic composition from east to west, with one ancestral component predominant in Russia and one in Scandinavia. This is suggestive of a major migration route from east to west. However, accounting for IBD, the gradual change in allele frequencies could be explained by a continuous spatial model with one ancestral component. A global F_{ST} of 0.0037 is much lower than that in Norway spruce (*Picea abies*), which also has a large distribution and a postglacial migration history similar to that of Scots pine. In Norway spruce, strong genetic differentiation is observed between the northern and the central parts of Europe (F_{ST} = 0.15–0.22; Chen et al., 2019; Milesi et al., 2019), as well as at a regional scale in northern Europe (Tollefsrud et al., 2009; Tsuda et al., 2016; Li, 2020; Sullivan, 2020). Our observation of weaker population structure in Scots pine in northern Europe, with no distinct subgroups, is in accordance with previous studies of Scots pine population structure (Dvornyk et al., 2002; Pyhäjärvi et al., 2007; Zimmer and Sønstebø, 2018; Tyrmi et al., 2020).

The very high and uniform diversity across Scots pine populations implies effective gene flow over large distances, which in turn could mask possible footprints of admixture in a population with recurrent migration from several refugia. However, the slightly elevated nucleotide diversity in the southern Swedish population (Table 1) could indicate an admixture of gene flow from two migratory routes, similar to what is observed in Norway spruce (Chen et al., 2019). In contrast to hardiness variation, which is associated with latitude, we found that the factors that most strongly correlate with and shape allele frequencies are longitude and environmental variables that follow a longitudinal gradient. In line with this, we found that the genetic variance explained by environmental variables overlaps the variance explained by spatial variables to a large extent. Longitude explained almost 50% more of the genetic variance than latitude in the PCA (Figure 2E). This suggests that most neutral allele frequency shifts, albeit small, are due to a longitudinal migratory route together with effective admixture, possibly over multiple post-Ice Age recolonizations.

In contrast to the weak structure in the nuclear genome, mitochondrial DNA markers have shown distinct differentiation in Scots pine from western Europe to eastern Russia (Naydenov et al., 2007; Dering et al., 2017). Mitochondrial DNA markers are maternally inherited through seeds and can often reveal otherwise hidden demographic components due to the low dispersal ability of seeds (Pyhäjärvi et al., 2008; Semerikov et al., 2018). Unfortunately, the resolution so far is low from the detected mitochondrial variation in Scots pine, nonetheless two distinct mitotypes have been found in Fennoscandia (Dering et al., 2017), and the pattern is inconclusive as to whether a refugium existed in northern Scandinavia under the LGM (Kullman, 2008; Parducci et al., 2012). Interestingly, Parducci et al. (2012) recovered 20 000 year old ancient *P. sylvestris* DNA and macrofossils in lake sediments on the island Andøya, situated just west of our population 16. This finding indicated a possibility of recovering a genetic signal in current populations. To bridge this knowledge gap, we managed to obtain material from a large part of Norway, and in particular NW Norway, and scored a substantial number of nuclear markers. This expanded sampling allowed us to identify a greater number of differentiated loci than in other recent studies (e.g., Tyrmi et al., 2020). Further mapping of these loci provided hints of three major haplotype structures underlying their allele frequency differences. Although the signals are weak, the NW-most populations appear to harbor alleles from an isolated lineage with otherwise rare alleles at or almost at fixation (populations 10 and 16, respectively). Possible scenarios for this to

occur are genetic drift or selection. Genetic drift would be more effective when gene flow into these marginal populations is restricted. However, for almost all loci the differentiation is basically zero, implying large gene flow among populations in general. For this drift scenario to be plausible, given the observed allele frequency distribution, the simplest explanation would be that some loci were located in a genomic region that experienced rearrangement during drift. This would produce a segment with greatly reduced recombination rate relative to all other populations. Thus, when the gene-flow barrier dissipated, the high frequencies would be maintained for a few loci, despite the high gene flow observed across the range since LGM, a corresponding time period of a few hundred or so generations. If selection, on the other hand, has shaped these extreme allele frequency differences, we would expect very strong selection favoring the rare allele in these populations, but equally strong against them in all other populations, given the extremely high gene flow observed. Unfortunately, we cannot examine these hypothesis further with the data currently at hand.

Genotype–environment and genotype–phenotype associations

The aim of association studies is to understand the underlying causes and biological nature of the variation in traits so that this information can be used to predict responses to future conditions and facilitate selection in breeding. However, association mapping in the wild for polygenic traits is challenging due to confounding factors and complex genetic interactions, resulting in typically weak association and predictive power (Nadeau et al., 2016; Barton et al., 2019). The influence of genes and environment on important quantitative traits remains elusive in conifer species.

In this study, we detected 164 significant F_{ST} outlier loci. Some of the loci correlated strongly with both environmental variables and longitude, but none were significant in the genotype–phenotype mapping. A similar result is reported by Tyrmi et al. (2020), who obtained almost eight times as many SNPs from exome capture (although still a minor part of the genome), but detected only one significant outlier. The most extreme F_{ST} outliers in our study are limited to the Norwegian marginal NW distribution range. The same extreme outliers are the only loci that appear to have a significant effect on freeze damage, but the signal from these loci disappeared completely when the NW populations were excluded from the analysis. It is also striking that, compared with BayeScan, neither TESS3 nor RDA detected any outlier loci when accounting for geography. From this we conclude that it is more probable that none of the loci we analyzed are more strongly influenced by any environmental factor than as a result of colonization or that they individually control a substantial proportion of frost hardiness. However, the great number of shared allele frequency correlations between freeze damage and length of growing season, rather than the east–west colonization route, is also suggestive of the presence of many small allele frequency shifts that have been shaped by local adaptation. The high marker-estimated heritability of frost hardiness (h^2 = 0.56) corroborates this hypothesis. This is a promising finding illustrating that, collectively, our markers captured a large portion of the trait variation and inheritance. The weak association signals at individual loci are not surprising, given the small and random portion of the genome sampled, the large effective population size of Scots pine, and the polygenic architecture of quantitative traits. In such cases, the statistical power of detecting any association is very limited unless the sampling of markers and phenotypes is extraordinarily large (Hall et al., 2016; Gienapp, 2020).

Overall, this study reveals several suggestive results that further studies may shed light upon: (1) The evolutionary forces that shape the strong correlation of some outliers with a locally adapted trait and climate should be studied. With an east–west colonization route almost perpendicular to the adaptive cline and the low neutral differentiation observed, the population genomics of Scots pine should facilitate detection of loci under divergent selection given a larger representation of the genome. (2) The three underlying haplotype structures among differentiated loci across the range (see Supplemental Figure 3, and Tyrmi et al., 2020) could have been caused by differential linkage disequilibrium between these loci in some populations. The fixation of otherwise rare alleles in the NW populations despite extensive gene flow indicates a reduction in recombination around these loci, possibly from an inversion, that would allow for these alleles to drift to fixation. Another possibility would be that differential selection on these or adjacent linked loci in a low recombination area brings these rare alleles to high frequencies. The common rare alleles in the southern population possibly originate from admixture with continental alleles, due to migration routes similar to those observed in *P. abies* (Chen et al., 2019). Whether this is the case or whether it is due to some other phenomena clearly needs further validation. (3) Finally, capturing a large proportion of genetic variance in the trait could potentially lead to genomic prediction in natural populations. However, whether we would actually then estimate the origin of the population as a function of the spatial matrix rather than the actual adaptation of the population to the local environment remains to be explored. Nevertheless, Scots pine promises to provide an excellent genomic background to advance our understanding of how allele frequency shifts are shaped by evolutionary forces.

MATERIALS AND METHODS

A more detailed Materials and methods is provided in the Supplemental information.

Sampling and freezing test

To obtain a comprehensive view of autumn frost-tolerance (or cold-hardiness) variation, genetic diversity and possible genetic structure of the NW distribution range of Scots pine, *P. sylvestris* L., we collected 54 populations ranging from Norway to western Russia and covering latitudes 57.5°N–69.1°N (Figure 1, Supplemental Table 1). Fifty-three of these populations were freeze tested. Seedlings were grown in a greenhouse at +20°C. Each seedling's position in the seedling box was mapped for calculation of edge effect (KantF). From week 10, the length of the dark period was increased by 1 h every week to initiate bud dormancy. Seedling boxes were placed in a freezer chamber at 10 different time points after the DDI treatment, for 2 h at −10°C. The freeze damage on each seedling was evaluated at least 1 week after to allow for discoloration to develop. The degree of freeze damage was scored visually into seven classes: 0, no needle discoloration; 1, 1%–20% of needles discolored; 2, 20%–40%; 3, 40%–60%; 4, 60%–80%; 5, 80%–99%, and 6, all needles discolored. This testing protocol was established in the early 1980s by the

Forestry Research Institute of Sweden (Skogforsk) in Sävar (Andersson, 1992; Persson et al., 2010) as a standard method for monitoring the hardiness of Scots pine seed orchard crops for reforestation. Needles were collected from seedlings for genotyping before they were subjected to freeze exposure.

Phenotypic analyses

Freeze damage was analyzed using a GLMM with the "glmer" function in the R package "lme4" (Bates et al., 2015). We assumed a Poisson-distributed response variable of damage categories as a function of the fixed effects of placement in the freezer (KantF), DDI, longitude, and latitude at origin, and their interactions. Individual plants were treated as a random effects of the population within replication. Longitude, latitude, and DDI were centered (subtracting the mean) and then scaled by dividing by their standard deviations. The average damage levels of the populations were calculated as the least-squares means for each sampled population to remove some experimental effects.

We also sampled the posterior distribution of freeze-damage differentiation among populations to estimate Q_{ST} in this trait with a Markov chain Monte Carlo (MCMC) procedure. Q_{ST} is the quantitative trait equivalent of the F_{ST} (Spitze, 1993; Whitlock, 1999; Leinonen et al., 2013). Q_{ST} for freeze damage was calculated as follows:

$$Q_{ST} = \frac{\sigma_B^2}{\sigma_B^2 + 2h^2\sigma_W^2},$$

where σ_B^2 is the between-population variance estimate and σ_W^2 is the within-population variance estimate (Spitze, 1993; Whitlock, 1999). The σ_W^2 term is confounded and contains the total within-population phenotypic variance, i.e., heritability, $h^2 = 1$. We estimated pairwise Q_{ST} values between populations and compared them with their differences in environmental variables, latitude, longitude, and physical and environmental distances. To calculate environmental distance, we performed a PCA on 68 environmental variables (Supplemental Table 2) and used the decomposition of variables to calculate the Euclidean distance $D_{i,j}$ between populations *i* and *j* in multidimensional space.

GBS library preparations

We extracted DNA for seedlings from 23 populations (Table 1, Supplemental Table 1) using the EZNA SP Plant DNA Kit (Omega Bio-tek). The GBS library was prepared using a *Pst*I high-fidelity restriction enzyme (New England Biolabs), following the protocol of Pan et al. (2015). Briefly, 200 ng DNA from each seedling was digested separately and ligated to sequencing adapters (with individual barcodes) simultaneously. This was carried out at 37°C for 8 h followed by 65°C for 30 min. The digested and ligated DNAs of 300 samples were then pooled into each library, purified, and PCR amplified. Fragment sizes of 350–450 bp were selected using an E-gel EX 2% agarose gel (Thermo Fisher Scientific) and purified. Paired-end sequencing (2 × 150 bp) was performed on Illumina HiSeq X Ten. In each library, we included a few samples as within- and among-library replicates.

Bioinformatics

Sequence read quality was assessed with FastQC (http://www.bioinformatics.babraham.ac.uk/projects/fastqc/). Adapter sequences and low-quality bases (Phred quality <20) from the tail of each read were removed by using Trimmomatic (Bolger et al., 2014). Clean reads were cataloged by using the process_radtags module of Stacks v.2.0 (Catchen et al., 2011) according to individual barcode. Reads shorter than 41 bases were discarded. Sequence reads were aligned to the *P. taeda* draft genome v.1.01 (Neale et al., 2014; Zimin et al., 2014) using the Burrows-Wheeler Aligner MEM algorithm with default parameters (Li, 2013). Variants were called using the SAMtools and BCFtools pipeline with default parameters (Li, 2011; Catchen et al., 2013). One previously genotyped population (No. 8) was included in this study, resulting in a total of 941 individuals from 24 populations in the final sequence dataset (Supplemental Table 1).

Several filtering steps were performed to minimize genotyping errors: SNPs located in repetitive regions (reference to *P. taeda* genome v.1.01) and with mapping quality <40 were removed; genotypes with genotype quality <20 or read depth <5 were masked as missing; loci with a missing rate of >30%, minor allele frequency of <5%, or heterozygosity of >70% or that were not biallelic were also removed.

Genetic diversity and population structure

The presence of related individuals, if undetected, would inflate population structure. To remove highly related samples, we examined relatedness among individuals in each population following the procedure of Hall et al. (2020). To obtain an overview of the spatial pattern of diversity we examined population structure using the R implementation of TESS3 (Caye et al., 2016) with the assumptions of one to five ancestral populations (*K*) and each *K* replicated 20 times. To avoid overestimating the number of potential clusters caused by the presence of IBD, which is often found in continuous populations, we used *conStruct* v.1.03 (Bradburd et al., 2018) to identify structure in a spatially aware context. We tested both the spatial and the non-spatial models with *K* values from 1 to 5 and 50 000 MCMC iterations for each test and with 5000 iterations and 10 replicates for model cross-validation.

To examine whether the individual seedling genotypes could be classified into their respective populations and whether the genetic variation could be attributed to geographic distance, we performed a PCA on the genetic covariances with EIGENSOFT v.6.1.4 (Patterson et al., 2006; Price et al., 2006). Genetic differentiation among populations was determined using pairwise F_{ST} (Weir and Cockerham, 1984) in Arlequin 3.5 (Excoffier and Lischer, 2010), where the statistical significance was assessed by 1023 permutations and a significance level of 0.05.

We estimated the nucleotide diversity at four-fold degenerate, synonymous sites (π_4) and zero-fold, non-synonymous sites (π_0) using the general feature format (gff) annotation file of *P. taeda*. We calculated observed (H_o) and expected (H_e) heterozygosity and fixation index F_{IS} in each population and overall. Pairwise nucleotide diversity at all sites (π) and at zero-fold (π_0) and four-fold (π_4) degenerate coding sites were computed using VCFtools (https://vcftools.github.io/index.html).

Genotype and environment association

As an initial screening of putative genotype–environment associations, we calculated the correlations between population-specific allele frequencies and their latitude, longitude, and GDD5 to identify possible allele frequency clines. We also compared the observed allele correlations among the three variables to establish whether there was an overlap between correlations.

To identify loci that are more or less differentiated than the average loci, we performed an F_{ST} outlier test of the SNPs using BayeScan (de Villemereuil et al., 2014; Foll and Gaggiotti, 2008). We also used TESS3 for F_{ST}-outlier loci detection. This method examines allele frequency changes while taking geographical constraints into account. We further analyzed the BayeScan outliers using TESS3 to see whether they resulted from shared ancestral components or possibly similar or linked selection and whether those components could be attributed to specific geographic origins.

To evaluate the impact of environmental factors on the differentiation at all loci and among outliers, we performed RDA over population allele frequencies (as the dependent matrix) and environmental parameters (independent matrix), a method that has been shown to be robust in comparative studies for detecting genotype by environment associations (Forester et al., 2018). We included two independent matrices in RDA,

an environmental matrix and a geo-distance matrix in the form of PCNM. We performed forward selection on 68 environmental variables (Supplemental Table 2) and on 15 PCNM decomposed geographic distance variables. The number of variables was reduced with a stepwise model to the four most influential variables in each dataset. We also ran partial RDA models, conditional on the geographic distance and environmental distance, respectively, to assess the exclusive impacts of env- and geo-factors on population differentiation and whether we could observe outlier SNPs exclusively shaped by the environment. Evaluation of SNP significance in the RDA was based on *anova.cca* function in the "vegan" R package (Oksanen et al., 2019). Significance tests followed the method in Capblancq et al., (2018).

Association mapping with damage levels

To further examine genotype–phenotype associations, we applied more direct association mapping of genotypes to freeze-damage levels. The damage scores were normalized over replication and freezing time points with the quantile normalization procedure in the Bioconductor R package "preprocessCore" (Bolstad, 2020), which results in comparable phenotypes across replicates.

Association mapping with damage levels was performed using both the LMM (Zhou and Stephens, 2012) and the BSLMM (Zhou et al., 2013), implemented in GEMMA. Both models are expected to control for population structure and kinship. We used the centered genotype matrix (mean genotype = 0) for both models. Two datasets were tested, one set was the full 24 populations with 935 genotypes (six replicated samples were removed, related individuals were kept), and another was a reduced set, which excluded populations 10, 16, and 26 from NW Norway. To estimate a significant threshold, we ran a permutation test of the LMM where phenotypes were shuffled 1000 times for both datasets.

To estimate SNP effects, we used BSLMM, which estimates the genetic effect of each marker without assigning significance and is a type of modeling that has been used for genomic selection (Meuwissen et al., 2001). One of the advantages of the BSLMM is that, in addition to estimating the *PVE* (proportion of genetic effects contributing to the total phenotypic variance, "chip heritability"), we could also estimate the GVE_β (proportion of major genetic effects contributing to the genetic variation). To estimate how much the major SNP effects contribute to the total phenotypic variance (PVE_β), we multiplied equations 13 (*PVE*) and 14 (GVE_β) from Zhou et al. (2013). The BSLMM model was run with 10^5 burn-in steps and 10^6 to 10^7 iterations for multiple runs to compare results, thinning sets to 10 and allowing for up to 500 sparse effects.

ACCESSION NUMBERS

Raw GBS library data reads have been deposited to NCBI under BioProject PRJNA687348 (https://www.ncbi.nlm.nih.gov/bioproject/PRJNA687348/). Final VCF files (https://doi.org/10.5878/8bg9-ah89 and https://doi.org/10.5878/rjzz-jk23), genotype population and phenotype information (https://doi.org/10.5878/0msc-3v36), population coordinates and environmental variables (https://doi.org/10.5878/45yn-ag55), and freeze test protocol and results (https://doi.org/10.5878/7pt2-ja36) are freely available from the Swedish National Data Service.

SUPPLEMENTAL INFORMATION

Supplemental information is available at *Plant Communications Online*.

FUNDING

Freezing tests were performed by Skogforsk and sponsored by NEFCO through the Programme for Environment and Climate Co-operation. This study was supported by grants from Formas, TC4F, Carl Tryggers Stiftelse, and Umeå Plant Science Center, Sweden.

AUTHOR CONTRIBUTIONS

U.W., J.K., D.H., and X.R.W. designed the study; U.W. and J.K. conducted the freezing tests; J.O., D.H., W.Z., and X.R.W. performed the lab work; D.H., W.Z., and J.O. analyzed the data; D.H., J.O., and X.R.W. wrote the manuscript with contributions from all authors.

ACKNOWLEDGMENTS

We thank Margareta Edvardsson with the staff at the nursery, Jörgen Hajek and Torgny Persson for advice concerning the freezing test, and Natalia Demidova at NRIF, Seppo Ruotsalainen at LUKE, and Arne Steffenrem and Øyvind Meland Edvardsen at NIBIO for help with Russian, Finnish, and Norwegian seed-lots, respectively. Genomic data processing and analyses were performed using resources provided by the Swedish National Infrastructure for Computing, through the High Performance Computing Center North (HPC2N), Umeå University. We thank Professor Barbara Giles for valuable comments on the manuscript and linguistic editing. We would also like to thank three anonymous reviewers and the handling editor for constructive comments and suggestions that greatly improved the manuscript. No conflict of interest declared.

Received: April 13, 2020
Revised: December 12, 2020
Accepted: December 25, 2020
Published: December 29, 2020

REFERENCES

Alberto, F.J., Aitken, S.N., Alía, R., González-Martínez, S.C., Hänninen, H., Kremer, A., Lefèvre, F., Lenormand, T., Yeaman, S., Whetten, R., et al. (2013). Potential for evolutionary responses to climate change – evidence from tree populations. Glob. Change Biol. **19**:1645–1661.

Andersson, B. (1992). Autumn frost hardiness of *Pinus sylvestris* offspring from seed orchard grafts of different ages. Scand. J. For. Res. **7**:367–375.

Andersson, B., and Fedorkov, A. (2004). Longitudinal differences in Scots pine frost hardiness. Silvae Genet. **53**:76–80.

Barton, N., Hermisson, J., and Nordborg, M. (2019). Why structure matters. eLife **8**:e45380.

Bates, D., Machler, M., Bolker, B.M., and Walker, S.C. (2015). Fitting linear mixed-effects models using lme4. J. Stat. Softw. **67**:1–48.

Bennett, K.D., Tzedakis, P.C., and Willis, K.J. (1991). Quaternary refugia of north European trees. J. Biogeogr. **18**:103–115.

Bennie, J., Kubin, E., Wiltshire, A., Huntley, B., and Baxter, R. (2010). Predicting spatial and temporal patterns of bud-burst and spring frost risk in north-west Europe: the implications of local adaptation to climate. Glob. Change Biol. **16**:1503–1514.

Berlin, M., Jansson, G., Danell, Ö., Andersson, B., Elfving, B., and Ericsson, T. (2009). Economic weight of tree survival relative to volume production in tree breeding: a case study with *Pinus sylvestris* in northern Sweden. Scand. J. For. Res. **24**:288–297.

Bolger, A.M., Lohse, M., and Usadel, B. (2014). Trimmomatic: a flexible trimmer for Illumina sequence data. Bioinformatics **30**:2114–2120.

Bolstad, B. (2020). preprocessCore: a collection of pre-processing functions. R package version 1.50.0. https://github.com/bmbolstad/preprocessCore.

Bradburd, G.S., Coop, G.M., and Ralph, P.L. (2018). Inferring continuous and discrete population genetic structure across space. Genetics **210**:33–52.

Capblancq, T., Luu, K., Blum, M.G.B., and Bazin, E. (2018). Evaluation of redundancy analysis to identify signatures of local adaptation. Mol. Ecol. Resour. **18**:1223–1233.

Catchen, J., Hohenlohe, P.A., Bassham, S., Amores, A., and Cresko, W.A. (2013). Stacks: an analysis tool set for population genomics. Mol. Ecol. **22**:3124–3140.

Catchen, J.M., Amores, A., Hohenlohe, P., Cresko, W., and Postlethwait, J.H. (2011). Stacks: building and genotyping loci de novo from short-read sequences. G3 **1**:171–182.

Caye, K., Deist, T.M., Martins, H., Michel, O., and François, O. (2016). TESS3: fast inference of spatial population structure and genome scans for selection. Mol. Ecol. Resour. **16**:540–548.

Cheddadi, R., Vendramin, G.G., Litt, T., François, L., Kageyama, M., Lorentz, S., Laurent, J.M., de Beaulieu, J.L., Sadori, L., Jost, A., et al. (2006). Imprints of glacial refugia in the modern genetic diversity of *Pinus sylvestris*. Glob. Ecol. Biogeogr. **15**:271–282.

Chen, J., Li, L., Milesi, P., Jansson, G., Berlin, M., Karlsson, B., Aleksic, J., Vendramin, G.G., and Lascoux, M. (2019). Genomic data provide new insights on the demographic history and the extent of recent material transfers in Norway spruce. Evol. Appl. **12**:1539–1551.

Csilléry, K., Rodríguez-Verdugo, A., Rellstab, C., and Guillaume, F. (2018). Detecting the genomic signal of polygenic adaptation and the role of epistasis in evolution. Mol. Ecol. **27**:606–612.

de Villemereuil, P., Frichot, É., Bazin, É., François, O., and Gaggiotti, O.E. (2014). Genome scan methods against more complex models: when and how much should we trust them? Mol. Ecol. **23**:2006–2019.

Dering, M., Kosiński, P., Wyka, T.P., Pers–Kamczyc, E., Boratyński, A., Boratyńska, K., Reich, P.B., Romo, A., Zadworny, M., Żytkowiak, R., et al. (2017). Tertiary remnants and Holocene colonizers: genetic structure and phylogeography of Scots pine reveal higher genetic diversity in young boreal than in relict Mediterranean populations and a dual colonization of Fennoscandia. Divers. Distributions **23**:540–555.

Dvornyk, V., Sirviö, A., Mikkonen, M., and Savolainen, O. (2002). Low nucleotide diversity at the pal1 locus in the widely distributed *Pinus sylvestris*. Mol. Biol. Evol. **19**:179–188.

Excoffier, L., and Lischer, H.E.L. (2010). Arlequin suite ver 3.5: a new series of programs to perform population genetics analyses under Linux and Windows. Mol. Ecol. Resour. **10**:564–567.

Foll, M., and Gaggiotti, O. (2008). A genome-scan method to identify selected loci appropriate for both dominant and codominant markers: a Bayesian perspective. Genetics **180**:977–993.

Forester, B.R., Lasky, J.R., Wagner, H.H., and Urban, D.L. (2018). Comparing methods for detecting multilocus adaptation with multivariate genotype–environment associations. Mol. Ecol. **27**:2215–2233.

Gienapp, P. (2020). Opinion: is gene mapping in wild populations useful for understanding and predicting adaptation to global change? Glob. Change Biol. https://doi.org/10.1111/gcb.15058.

Hall, D., Hallingbäck, H.R., and Wu, H.X. (2016). Estimation of number and size of QTL effects in forest tree traits. Tree Genet. Genomes **12**:110.

Hall, D., Luquez, V., Garcia, V.M., St Onge, K.R., Jansson, S., and Ingvarsson, P.K. (2007). Adaptive population differentiation in phenology across a latitudinal gradient in European Aspen (*Populus tremula*, L.): a comparison of neutral markers, candidate genes and phenotypic traits. Evolution **61**:2849–2860.

Hall, D., Zhao, W., Wennström, U., Andersson Gull, B., and Wang, X.R. (2020). Parentage and relatedness reconstruction in *Pinus sylvestris* using genotyping-by-sequencing. Heredity **124**:633–646.

Hurme, P., Repo, T., Savolainen, O., and Pääkkönen, T. (1997). Climatic adaptation of bud set and frost hardiness in Scots pine (*Pinus sylvestris*). Can. J. For. Res. **27**:716–723.

Jankowski, A., Wyka, T.P., Żytkowiak, R., Nihlgård, B., Reich, P.B., and Oleksyn, J. (2017). Cold adaptation drives variability in needle structure and anatomy in *Pinus sylvestris* L. along a 1,900 km temperate–boreal transect. Funct. Eco **31**:2212–2223.

Johnsen, A., Fidler, A.E., Kuhn, S., Carter, K.L., Hoffmann, A., Barr, I.R., Biard, C., Charmantier, A., Eens, M., Korsten, P., et al. (2007). Avian Clock gene polymorphism: evidence for a latitudinal cline in allele frequencies. Mol. Ecol. **16**:4867–4880.

Kullman, L. (2008). Early postglacial appearance of tree species in northern Scandinavia: review and perspective. Quat. Sci. Rev. **27**:2467–2472.

Latta, R.G. (1998). Differentiation of allelic frequencies at quantitative trait loci affecting locally adaptive traits. Am. Nat. **151**:283–292.

Le Corre, V., and Kremer, A. (2003). Genetic variability at neutral markers, quantitative trait loci and trait in a subdivided population under selection. Genetics **164**:1205–1219.

Leinonen, T., McCairns, R.J.S., O'Hara, R.B., and Merila, J. (2013). Q_{ST}-F_{ST} comparisons: evolutionary and ecological insights from genomic heterogeneity. Nat. Rev. Genet. **14**:179–190.

Li, H. (2011). A statistical framework for SNP calling, mutation discovery, association mapping and population genetical parameter estimation from sequencing data. Bioinformatics **27**:2987–2993.

Li, H. (2013). Aligning sequence reads, clone sequences and assembly contigs with BWA-MEM. arXiv, arXiv:1303.3997.

Li, L. (2020). Past Demography and Local Adaptation in Forest Trees : Insights from Natural Populations and Breeding Programs of Norway Spruce, PhD thesis (Uppsala, Sweden: Acta Universitatis Upsaliensis), p. 60, ISBN 978-91-513-0865-4.

Ma, X.-F., Hall, D., Onge, K.R.S., Jansson, S., and Ingvarsson, P.K. (2010). Genetic differentiation, clinal Variation and phenotypic associations with growth cessation across the *Populus tremula* photoperiodic pathway. Genetics **186**:1033–1044.

Meuwissen, T.H.E., Hayes, B.J., and Goddard, M.E. (2001). Prediction of total genetic value using genome-wide dense marker maps. Genetics **157**:1819–1829.

Milesi, P., Berlin, M., Chen, J., Orsucci, M., Li, L.L., Jansson, G., Karlsson, B., and Lascoux, M. (2019). Assessing the potential for assisted gene flow using past introduction of Norway spruce in southern Sweden: local adaptation and genetic basis of quantitative traits in trees. Evol. Appl. **12**:1946–1959.

Nadeau, S., Meirmans, P.G., Aitken, S.N., Ritland, K., and Isabel, N. (2016). The challenge of separating signatures of local adaptation from those of isolation by distance and colonization history: the case of two white pines. Ecol. Evol. **6**:8649–8664.

Naydenov, K., Senneville, S., Beaulieu, J., Tremblay, F., and Bousquet, J. (2007). Glacial vicariance in Eurasia: mitochondrial DNA evidence from Scots pine for a complex heritage involving genetically distinct refugia at mid-northern latitudes and in Asia Minor. BMC Evol. Biol. **7**:233.

Neale, D.B., Wegrzyn, J.L., Stevens, K.A., Zimin, A.V., Puiu, D., Crepeau, M.W., Cardeno, C., Koriabine, M., Holtz-Morris, A.E., Liechty, J.D., et al. (2014). Decoding the massive genome of loblolly pine using haploid DNA and novel assembly strategies. Genome Biol. **15**:R59.

Oakeshott, J.G., Gibson, J.B., Anderson, P.R., Knibb, W.R., Anderson, D.G., and Chambers, G.K. (1982). Alcohol dehydrogenase and glycerol-3-phosphate dehydrogenase clines in *Drosphila melanogaster* on different continents. Evolution **36**:86–96.

Oksanen, J., Blanchet, F.G., Friendly, M., Kindt, R., Legendre, P., McGlinn, D., Minchin, P.R., O'Hara, R.B., Simpson, G.L., Solymos, P., et al. (2019). vegan: community ecology package. R package version 2.5-6. https://CRAN.R-project.org/package=vegan.

Pan, J., Wang, B.S., Pei, Z.Y., Zhao, W., Gao, J., Mao, J.F., and Wang, X.R. (2015). Optimization of the genotyping-by-sequencing strategy for population genomic analysis in conifers. Mol. Ecol. Resour. **15**:711–722.

Parducci, L., Jørgensen, T., Tollefsrud, M.M., Elverland, E., Alm, T., Fontana, S.L., Bennett, K.D., Haile, J., Matetovici, I., Suyama, Y., et al. (2012). Glacial survival of boreal trees in northern Scandinavia. Science **335**:1083–1086.

Patterson, N., Price, A.L., and Reich, D. (2006). Population structure and eigenanalysis. PLoS Genet **2**. https://doi.org/10.1371/journal.pgen.0020190.

Persson, T., Andersson, B., and Ericsson, T. (2010). Relationship between autumn cold hardiness and field performance in northern *Pinus sylvestris*. Silva Fennica **44**:255–266.

Price, A.L., Patterson, N.J., Plenge, R.M., Weinblatt, M.E., Shadick, N.A., and Reich, D. (2006). Principal components analysis corrects for stratification in genome-wide association studies. Nat. Genet. **38**:904–909.

Pyhäjärvi, T., García-Gil, M.R., Knürr, T., Mikkonen, M., Wachowiak, W., and Savolainen, O. (2007). Demographic history has influenced nucleotide diversity in European *Pinus sylvestris* populations. Genetics **177**:1713–1724.

Pyhäjärvi, T., Kujala, S.T., and Savolainen, O. (2020). 275 years of forestry meets genomics in *Pinus sylvestris*. Evol. Appl. **13**:11–30.

Pyhäjärvi, T., Salmela, M.J., and Savolainen, O. (2008). Colonization routes of *Pinus sylvestris* inferred from distribution of mitochondrial DNA variation. Tree Genet. Genomes **4**:247–254.

Rehfeldt, G.E. (1989). Ecological adaptations in Douglas-Fir (*Pseudotsuga menziesii* var. *glauca*): a Synthesis. Ecol. Manage **28**:203–215.

Rehfeldt, G.E., Tchebakova, N.M., Parfenova, Y.I., Wykoff, W.R., Kuzmina, N.A., and Milyutin, L.I. (2002). Intraspecific responses to climate in *Pinus sylvestris*. Glob. Change Biol. **8**:912–929.

San-Miguel-Ayanz, J., De Rigo, D., Caudullo, G., Durrant, T.H., and Mauri, A. (2016). European Atlas of Forest Tree Species (Luxembourg: Publications Office of the European Union).

Savolainen, O., Bokma, F., Garcıa-Gil, R., Komulainen, P., and Repo, T. (2004). Genetic variation in cessation of growth and frost hardiness and consequences for adaptation of *Pinus sylvestris* to climatic changes. Ecol. Manage **197**:79–89.

Savolainen, O., Pyhäjärvi, T., and Knürr, T. (2007). Gene flow and local adaptation in trees. Annu. Rev. Ecol. Evol. Syst. **38**:595–619.

Semerikov, V.L., Semerikova, S.A., Putintseva, Y.A., Tarakanov, V.V., Tikhonova, I.V., Vidyakin, A.I., Oreshkova, N.V., and Krutovsky, K.V. (2018). Colonization history of Scots pine in Eastern Europe and North Asia based on mitochondrial DNA variation. Tree Genet. Genomes **14**:8.

Shutyaev, A., and Giertych, M. (1998). Height growth variation in a comprehensive Eurasian provenance experiment of (*Pinus sylvestris* L.). Silvae Genet. **46**:332–348.

Spitze, K. (1993). Population structure in *Daphnia obtusa*: quantitative genetic and allozymic variation. Genetics **135**:367–374.

Stinchcombe, J.R., Weinig, C., Ungerer, M., Olsen, K.M., Mays, C., Halldorsdottir, S.S., Purugganan, M.D., and Schmitt, J. (2004). A latitudinal cline in flowering time in *Arabidopsis thaliana* modulated by the flowering time gene *FRIGIDA*. Proc. Natl. Acad. Sci. U S A **101**:4712–4717.

Strømme, C.B., Schmidt, E., Olsen, J.E., and Nybakken, L. (2019). Climatic effects on bud break and frost tolerance in the northernmost populations of Beech (*Fagus sylvatica*) in Europe. Trees **33**:79–89.

Sullivan, A.R. (2020). A Forest Dark: an Evolutionary History of Norway Spruce, PhD thesis (Umeå, Sweden: Umeå University), ISBN 978-91-7855-211-5.

Tollefsrud, M.M., Sønstebø, J.H., Brochmann, C., Johnsen, O., Skrøppa, T., and Vendramin, G.G. (2009). Combined analysis of nuclear and mitochondrial markers provide new insight into the genetic structure of North European *Picea abies*. Heredity **102**:549–562.

Tóth, E.G., Köbölkuti, Z.A., Pedryc, A., and Höhn, M. (2017). Evolutionary history and phylogeography of Scots pine (*Pinus sylvestris* L.) in Europe based on molecular markers. J. For. Res. 1–15.

Tsuda, Y., Chen, J., Stocks, M., Källman, T., Sønstebø, J.H., Parducci, L., Semerikov, V., Sperisen, C., Politov, D., Ronkainen, T., et al. (2016). The extent and meaning of hybridization and introgression between Siberian spruce (*Picea obovata*) and Norway spruce (*Picea abies*): cryptic refugia as stepping stones to the west? Mol. Ecol. **25**:2773–2789.

Tyrmi, J.S., Vuosku, J., Acosta, J.J., Li, Z., Sterck, L., Cervera, M.T., Savolainen, O., and Pyhajarvi, T. (2020). Genomics of clinal local adaptation in *Pinus sylvestris* under continuous environmental and spatial genetic setting. G3 (Bethesda) **10**:2683–2696.

Wang, I.J., and Bradburd, G.S. (2014). Isolation by environment. Mol. Ecol. **23**:5649–5662.

Weir, B.S., and Cockerham, C.C. (1984). Estimating F-statistics for the analysis of population structure. Evolution **38**:1358–1370.

Whitlock, M.C. (1999). Neutral additive genetic variance in a metapopulation. Gen. Res. **74**:215–221.

Wright, S. (1943). Isolation by distance. Genetics **28**:114–138.

Yeaman, S. (2015). Local adaptation by alleles of small effect. The Am. Naturalist **186**:S74–S89.

Zale, R., Huang, Y.T., Bigler, C., Wood, J.R., Dalén, L., Wang, X.R., Segerström, U., and Klaminder, J. (2018). Growth of plants on the Late Weichselian ice-sheet during Greenland interstadial-1? Quat. Sci Rev **185**:222–229.

Zhao, W., Sun, Y.-Q., Pan, J., Sullivan, A.R., Arnold, M.L., Mao, J.-F., and Wang, X.R. (2020). Effects of landscapes and range expansion on population structure and local adaptation. New Phytol. **228**:330–343.

Zhou, X., Carbonetto, P., and Stephens, M. (2013). Polygenic modeling with Bayesian sparse linear mixed models. PLoS Genet. **9**:e1003264.

Zhou, X., and Stephens, M. (2012). Genome-wide efficient mixed-model analysis for association studies. Nat. Genet. **44**:821–824.

Zimin, A., Stevens, K.A., Crepeau, M., Holtz-Morris, A., Koriabine, M., Marçais, G., Puiu, D., Roberts, M., Wegrzyn, J.L., de Jong, P.J., et al. (2014). Sequencing and assembly of the 22-Gb Loblolly pine genome. Genetics **196**:875–890.

Zimmer, K., and Sønstebø, J.H. (2018). A preliminary study on the genetic structure of Northern European *Pinus sylvestris* L. by means of neutral nuclear microsatellite markers. Scand. J. For. Res. **33**:6–13.

Plant Communications, Volume *2*

Supplemental Information

Divergent patterns between phenotypic and genetic variation in Scots pine

David Hall, Jenny Olsson, Wei Zhao, Johan Kroon, Ulfstand Wennström, and Xiao-Ru Wang

Divergent pattern between phenotypic and genetic variation in Scots pine

David Hall, Jenny Olsson, Wei Zhao, Johan Kroon, Ulfstand Wennström and Xiao-Ru Wang

Supporting information

Supplementary Methods

Sampling and freezing test

To get a comprehensive view of the hardiness variation and genetic diversity and possible genetic structure of the northwestern distribution range of Scots pine, we collected 54 populations ranging from Norway to western Russia, and covering latitudes 57.5°N - 69.1°N (Figure 1, Supplemental Table 1), of which 53 populations were freeze tested. For most stands, cones were collected by commercial forest companies in stands consisting of 100 – 1000 trees. Seeds were extracted from cones in bulk for each stand. Seeds were then randomly picked from these bulk collections to be used in the freezing test. However, cones from population 39 was collected around the forestry research station in Arkhangelsk, Russia and population 40 from a seed orchard based on local stand trees.

The 53 populations were randomly divided into five groups. Within a group, seeds were sown in seedling boxes, with seven by eleven pots. Based on earlier assessment of germination, one, two (most stands) or three seeds were sown per pot (see Table S1). Each population was randomly given a number 1-55 and divided in five groups (A-E) according to their number, 1-11, 12-22, etc. In replication one all populations were sown in numerical order from left to right. To avoid edge effects, populations within a box were moved one column to the right within box and replicate (population in column eleven was moved to column one). Each seedling's position in the freezer was mapped for calculation of edge effect (KantF). Seedlings were sown in unfertilized peat and grown in a dark greenhouse at +20°C for the first week. In the following weeks of 1-9, the seedlings had 20/4 hr light/dark condition at +20°C daytime and +15°C during night. From week 10 the nighttime was increased by 1 hr every week with +15°C daytime and +5°C during night to initiate bud dormancy. Before exposure to freezing temperatures the pots where controlled for number of seedlings. If more than one seed had germinated, only the most central seedling was kept. Seedling boxes were placed into a freezer chamber at ten different time points after the dormancy initiation treatment, for two hours at -10°C. First freezing was conducted at 39 days after dormancy initiation (DDI) and the final freezing at 70 DDI. The scoring of needle damage on each seedling after exposure to low temperatures was done at least 1 week after exposure to allow for discoloration to develop. Each seedling was only frozen once but each population was represented in each replication. The degree of needle damage was scored visually into seven classes: **0**, no needle discoloration; **1**, 1-20% of needles discolored; **2**, 20-40%; **3**, 40-60%; **4**, 60-80%; **5**, 80-99% and **6** when all needles are completely discolored. A low score indicates low needle damage and therefore higher tolerance to freezing temperature (Table S1). This testing protocol was established by the Forestry Research Institute of Sweden (Skogforsk) in Sävar (Andersson, 1992, Persson et al., 2010) as a standard method for monitoring the hardiness of Scots pine seed orchard crops for reforestation since the early 1980s. In total there were 18 replications, of which each population had up to 7 seedlings represented per replication, made over 10 freezing time points, of which 15 were visually scored by a single person. Needles were collected from seedlings for genotyping before they were subjected to freeze exposure.

Phenotypic analyses

Freezing damage was analyzed using a generalized linear mixed model (GLMM) with the 'glmer' function in the R-package 'lme4' (Bates et al., 2015). We assumed a Poisson

distributed response variable of damage categories as a function of the fixed effects of placement in the freezer (KantF), DDI, longitude and latitude at origin and their interactions. Individual plant was set as a random effect of the population within replication. Longitude, latitude and DDI was centered (subtracting the mean) and then scaled by dividing with their standard deviations, because these variables were on a different scale than the response variable and caused instability during model fitting. The average damage levels of the populations were calculated as the least square means (LSM) for each sampled population to remove some experimental effects. These LSMs were then used for regression analysis against latitude, longitude and growing degree days with a 5°C base (GDD5). We used linear regression with latitude and longitude while a logistic regression was used with GDD0 and GDD5 where the LSM values were first normalized to a range between 0 and 1. In addition, we performed simple kriging (i.e. we assume we know the mean of our sampled region) on the freezing damage LSM to extrapolate expected frost tolerance across the sampled space. The semivariogram was calculated and fitted to a Gaussian model in the R-package 'gstat' (Pebesma, 2004).

We also sampled the posterior distribution of freezing damage differentiation among populations to estimate Q_{ST} in this trait with a Markov chain Monte Carlo (MCMC) procedure. Q_{ST} is the quantitative trait equivalent to the fixation index (F_{ST}), which is a measure of population differentiation due to genetic structure, and the comparison between Q_{ST} and F_{ST} can thus indicate if a trait is under balancing selection, i.e. $Q_{ST} < F_{ST}$, or under divergent or local adaptation, i.e. $Q_{ST} > F_{ST}$ (Leinonen et al., 2013, Spitze, 1993, Whitlock, 1999). We utilized the MCMCglmm package in R (Hadfield, 2010) following the full model above but with population and individual plant as random effects. We used 100 000 iterations with a burnin of 50 000 and thinning of 10. Q_{ST} for freezing damage was calculated as follows:

$$Q_{ST} = \frac{\sigma_B^2}{\sigma_B^2 + 2h^2\sigma_W^2}$$

where σ_B^2 is the between population variance estimate and σ_W^2 is the within population variance estimate (Spitze, 1993, Whitlock, 1999). The σ_W^2 term is confounded and contains the total within population phenotypic variance. Due to lack of family structure across the samples it is difficult to estimate additive genetic variance, which in turn will result in an underestimated Q_{ST}-value. However, see the association mapping section under the genotype and environment association subheading for marker estimated heritability.

We estimated pairwise Q_{ST}-values and compare them to differences in environmental variables, latitude, longitude, and physical and environmental distance between populations. To calculate environmental distance we performed a principal component analysis of 68 environmental variables (Supplemental Table 2) and used the decomposition of variables to calculate the Euclidean distance $D_{i,j}$ between populations i and j in the multidimensional space:

$$D_{i,j} = \sqrt{\sum_{n=1}^{N}\left(PC_{n_j} - PC_{n_i}\right)^2}$$

Where N is the number of principal components that explain a majority of variance observed or where adding additional eigenvector provide little explanatory gain ("elbow" in the scree-plot). Pairwise Q_{ST} was calculated utilizing a generalized linear mixed model due to the categorical data structure of freeze damage assessment for each seedlot and replication.

GBS library preparations

We extracted DNA for seedlings from 23 populations (Supplemental Table 1) using EZNA SP Plant DNA Kit (Omega Bio-tek). GBS library was prepared using a *Pst*I high-fidelity restriction enzyme (New England Biolabs® Inc.), following the protocol of (Pan et al., 2015).

Briefly, 200 ng DNA from each seedling was individually digested and ligated to sequencing adapters (with individual barcode) simultaneously. This was carried out at 37 °C for 8 h followed by 65 °C for 30 min. Then, the digested and ligated DNA of 300 samples were pooled into each library, purified, and PCR-amplified. Fragment size of 350–450 bp was selected using an E-gel EX 2% agarose gel (Thermo Fisher Scientific) and purified. Paired-end sequencing (2 × 150 bp) was performed on Illumina HiSeq X Ten. In each library, we included a few samples as within and among library replicates.

Bioinformatics

Sequence read quality was assessed with FastQC (http://www.bioinformatics.babraham.ac.uk/projects/fastqc/). Adapter sequences and low quality bases (Phred quality <20) from the tail of each read were removed by using Trimmomatic (Bolger et al., 2014). Clean reads were cataloged by using the process_radtags module of Stacks v2.0 (Catchen et al., 2011) according to individual barcode. Reads shorter than 41 bases were discarded. Sequence reads were aligned to the *Pinus taeda* draft genome v1.01 (Neale et al., 2014, Zimin et al., 2014), by using the Burrows-Wheeler Aligner mem (BWA-MEM) algorithm with default parameters (Li, 2013). Variants were called using the SAMtools and BCFtools pipeline with default parameters (Catchen et al., 2013, Li, 2011). One previously genotyped population (Skillingaryd in Southern Sweden, population No. 8 with 48 individuals, Figure 1A) was included in this study, resulting in a total of 941 individuals from 24 populations in the final sequence dataset (Supplemental Table 4).

Several filtering steps were performed to minimize genotyping errors: SNPs located in repetitive regions (reference to *P. taeda* genome v1.01) and with mapping quality (MQ) <40, were removed; genotypes with genotype quality (GQ) <20 or read depth (DP) <5 were masked as missing; loci with a missing rate of >30%, minor allele frequency (MAF) <5%, heterozygosity >70% or not biallelic were also removed.

Genetic diversity and population structure

We first examined relatedness among individuals in each population following the procedure of (Hall et al., 2020) to remove highly related samples. The presence of related individuals, if undetected, would inflate population structure. Relatedness between samples was based on the estimator proposed by Ritland (1996), a Method-of-moments estimator (MME) present in the R-package ‘related’(Pew et al., 2015) which in turn is an R implementation of the software ‘COANCESTRY’ (Wang, 2011). It requires a large population allele frequency reference which is based on a representative set of individuals. We did not know the relatedness among samples a priori, but we assumed that related individuals would be among different populations and not affect the overall allele frequency estimates, and used all samples as the reference set. Related samples were removed iteratively. First we removed the sample that had the largest number of pairwise comparisons among samples with elevated relatedness, above cousin level 0.125. Then the next and so forth until no sample had an elevated relatedness to any other sample.

We estimated the nucleotide diversity at synonymous sites (π_4) and non-synonymous sites (π_0). Using *Pinus taeda* genome as reference for mapping, we could annotate the SNPs based on the General Feature Format (gff) annotation file of *P. taeda*. A position of a codon is said to be an n-fold degenerate site if only n of four possible nucleotides (A, C, G, and T) at this position specify the same amino acid. A nucleotide substitution at a fourfold degenerate site is referred to as a synonymous nucleotide substitution, and 0-fold degenerate site is nonsynonymous site. The observed (H_o) and expected (H_e) heterozygosity and fixation index F_{IS} in each populations and overall, and pairwise nucleotide diversity at all sites (π) and at 0-

fold (π_0) and 4-fold (π_4) degenerate sites were computed using VCFtools (https://vcftools.github.io/index.html).

To obtain an overview of the spatial pattern of diversity, we examined population structure using the R-implementation of TESS3 (Caye et al., 2016). TESS3 assumes that the genotypes are sampled from an admixed populations originating from *K* ancestral clusters, like other softwares, such as *fastStructure* (Raj et al., 2014), that estimates ancestral components. The likelihood method used then assumes that the probability of sampling from *K* number ancestral genotype pools is equal to the admixture coefficients. The probabilities are geographically constrained, meaning that neighboring samples are more likely to share ancestral genotypes. We ran the TESS3 algorithm with the assumptions of 1 to 5 ancestral populations (*K*) and each *K* was replicated 20 times. To avoid overestimating the number of potential clusters caused by the presence of isolation by distance (IBD), as is often found in continuous populations, we used *conStruct* v.1.03 (Bradburd et al., 2018) to identify structure in a spatially aware context. We tested both the spatial and non-spatial models with *K* values from 1 to 5 and 50,000 MCMC iterations for each test. Model cross-validation was run with 5000 iterations and 10 replicates to test which model best explains the distribution of genetic variation. For each best fit *K*, i.e. the number of ancestral layers that explains most of the variation, we conducted three independent runs to evaluate the convergence.

Genetic differentiation among populations was determined using pairwise F_{ST} (Weir and Cockerham, 1984) in Arlequin 3.5 (Excoffier and Lischer, 2010), where the statistical significance was assessed by 1023 permutations, at significance level of 0.05. To examine if the individual seedlings genotypes could be categorized into their respective populations and if the genetic variation can be attributed to geographic distance, we performed a principal component analysis (PCA) on the genetic covariances with EIGENSOFT v 6.1.4 (Patterson et al., 2006, Price et al., 2006).

Genotype and environment association

To examine possible allele frequency clines, as an initial screening of putative genotype-environment association, we calculated the association of allele frequencies in populations with latitude, longitude and GDD5. We also compared the observed allele correlations (Spearman's ρ) among the three variables to establish if there was an overlap between correlations.

To identify loci that are more or less differentiated than the average loci, we performed a F_{ST} outlier test of the SNPs using BayeScan (Foll and Gaggiotti, 2008). This method has been shown to have elevated type I error rates under IBD (de Villemereuil et al., 2014). However, we know from previous studies that the expected differentiation among loci is low (Hall et al., 2020:Tyrmi, 2020 #1139), and we are interested in examining putative patterns of all loci with elevated differentiation. We also used TESS3 (Caye et al., 2016) for F_{ST}-outlier loci detection, but in contrast to BayeScan, TESS3 is based on the estimated ancestral allele frequencies which in turn are geographically constrained. In addition, we further analyzed those highly differentiated loci identified by BayeScan using TESS3. We did this to see if they were a result of any shared ancestral component and if those components could be attributed to a specific geographic origin (Caye et al., 2016).

We considered whether environmental factors could have shaped the patterns of differentiation in all loci and among outliers by performing a redundancy analysis (RDA) over population allele frequencies and environmental parameters, a method that has been shown to be robust in comparative studies in detecting genotype by environment associations (Forester et al., 2018). We ran one full RDA based on two predictive matrices, environment and geographic distance in the form of Principal coordinates of neighbor matrices (PCNM). PCNMs considers the spatial input variables (longitude and latitude in this study) and determine the distance between sites and neighboring sites in particular. These distances are

then decomposed into a new set of independent spatial variables. The PCNM removes spatial autocorrelations (Dray et al., 2006). However, this requires assumptions about the connectedness between samplings sites which could inflate the explanatory power (Gilbert and Bennett, 2010). The two matrices are evaluated by separate RDAs on each matrix. One RDA with 68 environmental variable (Supplemental Table 2), and one with the 15 decomposed geographic distance variables. We applied a forward stepwise selection with the function *ordistep* in the vegan –R-package for parameters on the two models separately. The function employs permutation tests to only keep those that had the most influence. The algorithm perform a stepwise addition of variables that significantly contribute to the model fit. For each step the remaining variables are evaluated to find the variable that would explain most of the remaining variance and give the lowest AIC-value. This forward stepping procedure is performed until there is no more significant improvement of the model, making the best fit model with the fewest possible variables. We also made sure that none of the remaining variables from the forward selection shared high levels of information ($|R| < 0.75$) (Oksanen et al., 2019). The full model with the two matrices are then partitioned to evaluate the two predictors' relative importance and confounding using the function *varpart* in the 'vegan' R-package. We also ran a partial RDA, conditional on the geographic distance, to assess whether we could observe outlier SNPs exclusively shaped by the environment.

Evaluation of SNP-significance in the RDA was based on the number of significant constrained axis (K) evaluated with the *anova.cca* function in the 'vegan' R-package (Oksanen et al., 2019). We then performed significant tests following the method in (Capblancq et al., 2018). In short, for each loci the Mahalanobis distance is estimated within the variance covariance matrix of K number dimensions through the *covRob* function of the 'robust' R-package (Wang et al., 2020). Loci with an extreme Mahalanobis distance are considered significant. The Mahalanobis distances has a Chi-square distribution with K degrees of freedom and distances are considered significant at q-values ≤ 0.05 after adjusting p-values for a false discovery rate with the 'qvalue' R-package (Storey et al., 2020).

Association mapping with damage levels

To further examine genotype-phenotype and genotype-environment associations we applied more direct association mapping of genotypes to freeze damage levels and to population environmental variables. To accommodate a more uniform dataset in association mapping on the genotyped and phenotyped seedlings we had to normalize the data over replication and freezing time points. This was necessary because each seedling (genotype) was only phenotyped once and dormancy progression will strongly influence their resistance to frost. We used quantile normalization procedure from the Bioconductor R-package 'preprocessCore' (Bolstad, 2020), which results in comparable phenotypes across replicates. However, because the underlying data has a Poisson distribution the result from the quantile normalization is pseudo-continuous with a non-normal distribution.

Association mapping with damage levels were performed using both the univariate linear mixed model (LMM, Zhou and Stephens, 2012) and Bayesian sparse linear mixed model (BSLMM, Zhou et al., 2013) implemented in GEMMA. Both these models are expected to control for population structure and kinship. We used the centered genotype matrix (mean genotype = 0) for both models. Two datasets were used with both models. Data set one was the full dataset with 935 genotypes, (6 genotype replicated samples were removed) which included related individuals, and a reduced set which excluded populations 10, 16 and 26 of north western Norway. To estimate a significant threshold we ran a permutation test of the LMM where phenotypes were shuffled 1000 times for both data sets. Minimum p-values for each permutation was stored and the lower 5% tail of the distribution considered a significant

threshold comparable to a 5% FDR. We also considered the lowest observed *p*-value of the permutation runs as a threshold for a highly significant effect.

To estimate SNP effects we used BSLMM, which estimates the genetic effect of each marker without assigning significance, and is a type of modelling that has been used for genomic selection (Meuwissen et al., 2001). One of the advantages of the BSLMM is that it is a mixed distribution model where in addition to estimate the *PVE* (proportion of genetic effects contributing the total phenotypic variance, "chip heritability"), we can also get an estimate of the GVE_β (proportion of major genetic effects contributing to the genetic variation) almost independently. To get an estimate of how much major SNP effects contributes to the total phenotypic variance, GVE_β, we multiplied equations 13 (*PVE*) and 14 (GVE_β) from Zhou et al. (2013) and thus get the following:

$$PVE_\beta(\tilde{\beta}, \boldsymbol{u}, \tau) = \frac{V(\boldsymbol{X}\tilde{\beta})}{V(\boldsymbol{X}\tilde{\beta} + \boldsymbol{u}) + \tau^{-1}}$$

where $\boldsymbol{u}$ is the polygenic component which captures the combined small effects of all markers, τ the error variance, $\tilde{\beta}$ the major SNP effects or "sparse effects" and $\boldsymbol{X}$ is the genotype matrix.

References

Andersson, B. (1992). Autumn frost hardiness of *Pinus sylvestris* offspring from seed orchard grafts of different ages. Scand J Forest Res **7**:367-375.

Bates, D., Machler, M., Bolker, B.M., and Walker, S.C. (2015). Fitting linear mixed-effects models using lme4. J Stat Softw **67**:1-48.

Bolger, A.M., Lohse, M., and Usadel, B. (2014). Trimmomatic: a flexible trimmer for Illumina sequence data. Bioinformatics **30**:2114-2120.

Bolstad, B. (2020). preprocessCore: A collection of pre-processing functions. *R package version 1.50.0*. https://github.com/bmbolstad/preprocessCore.

Bradburd, G.S., Coop, G.M., and Ralph, P.L. (2018). Inferring continuous and discrete population genetic structure across space. Genetics **210**:33-52.

Capblancq, T., Luu, K., Blum, M.G.B., and Bazin, E. (2018). Evaluation of redundancy analysis to identify signatures of local adaptation. Mol Ecol Resour **18**:1223-1233.

Catchen, J., Hohenlohe, P.A., Bassham, S., Amores, A., and Cresko, W.A. (2013). Stacks: an analysis tool set for population genomics. Mol Ecol **22**:3124-3140.

Catchen, J.M., Amores, A., Hohenlohe, P., Cresko, W., and Postlethwait, J.H. (2011). Stacks: Building and genotyping loci de novo from short-read sequences. G3 **1**:171-182.

Caye, K., Deist, T.M., Martins, H., Michel, O., and François, O. (2016). TESS3: fast inference of spatial population structure and genome scans for selection. Mol Ecol Resour **16**:540-548.

de Villemereuil, P., Frichot, É., Bazin, É., François, O., and Gaggiotti, O.E. (2014). Genome scan methods against more complex models: when and how much should we trust them? Mol Ecol **23**:2006-2019.

Dray, S., Legendre, P., and Peres-Neto, P.R. (2006). Spatial modelling: a comprehensive framework for principal coordinate analysis of neighbour matrices (PCNM). Ecological Modelling **196**:483-493.

Excoffier, L., and Lischer, H.E.L. (2010). Arlequin suite ver 3.5: a new series of programs to perform population genetics analyses under Linux and Windows. Mol Ecol Resour **10**:564-567.

Foll, M., and Gaggiotti, O. (2008). A genome-scan method to identify selected loci appropriate for both dominant and codominant markers: a Bayesian perspective. Genetics **180**:977-993.

Forester, B.R., Lasky, J.R., Wagner, H.H., and Urban, D.L. (2018). Comparing methods for detecting multilocus adaptation with multivariate genotype–environment associations. Mol Ecol **27**:2215-2233.

Gilbert, B., and Bennett, J.R. (2010). Partitioning variation in ecological communities: do the numbers add up? Journal of Applied Ecology **47**:1071-1082.

Hadfield, J.D. (2010). MCMC methods for multi-response generalized linear mixed models: The MCMCglmm R package. J Stat Softw **33**:1-22.

Hall, D., Zhao, W., Wennström, U., Andersson Gull, B., and Wang, X.-R. (2020). Parentage and relatedness reconstruction in *Pinus sylvestris* using genotyping-by-sequencing. Heredity:DOI: https://doi.org/10.1038/s41437-41020-40302-41433.

Leinonen, T., McCairns, R.J.S., O'Hara, R.B., and Merila, J. (2013). Q_{ST}-F_{ST} comparisons: evolutionary and ecological insights from genomic heterogeneity. Nat Rev Genet **14**:179-190.

Li, H. (2011). A statistical framework for SNP calling, mutation discovery, association mapping and population genetical parameter estimation from sequencing data. Bioinformatics **27**:2987-2993.

Li, H. (2013). Aligning sequence reads, clone sequences and assembly contigs with BWA-MEM. arXiv preprint arXiv:1303.3997.

Meuwissen, T.H.E., Hayes, B.J., and Goddard, M.E. (2001). Prediction of total genetic value using genome-wide dense marker maps. Genetics **157**:1819-1829.

Neale, D.B., Wegrzyn, J.L., Stevens, K.A., Zimin, A.V., Puiu, D., Crepeau, M.W., Cardeno, C., Koriabine, M., Holtz-Morris, A.E., Liechty, J.D., et al. (2014). Decoding the massive genome of loblolly pine using haploid DNA and novel assembly strategies. Genome Biol **15**:R59.

Oksanen, J., Blanchet, F.G., Friendly, M., Kindt, R., Legendre, P., McGlinn, D., Minchin, P.R., O'Hara, R.B., Simpson, G.L., Solymos, P., et al. (2019). vegan: Community ecology package. *R package version 2.5-6.* https://CRAN.R-project.org/package=vegan.

Pan, J., Wang, B.S., Pei, Z.Y., Zhao, W., Gao, J., Mao, J.F., and Wang, X.R. (2015). Optimization of the genotyping-by-sequencing strategy for population genomic analysis in conifers. Mol Ecol Resour **15**:711-722.

Patterson, N., Price, A.L., and Reich, D. (2006). Population structure and eigenanalysis. Plos Genet **2**.

Pebesma, E.J. (2004). Multivariable geostatistics in S: the gstat package. Computers & Geosciences **30**:683-691.

Persson, T., Andersson, B., and Ericsson, T. (2010). Relationship between autumn cold hardiness and field performance in northern *Pinus sylvestris*. Silva Fennica **44**:255-266.

Pew, J., Muir, P.H., Wang, J.L., and Frasier, T.R. (2015). related: an R package for analysing pairwise relatedness from codominant molecular markers. Mol Ecol Resour **15**:557-561.

Price, A.L., Patterson, N.J., Plenge, R.M., Weinblatt, M.E., Shadick, N.A., and Reich, D. (2006). Principal components analysis corrects for stratification in genome-wide association studies. Nat Genet **38**:904-909.

Raj, A., Stephens, M., and Pritchard, J.K. (2014). fastSTRUCTURE: Variational inference of population structure in large SNP data sets. Genetics **197**:573-589.

Ritland, K. (1996). Estimators for pairwise relatedness and individual inbreeding coefficients. Genetical Research **67**:175-185.

Spitze, K. (1993). Population structure in *Daphnia obtusa*: quantitative genetic and allozymic variation. Genetics **135**:367-374.

Storey, J.D., Bass, A.J., Dabney, A., Robinson, D., and Warnes, G. (2020). qvalue: Q-value estimation for false discovery rate control. *R package version 2.20.0*. http://github.com/jdstorey/qvalue.

Wang, J. (2011). COANCESTRY: a program for simulating, estimating and analysing relatedness and inbreeding coefficients. Mol Ecol Resour **11**:141-145.

Wang, J., Zamar, R., Marazzi, A., Yohai, V., Salibian-Barrera, M., Maronna, R., Zivot, E., Rocke, D., Martin, D., Maechler, M., et al. (2020). robust: Robust Library. *R Package Version 0.5-00.* https://CRAN.R-project.org/package=robust.

Weir, B.S., and Cockerham, C.C. (1984). Estimating F-statistics for the analysis of population structure. Evolution **38**:1358-1370.

Whitlock, M.C. (1999). Neutral additive genetic variance in a metapopulation. Gen Res **74**:215-221.

Zhou, X., Carbonetto, P., and Stephens, M. (2013). Polygenic modeling with Bayesian sparse linear mixed models. Plos Genet **9**:e1003264.

Zhou, X., and Stephens, M. (2012). Genome-wide efficient mixed-model analysis for association studies. Nat Genet **44**:821-824.

Zhou, X., and Stephens, M. (2014). Efficient multivariate linear mixed model algorithms for genome-wide association studies. Nature Methods **11**:407-409.

Zimin, A., Stevens, K.A., Crepeau, M., Holtz-Morris, A., Koriabine, M., Marçais, G., Puiu, D., Roberts, M., Wegrzyn, J.L., de Jong, P.J., et al. (2014). Sequencing and assembly of the 22-Gb Loblolly pine genome. Genetics **196**:875-890.

Tables

Table S1. Population information collection year and seeds sown per pot. Hardiness determined by average damage on needles after freeze test. LSM: the model corrected hardiness. NP: No. seedlings subjected to freezing test. NG: No. seedlings genotyped.

Population ID	Name	Collected	Seeds/ pot	Country	Lat.	Long.	GDD5	LSM (Damage)	NP	NG
1	Molde, Gjemnes, Skodje	1998	2	Norway	62.80	7.50	10521	4.03	104	59
2	Vågå, Oppland	2006	2	Norway	61.86	9.05	8243	3.14	102	21
3	Hemne, Sør-Trøndelag	1999	2	Norway	63.30	9.12	10215	3.34	103	66
4	Ringerike, Buskerud	2004	2	Norway	60.28	9.95	12483	3.93	104	61
5	Åsnes, Hedmark	2002	2	Norway	60.60	12.08	12204	4.18	105	-
6	Trysil, Hedmark	2005	2	Norway	61.22	12.37	8490	3.29	101	-
7	Torsby 1	2016	2	Sweden	60.20	12.90	12879	3.87	105	-
8	Skillingaryd	1999	1	Sweden	57.42	14.02	12906	-	-	48
9	Hattkullen B	1995	3	Sweden	60.89	14.23	8910	3.27	105	-
10	Beiarn, Nordland	1988	3	Norway	67.00	14.24	8243	2.22	103	60
11	Laforsen-Kårböle	1998	2	Sweden	61.97	15.48	9053	3.06	103	-
12	Gunnilbo_Kulheden	1995	3	Sweden	59.90	15.80	12987	3.86	90	59
13	Hammarstrand	1995	3	Sweden	63.15	16.20	8850	3.41	102	20
14	Sollefteå kommun	1998	2	Sweden	63.17	17.01	8925	3.15	105	-
15	Skorped	2016	2	Sweden	63.40	17.90	8535	2.70	105	-
16	Kirkesmoen, Troms	2011	2	Norway	68.90	18.30	4476	0.72	104	65
17	Abborrträskliden	1998	2	Sweden	64.82	18.80	8168	2.13	99	-
18	Arvidsjaur	1998	2	Sweden	65.60	19.20	7403	1.81	97	-
19	Kullsjöliden	1992	2	Sweden	64.10	19.80	8423	1.98	94	-
20	Nyträsk	1998	2	Sweden	65.00	20.50	8085	2.13	105	-
21	Harads	1998	2	Sweden	66.20	21.00	7860	1.87	102	-
22	Uusikaupunki	2004	2	Finland	60.84	21.49	12663	3.96	60	50
23	Tärendö	1998	2	Sweden	67.10	22.70	5370	0.78	104	-
24	Kuttainen	2003	2	Sweden	68.20	22.80	4650	0.66	93	-
25	Almajärvi	1998	3	Sweden	68.00	23.20	4854	0.50	102	-
26	Alta, Stengelsen	2013	2	Norway	69.87	23.27	4704	0.78	105	60
27	Kaunisvaara	1998	3	Sweden	67.50	23.30	5208	0.72	105	-
28	Svanstein	2016	2	Sweden	66.60	23.80	7733	1.00	84	-
29	Kourevesi	1977	1	Finland	62.00	24.80	9165	3.63	78	-
30	Rovaniemen mlk	2002	2	Finland	66.90	25.40	7718	1.09	105	20
31	Äänekoski	1999	2	Finland	62.80	25.70	8798	2.79	99	20
33	Riistina	1985	1	Finland	61.50	27.40	9998	3.81	25	17
34	Pudasjärvi	2004	2	Finland	65.50	27.60	7905	1.41	102	19
35	Suomussalmi	2002	2	Finland	65.00	29.10	7988	1.20	104	-
36	Kerimäki	2007	2	Finland	61.90	29.40	9548	3.43	105	-
37	Kuhmo	1997	2	Finland	64.10	29.60	8430	2.38	104	66
38	Archangelsk stand 5	2015	2	Russia	62.10	40.60	9075	3.15	100	18
39	Archangelsk stand 13	2016	2	Russia	64.52	40.70	8333	2.44	70	57
40	Archangelsk seed orchard	2015	2	Russia	61.00	42.30	9285	3.23	103	24
41	Archangelsk stand 12	2016	2	Russia	62.00	44.70	8963	3.37	87	-
43	Archangelsk stand 10	2016	2	Russia	61.90	45.00	9060	2.84	98	-
44	Archangelsk stand 11	2016	2	Russia	61.90	45.00	9060	2.89	95	-
45	Archangelsk stand 8	2016	2	Russia	61.90	45.00	9060	2.69	105	-
46	Archangelsk stand 7	2016	2	Russia	61.70	45.50	9263	3.47	102	-
47	Archangelsk stand 3	2015	2	Russia	61.20	46.20	9135	3.07	103	20
48	Archangelsk stand 4	2015	2	Russia	61.20	46.20	9135	2.68	105	-
49	Archangelsk stand 6	2016	2	Russia	62.10	46.60	8603	3.10	104	-
50	Archangelsk stand 2	2014	2	Russia	61.20	48.50	9233	3.03	103	-
51	Udora, Komi	2000	2	Russia	64.30	49.20	7935	1.40	79	19
52	Megdurechensk, Komi	2000	2	Russia	63.10	50.80	8640	2.03	83	19
53	Mechura, Komi	2000	2	Russia	63.80	51.20	8205	2.20	95	-
54	Ust-Kulom, Komi	2000	2	Russia	61.50	54.00	9330	2.78	97	-
55	Pomozdino, Komi	2000	2	Russia	62.10	54.30	8933	2.54	78	64
56	Sosnogorsk, Komi	2000	2	Russia	63.68	54.76	8003	1.82	8	9
**Total**									5028	941

Table S2. The 68 climatic variables used for phenotype- and genotype-environment association analyses. Bold text indicates those kept by redundancy analysis (RDA).

**Variable**	**Information**	**Reference**
BIO1	Annual Mean Temperature	1
BIO2	Mean Diurnal Range (Mean of monthly (max temp - min temp))	1
BIO3	Isothermality (BIO2/BIO7) (×100)	1
BIO4	Temperature Seasonality (standard deviation ×100)	1
BIO5	Max Temperature of Warmest Month	1
BIO6	Min Temperature of Coldest Month	1
BIO7	Temperature Annual Range (BIO5-BIO6)	1
**BIO8**	**Mean Temperature of Wettest Quarter**	**1**
BIO9	Mean Temperature of Driest Quarter	1
BIO10	Mean Temperature of Warmest Quarter	1
BIO11	Mean Temperature of Coldest Quarter	1
BIO12	Annual Precipitation	1
BIO13	Precipitation of Wettest Month	1
BIO14	Precipitation of Driest Month	1
BIO15	Precipitation Seasonality (Coefficient of Variation)	1
BIO16	Precipitation of Wettest Quarter	1
BIO17	Precipitation of Driest Quarter	1
BIO18	Precipitation of Warmest Quarter	1
BIO19	Precipitation of Coldest Quarter	1
frs01	Frost day frequency in January	2
frs02	Frost day frequency in February	2
frs03	Frost day frequency in March	2
frs04	Frost day frequency in April	2
frs05	Frost day frequency in May	2
frs06	Frost day frequency in June	2
frs07	Frost day frequency in July	2
frs08	Frost day frequency in August	2
frs09	Frost day frequency in September	2
frs10	Frost day frequency in October	2
frs11	Frost day frequency in November	2
frs12	Frost day frequency in December	2
vap01	Vapor pressure in January	2
vap02	Vapor pressure in February	2
vap03	Vapor pressure in March	2
vap04	Vapor pressure in April	2
vap05	Vapor pressure in May	2
vap06	Vapor pressure in June	2
vap07	Vapor pressure in July	2
vap08	Vapor pressure in August	2
vap09	Vapor pressure in September	2

Table S2 contd.

vap10	Vapor pressure in October	2
vap11	Vapor pressure in November	2
vap12	Vapor pressure in December	2
wet01	Wet day frequency in January	2
wet02	Wet day frequency in February	2
wet03	Wet day frequency in March	2
wet04	Wet day frequency in April	2
wet05	Wet day frequency in May	2
wet06	Wet day frequency in June	2
wet07	Wet day frequency in July	2
wet08	Wet day frequency in August	2
wet09	Wet day frequency in September	2
wet10	Wet day frequency in October	2
**wet11**	**Wet day frequency in November**	**2**
wet12	Wet day frequency in December	2
embergerQ	Emberger's pluviothermic quotient: a metric that was designed to differentiate among Mediterranean type climates	3
**GDD0**	**Sum of mean monthly temperature for months with mean temperature greater than 0°C multiplied by number of days**	**3**
GDD5	Sum of mean monthly temperature for months with mean temperature greater than 5°C multiplied by number of days	3
**maxTempColdestMonth**	**Max. temp. of the coldest month**	**3**
sc	Soil organic carbon	4
SpH	Soil pH	4
gdd	Annual GDD with 5°C baseline	4
uvb1	Annual Mean UV-B	5
uvb2	Annual UV-B Seasonality (standard deviation)	5
uvb3	Mean UV-B of Highest Month	5
uvb4	Mean UV-B of Lowest Month	5
uvb5	Sum of Monthly Mean UV-B during Highest Quarter	5
uvb6	Sum of Monthly Mean UV-B during Lowest Quarter	5

References

1. Fick, S.E., and Hijmans, R.J. (2017). WorldClim 2: new 1-km spatial resolution climate surfaces for global land areas. International Journal of Climatology 37:4302-4315.
2. Mitchell, T.D., and Jones, P.D. (2005). An improved method of constructing a database of monthly climate observations and associated high-resolution grids. International Journal of Climatology 25:693-712.
3. Title, P.O., and Bemmels, J.B. (2018). ENVIREM: an expanded set of bioclimatic and topographic variables increases flexibility and improves performance of ecological niche modeling. Ecography 41:291-307.
4. SAGE. Atlas of the Biosphere--The Center for Sustainability and the Global Environment, N.I.F.E.S., University of Wisconsin-Madison.
5. Beckmann, M., Václavík, T., Manceur, A.M., Šprtová, L., von Wehrden, H., Welk, E., and Cord, A.F. (2014). glUV: a global UV-B radiation data set for macroecological studies. Methods in Ecology and Evolution 5:372-383.

Table S3. Summary of GBS results for the analyzed Scots pine seedlings. Numbers in parentheses are the ±1 standard error. Reads with a low coverage or depth (less than or equal to 5x) were discarded.

	**Mean**	**Median**
Reads per individual	2,355,433 (± 94,478)	1,630,707
Coverage (Mbp, >=5x)	2.53 (± 0.026)	2.45
Depth (>=5x)	87x (± 1.51)	32x
Mapping rate	96.36 % (± 0.153)	97.41 %

Table S4. Number of related (including replicates) and unrelated individuals in each population. Only unrelated individuals were used in the genetic analyses.

**Population ID**	**Country**	**Related**	**Unrelated**	**Total**
1	Norway	21	38	59
2	Norway	3	18	21
3	Norway	26	40	66
4	Norway	27	34	61
8	Sweden	2	46	48
10	Norway	11	49	60
12	Sweden	3	56	59
13	Sweden	1	19	20
16	Norway	3	62	65
22	Finland	17	33	50
26	Norway	1	59	60
30	Finland	0	20	20
31	Finland	4	16	20
33	Finland	7	10	17
34	Finland	0	19	19
37	Finland	2	64	66
38	Russia	3	15	18
39	Russia	45	12	57
40	Russia	6	18	24
47	Russia	0	20	20
51	Russia	0	19	19
52	Russia	1	18	19
55	Russia	11	53	64
56	Russia	1	8	9
**Total**		**195**	**746**	**941**

Table S5. Pairwise F_{ST} estimates between all genotyped populations. Darker shade of red indicate higher pairwise differentiation.

Pop	1	2	3	4	8	10	12	13	16	22	26	30	31	33	34	37	38	39	40	47	51	52	55
**56**	0.0190	0.0136	0.0192	0.0129	0.0135	0.0191	0.0161	0.0152	0.0149	0.0058	0.0087	0.0122	0.0117	0.0126	0.0102	0.0134	0.0018	0.0057	0.0053	0.0022	0.0036	0.0041	0.0027
**55**	0.0158	0.0076	0.0139	0.0082	0.0116	0.0145	0.0101	0.0038	0.0084	0.0068	-0.0021	0.0073	0.0077	0.0108	0.0075	0.0053	-0.0036	-0.0031	0.0028	-0.0009	-0.0003	0.0003	
**52**	0.0144	0.0141	0.0143	0.0072	0.0091	0.0140	0.0105	0.0125	0.0120	0.0042	0.0053	0.0093	0.0096	0.0094	0.0049	0.0070	0.0013	0.0022	0.0000	-0.0004	0.0001		
**51**	0.0132	0.0152	0.0123	0.0050	0.0076	0.0124	0.0105	0.0123	0.0114	0.0042	0.0059	0.0063	0.0112	0.0097	0.0025	0.0054	0.0030	0.0032	-0.0004	-0.0025			
**47**	0.0152	0.0072	0.0153	0.0110	0.0119	0.0155	0.0105	0.0035	0.0082	-0.0011	-0.0024	0.0076	0.0051	0.0067	0.0071	0.0062	-0.0087	-0.0060	-0.0009				
**40**	0.0098	0.0024	0.0085	0.0020	0.0049	0.0102	0.0040	-0.0031	0.0034	0.0022	-0.0072	0.0028	0.0019	0.0050	0.0022	0.0006	-0.0079	-0.0072					
**39**	0.0020	0.0130	-0.0002	-0.0083	-0.0041	-0.0012	-0.0011	0.0119	0.0075	-0.0028	0.0066	-0.0022	0.0032	0.0014	-0.0084	-0.0034	0.0072						
**38**	0.0050	0.0137	0.0028	-0.0065	-0.0033	0.0049	0.0023	0.0110	0.0105	-0.0030	0.0074	0.0007	0.0063	0.0014	-0.0034	-0.0007							
**37**	0.0091	0.0045	0.0087	0.0053	0.0069	0.0089	0.0056	0.0031	0.0048	-0.0042	-0.0034	0.0029	0.0031	0.0022	0.0013								
**34**	0.0099	0.0015	0.0087	0.0069	0.0081	0.0075	0.0066	-0.0023	0.0022	-0.0040	-0.0089	0.0020	-0.0019	0.0026									
**33**	0.0076	0.0032	0.0061	-0.0016	0.0015	0.0072	0.0033	-0.0010	0.0063	0.0016	-0.0051	0.0027	0.0027										
**31**	0.0073	0.0093	0.0043	-0.0007	0.0019	0.0054	0.0039	0.0070	0.0071	-0.0030	0.0016	0.0040											
**30**	0.0105	0.0037	0.0097	0.0059	0.0068	0.0085	0.0063	-0.0006	0.0046	-0.0029	-0.0054												
**26**	-0.0045	0.0053	-0.0066	-0.0122	-0.0076	-0.0083	-0.0031	0.0060	0.0000	-0.0147													
**22**	0.0018	-0.0028	-0.0028	-0.0103	-0.0051	-0.0007	-0.0044	-0.0088	-0.0027														
**16**	0.0055	0.0071	0.0031	-0.0011	0.0026	-0.0001	0.0051	0.0071															
**13**	-0.0037	0.0061	-0.0049	-0.0113	-0.0066	-0.0045	-0.0045																
**12**	0.0053	0.0000	0.0045	0.0006	0.0019	0.0056																	
**10**	0.0071	0.0001	0.0059	0.0054	0.0074																		
**8**	0.0048	-0.0033	0.0058	0.0020																			
**4**	0.0005	-0.0110	0.0030																				
**3**	0.0027	-0.0025																					
**2**	-0.0005																						

Table S6. Posterior distribution of the hyper parameters Bayesian sparse linear mixed models. The 95 % HPD regions of the hyper-parameters posterior distributions and the point estimate of the highest posterior density (HPD, from Fig. 4F and G of the main text). *PVE* is the proportion of phenotypic variance explained by all markers, GVE_β the proportion of PVE explained by the large, or sparse, effects and PVE_β the proportion of the phenotypic variation explained by large effect alleles. γ_N is the posterior distribution of the number of large effect. Point is the point along the x-axis with the highest density in y.

Full population set	2.50%	50%	97.50%	Point
PVE	0.478	0.580	0.681	0.584
GVE_β	0.147	0.357	0.663	0.328
PVE_β	0.087	0.206	0.373	0.190
γ_N	3	39	285	10
Reduced population set*				
PVE	0.427	0.557	0.690	0.555
GVE_β	0	0.316	0.898	0.034
PVE_β	0	0.100	0.807	0.010
γ_N	0	67	453	1

* Reduced population set is without population (10, 16, and 26) from the north western part of the distribution.

Figures

Figure S1

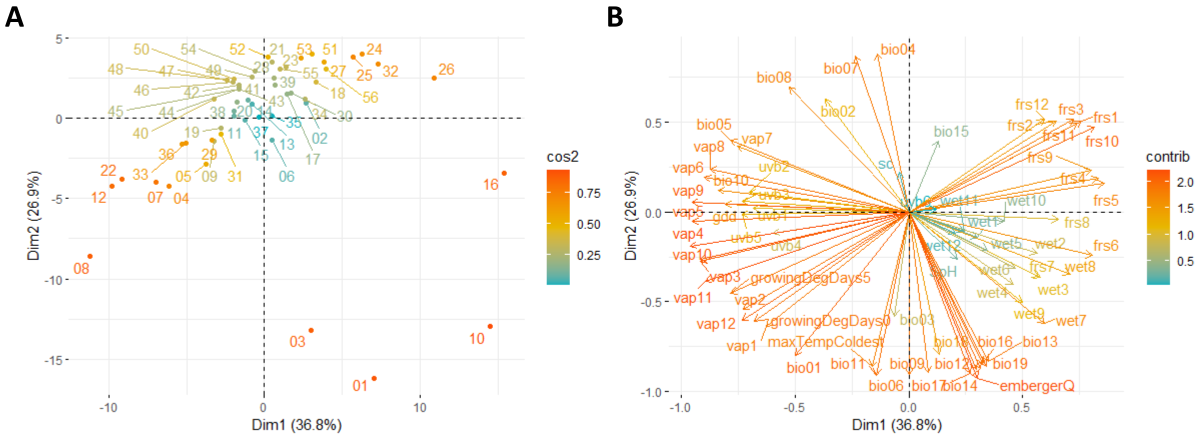

Figure S1. PCA of the environmental variables. (A) The populations' locations in the PCA-space. **(B)** The contributing variables and their direction in the first two PC-axis. Colors towards dark imply greater contribution.

Figure S2

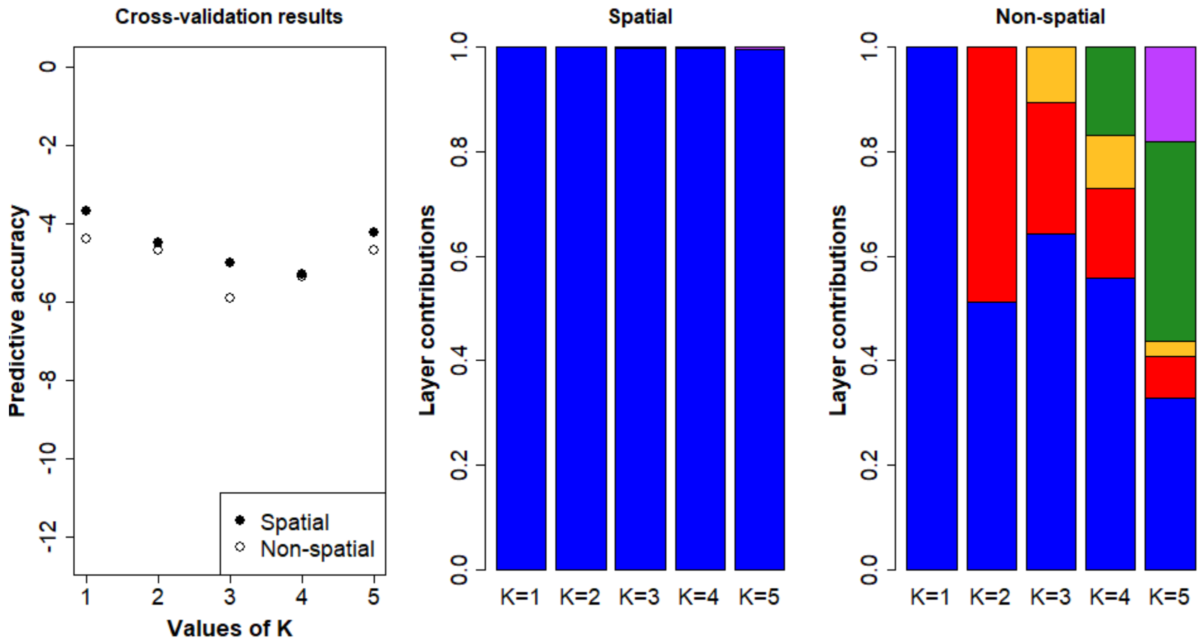

Figure S2. Cross-validation results and ancestral layer contributions to the total covariance with *K*=1 to *K*=5 for both the spatial and non-spatial model.

Figure S3

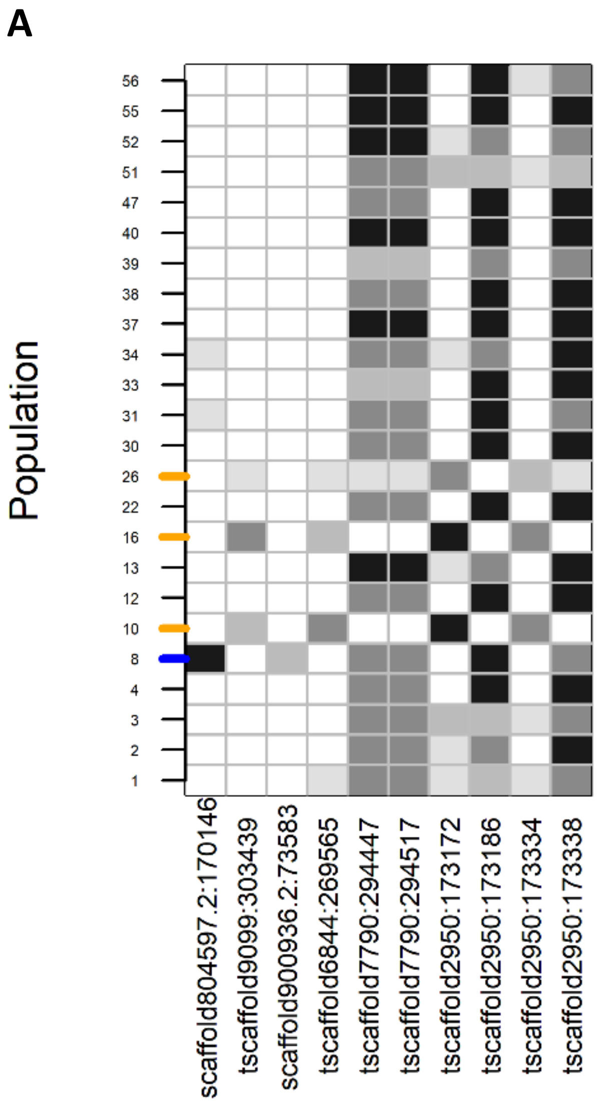

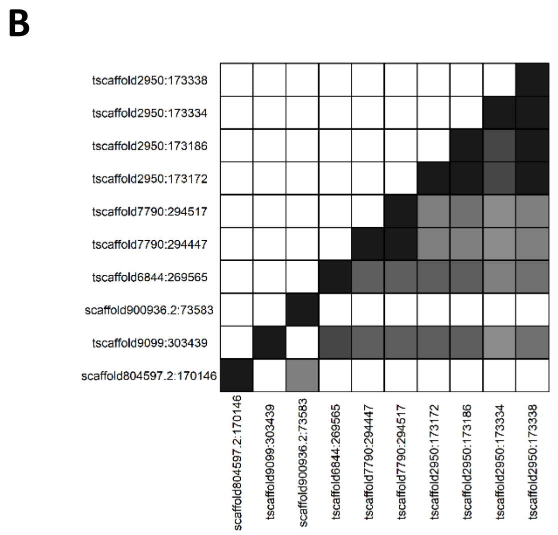

Figure S3. Population level linkage structure among ten of the most differentiated loci. **(A)** Orange tick marks indicate populations in the northwestern cluster and blue the southern cluster. Darker color indicate higher allele frequency or LD. Population ID on the y-axis and SNP on the x-axis. **(B)** LD (r^2) between the 10 loci

Figure S4

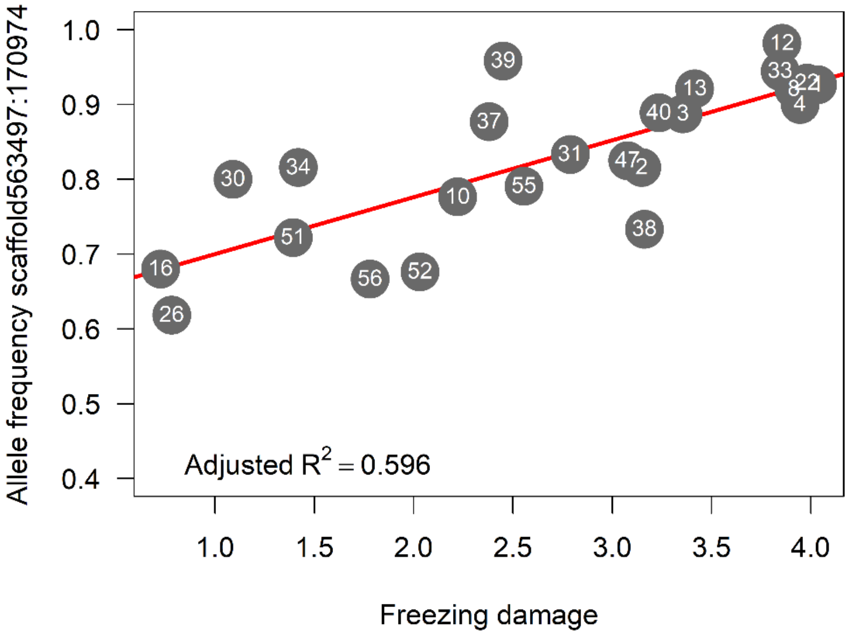

Figure S4. The allele frequencies of SNP scaffold563497:170974 as a function of freeze damage among populations, displayed as numbers within the points. Scaffold563497:170974 is the SNP that correlates most with freeze damage and is also highly correlated with the environmental variable vap2 but less with latitude or longitude see Figure 3D of the main text.
